# Transcriptomic responses in the oral cavity of F344 rats and B6C3F1 mice following exposure to Cr(VI): Implications for risk assessment

**DOI:** 10.1002/em.22064

**Published:** 2016-11-15

**Authors:** Chad M. Thompson, Julia E. Rager, Mina Suh, Caroline L. Ring, Deborah M. Proctor, Laurie C. Haws, Rebecca C. Fry, Mark A. Harris

**Affiliations:** ^1^ ToxStrategies, IncKatyTexas; ^2^ ToxStrategies, IncAustinTexas; ^3^ ToxStrategies, IncMission ViejoCalifornia; ^4^ Department of Environmental Sciences and EngineeringGillings School of Global Public HealthChapel HillNorth Carolina; ^5^ Curriculum in Toxicology, University of North Carolina at Chapel HillChapel HillNorth Carolina

**Keywords:** hexavalent chromium, mode of action, transcriptomics, risk assessment

## Abstract

Exposure to hexavalent chromium [Cr(VI)] in drinking water was previously reported to increase oral tumor incidence in F344 rats. To investigate the mode of action for these tumors, transcriptomic profiles in oral mucosa samples of F344 rats and B6C3F1 mice were analyzed following exposure to 0.1–180 ppm Cr(VI) for 7 or 90 days. In rats, genome‐wide microarray analyses identified no significantly differentially expressed genes (DEGs) at either time point. In mice, 14 and 1 DEGs were respectively identified after 7 and 90 days of exposure. Therefore, relaxed statistical criteria were employed to identify potential DEGs (pDEGs), followed by high‐throughput benchmark dose modeling to identify responsive pDEGs for pathway enrichment analysis. This identified 288 and 168 pDEGs in the rat oral mucosa, of which only 20 and 7 showed evidence of dose‐response. No significant pathway enrichment was obtained with either pDEG or dose‐responsive pDEG lists. Similar results were obtained in mice. These analyses indicate a negligible transcriptional response in the oral mucosa of both species. Comparison of the total number of gene changes in the oral mucosa of rats and mice with responses in the duodenum of animals from the same study demonstrated remarkable dose‐response concordance across tissues and species as a function of tissue chromium concentration. The low chromium levels in the oral mucosa and negligible transcript response are consistent with an absence of tissue lesions. These findings are used to compare the merits of linear and nonlinear approaches for deriving toxicity criteria based on the oral tumors in rats. Environ. Mol. Mutagen. 57:706–716, 2016. © 2016 The Authors. Environmental and Molecular Mutagenesis Published by Wiley Periodicals, Inc.

**Abbreviations**
AIC: Akaike information criterion
ANOVA: analysis of variance
BMD: benchmark dose
BMR: benchmark response
BPD: breakpoint dose
DAVID: Database for Annotation, Visualization and Integrated Discovery
DRC: dynamic reaction cell
FC: fold‐change
FDR: false discovery rate
HED: human equivalent doses
ICP‐MS: inductively coupled plasma‐mass spectrometr
MCL: maximum contaminant level
MF: mutant frequency
NTP: National Toxicology Program
PCA: principal component analysis
SDD: sodium dichromate dihydrate
TGR: transgenic rodent

## INTRODUCTION

Chromium from natural and anthropogenic sources is present in drinking water in trivalent [Cr(III)] and hexavalent [Cr(VI)] forms [Ellis et al., 2002; Oze et al., 2007; McNeill et al., 2012; U.S. EPA, 2014]. Average concentrations of Cr(VI) in U.S. drinking water supplies range from ∼0.0002 ppm [EWG, 2011] to 0.001 ppm [U.S. EPA, 2014]. The current U.S. maximum contaminant level (MCL) is 0.1 ppm total chromium [U.S. EPA, 1991; U.S. EPA, 1998], which is based on the absence of toxicity in Sprague‐Dawley rats exposed to 25 ppm Cr(VI) in drinking water for one year [Mackenzie et al., 1958]. More recently, the National Toxicology Program (NTP) reported that chronic exposure to ≥30 ppm Cr(VI) increased small bowel tumors (primarily in the duodenum) in B6C3F1 mice, and that exposure to 180 ppm increased oral mucosa tumors in F344 rats [NTP, 2008b; Stout et al., 2009].

Although the average Cr(VI) levels present in drinking water are well below the MCL, risk assessments based on the more recent NTP studies may result in new drinking water standards—depending on the toxicity endpoints, modes of action (MOAs), and quantitative dose–response approaches employed. As part of an investigation into the MOA of the tumors observed in the NTP cancer bioassay, 90‐day drinking water studies were conducted in F344 rats and B6C3F1 mice, with exposures ranging from 0.1 to 180 ppm Cr(VI) [Thompson et al., 2011; Thompson et al., 2012]. Animals in these studies were designated for either histopathological, pharmacokinetic, biochemical, or toxicogenomic evaluation. The two primary tissues of interest were the carcinogenic targets in the NTP cancer bioassay (small intestine and oral mucosa). In both rats and mice, robust dose‐dependent changes in gene expression occurred in the duodenum, particularly in mice at the higher exposure concentrations [Kopec et al., 2012a; Kopec et al., 2012b]. Based on mechanistic data in the small intestine, several groups have concluded that the MOA for intestinal tumors involves cytotoxicity and regenerative hyperplasia [Thompson et al., 2013; Becker et al., 2015; Haney, 2015; HealthCanada, 2015], and have developed threshold‐based toxicity criteria for these tumors [Thompson et al., 2014; Haney, 2015; HealthCanada, 2015; TCEQ, 2016].

The aforementioned Cr(VI) studies [Thompson et al., 2011; Thompson et al., 2012] also analyzed transcriptomic data from the oral mucosa, which were not published due to overall weak transcriptomic responses. However, two recent developments indicate that these oral mucosa transcriptomic data should be revisited. First, the oral mucosa (including the gingiva, palate, and buccal regions) was recently qualified for use in transgenic rodent (TGR) *in vivo* mutation assays to permit investigation of mutagenic potential in the oral cavity of Big Blue® TgF344 rats [Young et al., 2015]. Using the OECD 28 + 3 exposure protocol (i.e., 28 days of exposure followed by necropsy on day 31), the oral mutagenic carcinogen 4‐nitroquinoline‐1‐oxide (4NQO) increased mutant frequency (MF) 20‐ to 50‐fold in the oral mucosa, but not in liver or bone marrow [Young et al., 2015]. A subsequent study from the same laboratory again found that 4NQO increased MF in the oral mucosa, whereas exposure to 180 ppm Cr(VI) did not increase MF in the oral mucosa of TgF344 rats [Thompson et al., 2015].

The second development is that the NTP has a new initiative to analyze transcriptomic data from *in vitro* and *in vivo* studies, with the rationale that transcriptomic responses provide a link between exposure and apical effects (e.g., exposure and disease), and can assist in hazard identification and risk assessment [Merrick et al., 2015]. As such, transcriptomic responses in the oral mucosa might be more sensitive and informative than histopathology or TGR assays. On the other hand, negligible responses in a target tissue can still provide important information for hazard identification, MOA, and risk assessment, such as providing evidence for indirect and/or threshold mechanisms. Notably, histopathological analyses of the oral mucosa have revealed no non‐neoplastic or pre‐neoplastic lesions in either rats or mice after exposure to Cr(VI) for 7 days, 13 weeks, or 2 years [NTP, 2007; NTP, 2008b; Thompson et al., 2011; Thompson et al., 2012].

To further investigate the MOA for oral tumors and inform human health risk assessment for Cr(VI), we re‐analyzed the unpublished microarray data generated from previous 90‐day Cr(VI) drinking water studies [Thompson et al., 2011; Thompson et al., 2012]. Herein we describe the transcriptomic analyses from the oral mucosa of F344 rats and B6C3F1 mice exposed to ≤180 ppm Cr(VI) for 7 and 90 days. We also assessed the dose–response concordance across tissues and species by plotting gene expression changes as a function of tissue chromium burden. These data were then used to compare the merits of nonlinear and linear no‐threshold risk assessment approaches for the oral tumors in rats. The data and analyses herein should be informative for risk assessors evaluating the human health risks of oral exposure to Cr(VI) in drinking water.

## MATERIALS AND METHODS

### Test Substance

Rats and mice were exposed to Cr(VI) in the form of sodium dichromate dihydrate (SDD) as described previously [Thompson et al., 2011; Thompson et al., 2012]. In brief, SDD (CAS 7789‐12‐0; 99.95% pure) was obtained from Sigma‐Aldrich, Inc. (Milwaukee, WI) and stored at room temperature and protected from light. Dose formulations were prepared every two weeks at concentrations of 0.3, 4, 14, 60, 170, and 520 mg/L SDD in tap water, which is equivalent to ∼0.1, 1.4, 5, 20, 60, and 180 ppm Cr(VI). The latter four concentrations were employed in the NTP 2‐year cancer bioassay [NTP, 2008b]. Rats did not receive 14 mg/L SDD. The lowest concentration was selected because it is similar to the current U.S. EPA MCL [U.S. EPA, 1991; U.S. EPA, 1998]. On the first, third, fifth, and seventh (final) batch preparations, samples of formulations for each dose group, including the control, were collected and analyzed for Cr(VI) content at Brooks Rand Laboratories (Seattle, WA) in accordance with EPA Method SW‐7196A. Batches found to differ from the target concentration by ±10% were not used. Test article stability was previously determined by NTP [NTP, 2007]; dose formulations of SDD were stored in sealed Nalgene carboys at room temperature and protected from light.

### Test Animals

Details regarding animals, vendors, and animal husbandry were described previously [Thompson et al., 2011; Thompson et al., 2012]. The in‐life portion of these studies were conducted at Southern Research Institute (Birmingham, AL), which is the same research facility that conducted the NTP 13‐week and 2‐year Cr(VI) bioassays [NTP, 2007; NTP, 2008b]. Female Fischer 344 rats (Charles River Laboratories International, Inc.; Stone Ridge, NY) were received at ∼4 weeks of age and allowed to acclimate for ∼2 weeks. Rats weighed 83.1 to 126.4 g at the start of exposure. Female B6C3F1 mice (Charles River, Raleigh, NC) were received at 4–5 weeks of age and allowed to acclimate for ∼2 weeks. Mice weighed 13.3 to 22.9 g at the start of exposure. All animals were allowed *ad libitum* access to irradiated NTP‐2000 wafers (Zeigler Bros., Gardners, PA) and drinking water (including test article) until study termination at days 8 or 91 of the study. Water and Cr(VI) were supplied in amber glass water bottles with Teflon‐lined lids and stainless steel, double‐balled sipper tubes. Water bottles were changed twice weekly, or as needed. Before the start of the study, samples of tap water from the animal facility were analyzed, and no known contaminants were present that would be expected to interfere with or affect the outcome of the study.

The animals were group‐housed (five per cage) in solid bottom polycarbonate cages on a stainless steel rack in a room maintained at a temperature of 60.7°F–84.1°F and a relative humidity of 24.7–100%. Excursions outside the desired temperature (69°F–75°F) and humidity (35–65%) ranges were brief in duration and did not adversely affect the health of the animals or outcome of the study. Fluorescent lighting provided illumination approximately 12 h per day. Irradiated hardwood bedding chips (Sani Chips; P.J. Murphy Forest Products Corp., Montville, NJ) were used as bedding material. No known contaminants were present in the bedding that would have been expected to interfere with or affect the outcome of the study. Cage size and animal care conformed to the guidelines of the Guide for the Care and Use of Laboratory Animals, the U.S. Department of Agriculture through the Animal Welfare Act (Public Law 99‐198) and to the applicable SOPs of Southern Research.

Water and food consumption were measured weekly throughout the study, and values were reported as an average consumption (milliliters/animal per day or grams/animal per day, respectively). Animals were weighed the week before treatment began (for randomization), on day 1, weekly thereafter, and prior to scheduled euthanasia. All animals were observed for signs of mortality and moribundity at least twice daily during the pre‐study and study periods. Each animal was examined for clinical signs of toxicity on day 1 and weekly thereafter.

### Study Design

Details regarding study design were described previously [Thompson et al., 2011; Thompson et al., 2012] and are summarized in Supporting Information Figure S1 and Supporting Information Table I. Briefly, the study was designed to investigate the MOA for tumors observed in the NTP (2008) cancer bioassay on Cr(VI) by replicating exposure conditions for 13 weeks and collecting data from target tissues (i.e., duodenum and oral mucosa). Groups of 5 to 10 animals were randomly assigned to specific groups for analysis (histopathology, pharmacokinetics, toxicogenomics, biochemical, and mutation) prior to the start of the study. Animals were necropsied after either 7 or 90 days of exposure, and are referred to herein as either Day 8 and Day 91 groups. Because male and female mice responded similarly to Cr(VI) in the NTP (2008) study, only female mice were used in this study. The design of this study was informed by an expert panel (report is available at http://www.tera.org/Peer/Chromium/Chromium.htm). Data from this study are publicly available for download at www.cr6study.info.

### Measurement of Chromium in Oral Mucosa

Samples of the oral cavity and duodenum were collected for evaluation of total chromium (Cr) content following 90 days of exposure to Cr(VI). Five animals per group were anesthetized using CO_2_, and tissues were removed, flushed of contents, snap frozen, and stored at approximately 80°C. Samples were shipped frozen to Brooks Rand Laboratories where samples were thawed, lightly homogenized, and ∼100 mg of tissue was digested in nitric acid in a controlled microwave digestion program. Samples were then brought to a final volume of 8 ml with deionized water. Analysis was performed using EPA Draft Method 1638 (modified) using inductively coupled plasma‐mass spectrometry (ICP‐MS) with dynamic reaction cell (DRC) technology. Digested samples were analyzed utilizing internal standardization with rhodium. The limit of detection was 0.02 μg Cr/g tissue. Statistical analysis was conducted using Shirley's test [Shirley, 1977]. These tissue data were previously published [Thompson et al., 2011; Thompson et al., 2012], but are shown herein to further understand the transcriptomic responses.

### Transcriptomic Analyses

Oral tissue samples were prepared in a manner similar to those described previously for the small intestine [Kopec et al., 2012a; Kopec et al., 2012b]. Briefly, oral mucosa samples (*viz*. upper hard and soft palate) from behind the upper front teeth back to the soft palate were placed into vials containing ∼1 mL of TRIzol (Invitrogen, Carlsbad, CA), snap‐frozen in liquid nitrogen, stored at −80°C, and total RNA was extracted following manufacturer's protocol. Only high‐quality RNA samples (A260/280 > 1.9) were examined using Rat and Mouse 4 × 44 K Agilent whole‐genome oligonucleotide microarrays (version 1, Agilent Technologies, Inc., Santa Clara, CA) as described previously [Kopec et al., 2012a; Kopec et al., 2012b]. The tissue samples and microarrays were processed shortly after collection (i.e., *ca*. 2012). Microarray data are publicly available at www.Cr6study.info and NCBI's Gene Expression Omnibus [Edgar et al., 2002], accessible through GEO Series accession number GSE87262.

Microarrays incorporated independent labeling of each sample with two different dyes that were scanned using a GenePix 4000B scanner at 532 nm for Cy3 labeling (green dye), and 635 nm for Cy5 labeling (red dye). The mRNA microarray data were analyzed by pulling data for the median Cy3 dye signal, because the Cy5 dye signal has been shown to be susceptible to laboratory‐introduced biases/errors (e.g., ozone) [Fare et al., 2003]. Data were then normalized by quantiles (Partek Genomics Suite, St Louis, Missouri) and assessed for overall quality through microarray expression value distribution assessment (i.e., comparison of mean, median, minimum, and maximum signal intensities) and principal component analysis (PCA). PCA was conducted using the prcomp() function in R (RStudio v0.99.489) with the first three components plotted using the scatterplot3d() function. Background noise was eliminated by removing mRNA probes with signal intensities less than the median signal across the majority of samples. Significantly differentially expressed genes (DEGs) were defined as those with a significant difference in mRNA levels between exposed (*n* = 3) versus unexposed (*n* = 3) samples, where three statistical requirements were set: (1) fold change of ≥2 or ≤−2 (average exposed vs. average unexposed), (2) analysis of variance (ANOVA) *P* < 0.05, and (3) a false discovery rate (FDR) corrected q <0.05. These methods and statistical parameters are similar to those implemented in recent publications using Agilent array data [Bartman et al., 2014; Rager et al., 2014]. Furthermore, these methods and overall design, including the use of biological triplicates, are in line with current guidelines for microarray analyses [Bourdon‐Lacombe et al., 2015].

An additional analysis was carried out through the evaluation of microarray data uploaded on NCBI's GEO website using the GEO2R data analysis pipeline (https://www.ncbi.nlm.nih.gov/geo/info/geo2r.html). GEO2R is a web tool that allows users to statistically compare gene expression profiles between sample groups using the GEOquery and Linear Models for Microarray Analysis (limma) R packages. Default statistical values were generated, including adjusted *P*‐values based off the Benjamini & Hochberg FDR method. Genes (represented by probesets) were required to have adjusted *P* < 0.05 (comparing exposed vs. unexposed samples) in order to be identified as significantly differentially expressed.

Microarray results from the small intestine were also used in this evaluation, with the goal of comparing tissue Cr levels against the number of DEGs across multiple tissues. Microarray data from the small intestine have been processed, analyzed, and published previously using different methods [Kopec et al., 2012a; Kopec et al., 2012b]. To ensure consistency in the current cross‐tissue analysis, microarray data from the small intestine were re‐analyzed here using the same updated methods described above for the oral mucosa analysis.

### Evaluating Dose‐Response Concordance Across Tissues and Species

The number of significant DEGs in the oral mucosa and duodenum were plotted against tissue Cr levels, and the potential relationship was evaluated with bi‐linear modeling using the *segment* [Muggeo, 2008] function of the *drsmooth* [Hixon et al., 2015] package for R (http://www.R-project.org). To model these data, replicate values per dose were required to establish levels of variance. As such, the number of DEGs for each exposed animal was estimated by evaluating the significant DEGs and summing those with fold‐change (FC) values ≥2 per animal (where FC = individual exposed animal/average unexposed animals). There were 3 biological replicates per dose group with the exception of only 2 replicates in one low‐dose mouse duodenum group. These calculations resulted in a range for the number of DEGs that could be modeled for each tissue Cr dose. Note that the number of DEGs per animal, in some instances, differed from the total number of DEGs per exposure group.

### Transcriptomic Analysis Using Relaxed Statistical Criteria

**Figure 1:**
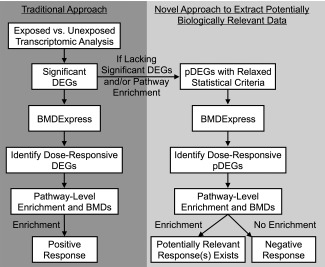
Flowchart summarizing steps conducted in more traditional transcriptomic analyses estimating pathway‐level BMDs (left side) versus steps conducted in the current study to search for potentially relevant responses using less stringent statistical criteria (right side). The new approach relaxes statistical stringency to identify potential DEGs (pDEGs) in exposed versus unexposed samples, and then filters these for pDEGs showing dose‐dependent changes. Pathway analysis of these pDEGs can either identify potentially biologically relevant changes in signaling or confirm overall negative transcriptomic responses.

Current guidelines for gene expression profiling in the field of human health risk assessment encourage the use of data adjustment for false positives, wherein FDR adjustment is specified as a commonly used approach [Bourdon‐Lacombe et al., 2015]. For the current dataset, transcriptomic responses were extremely minimal using these traditional filters. In order to ensure that any potentially relevant findings were not overlooked, a new approach (Fig. 1) was employed in which potential DEGs (pDEGs) were identified using relaxed statistical criteria and then further analyzed for potential dose‐dependent responses to Cr(VI). The following two requirements were set to identify pDEGs: (1) fold change of ≥1.5 or ≤−1.5 (average exposed vs. average unexposed), and (2) ANOVA *P* < 0.05 with no FDR *q*‐value filter used.

Dose–response modeling was conducted with BMDExpress v1.4 using methods previously detailed [Yang et al., 2007; Moffat et al., 2015]. For the relatively weak transcript responses in this study, the aim of using BMDExpress to evaluate pDEGs was not to derive BMD values *per se*, but rather to use BMDExpress to identify genes potentially showing dose‐dependent responses to Cr(VI). Briefly, analyses were carried out on each list of pDEGs (represented by their respective microarray probesets) against SDD treatment concentration using four models: Hill, power, linear, and 2° polynomial. The models were run assuming constant variance, and a benchmark response (BMR) factor of 1.349 was used, representing the number of standard deviations required to shift the mean transcriptional response 10% over the assumed background rate of response, defining the benchmark dose (BMD). Models with the best fit were selected as those that (1) described the data with the least complexity, (2) had a nested likelihood ratio test *P* < 0.05, and (3) had the lowest Akaike information criterion (AIC). The Hill model was considered only when the k parameter was more than one third of the lowest dose tested, in order to avoid artificial minimization of BMDs. Other parameters used in the analysis included setting the maximum iterations to 250 and the confidence level to 0.95. Probesets were removed if they showed either BMD/BMDL (BMD lower confidence limit) ratios > 20, BMD values less than one third of the lowest SDD concentration tested, and/or BMD values greater than the highest concentration tested in order to avoid model extrapolation. In order for the models to adequately describe potential dose‐response trends in gene expression, curves were required to have goodness‐of‐fit *P* > 0.1 (likelihood ratio test) [Yang et al., 2007; Moffat et al., 2015]. Pathway enrichment analysis of the pDEGs showing dose–response relationships with SDD exposure was carried out using the Database for Annotation, Visualization and Integrated Discovery (DAVID) (v6.7) by querying the Kyoto Encyclopedia of Genes and Genomes (KEGG) pathway database [Huang et al., 2009]. Pathways with enrichment *P* < 0.05 were identified as associated with dose‐responsive pDEGs.

### Dose‐Response Modeling of Oral Tumors in F344 Rats

Dose–response modeling of oral tumor data was conducted with U.S. EPA's BMDS v2.6, and in accordance with U.S. EPA guidelines [U.S. EPA, 2012]. The estimated Cr(VI) study doses in rats [mg/kg Cr(VI)] were converted to human equivalent doses (HED) by allometric scaling prior to BMD modeling, consistent with U.S. EPA recommendations [U.S. EPA, 2011]. These HEDs along with corresponding tumor incidence data from the NTP 2‐year bioassay [NTP, 2008b] were modeled using the 1^st^, 2^nd^, and 3^rd^ degree multistage cancer models with a BMR of 10% extra risk. The model with lowest AIC and acceptable goodness‐of‐fit *P*‐value was selected.

## RESULTS

### Chromium Dosimetry in the Oral Mucosa and Duodenum

**Figure 2:**
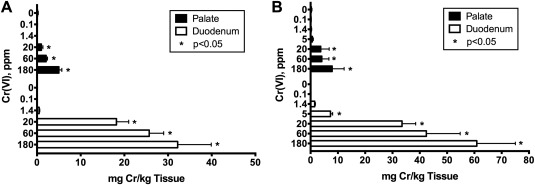
Oral and duodenal tissue chromium levels in rats (**A**) and mice (**B**) exposed to ≤180 ppm Cr(VI) in drinking water for 90 days. Data represent mean and SD (*n* = 5). These data are adapted from previous publications [Thompson et al., 2011; Thompson et al., 2012].

Tissue dosimetry data following 90 days of exposure to 0.1–180 ppm Cr(VI) in drinking water were published previously [Thompson et al., 2011; Thompson et al., 2012]; the tissue burden in the oral cavity and duodenum of both rats and mice are shown in Figure 2. Tissue chromium levels in the oral mucosa were increased significantly in rats and mice exposed to ≥20 ppm Cr(VI) for 90 days (Fig. 2). Exposure to ≤5 ppm Cr(VI) for 90 days did not significantly increase chromium levels in the oral mucosa of either species. Tissue Cr levels were much higher in the duodena of the same mice and rats, reaching up to 61 mg/kg (Fig. 2).

### Significant DEGs as a Function of Tissue Burden

**Figure 3:**
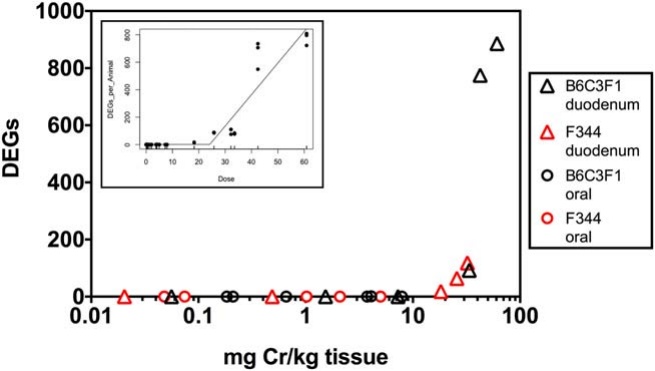
Differential gene expression as a function of tissue dose after 90 days of exposure to Cr(VI) in B6C3F1 mice and F344 rats. Tissue chromium levels in the oral mucosa and duodenum, are plotted against the number of significant DEGs in the oral mucosa and duodenum. The inset plot displays bilinear modeling of DEGs indicating a potential threshold at 22.0 mg Cr/kg tissue.

Using standard statistical criteria (e.g., multiple test correction and ≥±2‐fold change in expression), the number of DEGs were plotted as a function of tissue chromium levels in the duodena and oral mucosae of rats and mice following 90 days of exposure (Fig. 3). This plot demonstrates strong dose–response concordance across species and across tissues. Tissue chromium levels below ∼10 mg/kg elicited minimal, if any, transcriptomic responses in the oral mucosa or duodenum. Duodenal chromium levels greater than ∼10 mg/kg elicited transcriptomic responses in both rats and mice (Fig. 3). Bilinear modeling of these data indicated a bilinear breakpoint dose (BPD) and lower confidence limit (BPDL) at 24.1 and 22.0 mg Cr/kg tissue, respectively (inset of Fig. 3).

### Significant DEGs in the Oral Mucosa

To further investigate transcript changes in the oral cavity, DEGs were analyzed in rats and mice after both 7 and 90 days of exposure. Quality assessment showed that all microarray expression values had similar distributions across samples (i.e., similar mean, median, minimum, and maximum values). All microarray expression values also showed similar PCA groupings, with the potential exception of one microarray from the lowest exposure concentration (0.3 mg/L SDD) from the rat day 91 group (Supporting Information Fig. II). Although this sample showed similar expression value distributions, projecting its data through PCA onto lower dimensional subspaces suggested that it may be an outlier. It is not possible to identify the cause of this potential outlier, as all RNA samples evaluated were of high quality and were analyzed using the same amount of starting RNA material, among other potential experimental variables that were controlled for. It is important to note that microarray outliers may simply be the result of natural data variability, and that discarding samples on the basis that they are outliers without more substantive explanations is highly discouraged [Shieh and Hung, 2009]. Still, analysis of differential gene expression with and without this sample resulted in the same findings (detailed below).

**Figure 4:**
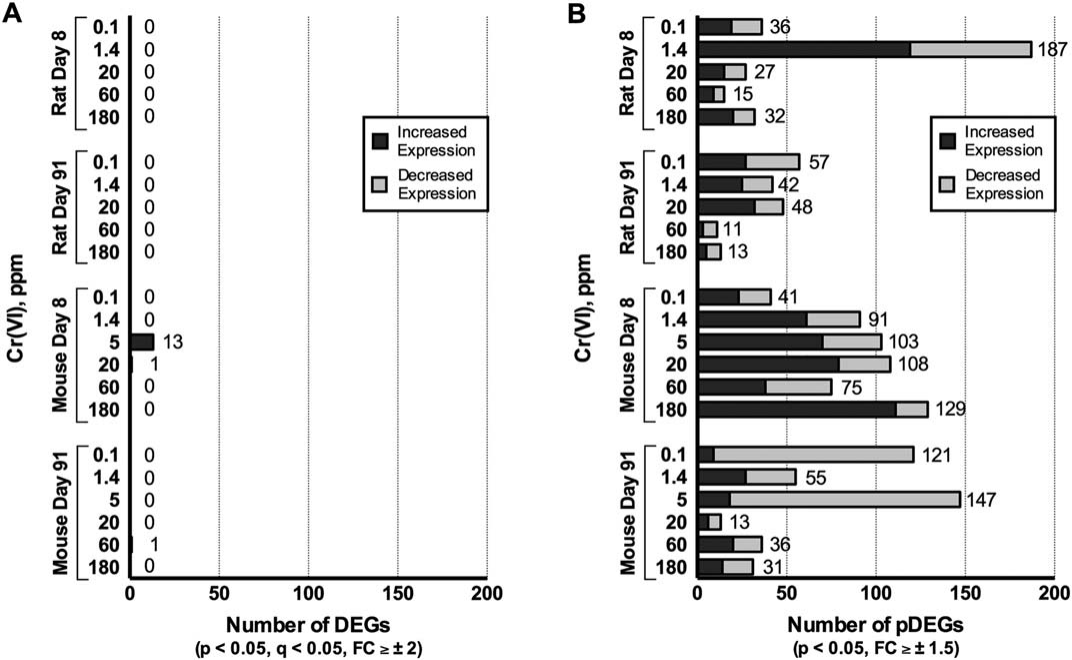
Transcriptomic responses in the rat and mouse oral mucosa following exposure to ≤180 ppm Cr(VI) for 7 and 90 days. Statistically significant DEGs are shown in (**A**), and because of the minimal responses, relaxed filters were used to identify pDEGs for further evaluation, as shown in (**B**). Data represent 3 biological replicates.

Using standard statistical criteria (e.g., multiple test correction and ≥±2‐fold change in expression), no significant DEGs were identified at any dose in rats after either 7 or 90 days of exposure in the oral mucosa; only one probeset showed differential expression that was not annotated to a known rat gene (Fig. 4A, Supporting Information Table II). Similar results were obtained through an additional statistical analysis using NCBI's GEO2R online tool, with no genes showing significant differential expression resulting from Cr(VI) exposure (i.e., all probesets had adjusted *P* > 0.05).

In mice, 14 significant DEGs were identified at day 8 (in at least one dose group), and only one significant DEG was identified at day 91 (Fig. 4A, Supporting Information Table II). Due to the low number of gene changes, functional enrichment analysis could not be conducted at this stage; however, enrichment analysis was carried out during the analysis of the pDEGs (see below). An additional statistical analysis through GEO2R identified three significant DEGs at day 8, specifically activating transcription factor 3 (*Atf3*), early growth response 1 (*Egr1*), and FBJ osteosarcoma oncogene (*Fos*), all of which overlapped with those identified in the first statistical analysis (Supporting Information Table II). Similarly, no significant DEGs were identified at day 91 through this additional analysis.

### Analysis of pDEGs in the Oral Mucosa

To extract any potentially relevant changes in the oral mucosa, a new approach was employed to identify pDEGs (through relaxed statistical criteria) and determine whether pDEGs show dose‐dependent changes with Cr(VI) that are relevant to canonical pathways. Statistical criteria were relaxed by eliminating the multiple test correction (FDR *q*‐value) requirement and decreasing the fold change in expression requirement from ≥±2 to ≥±1.5. In the rat oral mucosa, relaxing the criteria resulted in the identification of 288 and 168 pDEGs (at one or more doses) at day 8 and day 91, respectively (Supporting Information Table III). In mice, using the more relaxed criteria resulted in a total of 446 and 294 pDEGs at day 8 and day 91, respectively (Supporting Information Table III). For the results in both rats and mice, the number of pDEGs did not consistently increase with increasing dose (Fig. 4B).

To further explore these putative changes in gene expression, the pDEGs were analyzed for dose–response relationships using BMDExpress. BMDExpress was not used here for the purpose of deriving BMD values; instead, the dose–response modeling algorithms within BMDExpress were leveraged to search through the pDEGs and identify genes showing dose–response relationships with Cr(VI). Of the 288 and 168 pDEGs identified in the rat oral mucosa, only 20 and 7 pDEGs showed curves with goodness‐of‐fit *P* > 0.1 after 7 and 90 days of exposure, respectively (Supporting Information Table IV). Of the 446 and 294 pDEGs in the mouse oral mucosa, 36 and 31 pDEGs showed curve fit *P* > 0.1 after 7 and 90 days of exposure, respectively (Supporting Information Table IV). It should be noted that some fraction of pDEGs will likely have acceptable *P*‐values for fit by chance, i.e., do not represent biological dose–responses. For example, ∼50% of the dose‐responsive pDEGs in the rat oral mucosa were linear models, and examination of some of these models indicated that the slopes did not differ significantly from zero. Nevertheless, the pDEGs showing good curve fits were analyzed for KEGG pathway enrichment. No pathways were identified as being associated with these lists of pDEGs showing dose–response trends in expression.

Considering that these pDEGs did not pass FDR correction criteria, coupled with the overall lack of dose–response relationships/pathway enrichment for the pDEGs, this analysis of potential changes in gene expression clearly shows that Cr(VI) exposure causes minimal changes in transcriptional levels in the oral mucosa of rodents at exposure doses up to 180 ppm and durations up to 90 days.

### Dose–Response Modeling of Oral Tumors in F344 Rats

**Figure 5:**
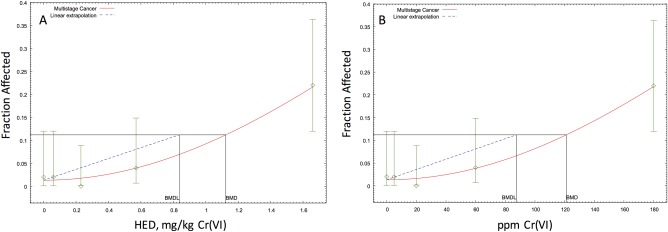
BMD modeling of oral tumor incidence in female F344 rats based on human equivalent doses (mg Cr(VI)/kg) (**A**) and mg/L Cr(VI) (**B**). The *P*‐value for model fits in A and B were 0.75 and 0.77, respectively. Tumor incidence data is from 50 rats per group [NTP, 2008b].

Modeling of the oral tumor incidence in female rats of the NTP 2‐year cancer bioassay results in a BMDL_10HED_ of 0.84 mg Cr(VI)/kg (Fig. 5A); a higher BMDL_10HED_ was obtained for male rats (data not shown). This results in an oral slope factor of 0.12 per mg/kg, and a 10^−6^ risk‐specific dose of 8.3E‐6 mg/kg. By comparison, application of similar uncertainty factors as applied by the U.S. EPA in their draft assessment [U.S. EPA, 2010] results in an RfD of 0.03 mg/kg‐day (0.84 mg/kg‐day ÷ 30). Based on the assumption of a 80 kg adult consuming 2.5 L of water per day, the linear and nonlinear extrapolation methods used to derive toxicity criteria result in drinking water concentrations of approximately 0.0003 ppm and 1 ppm, respectively. Thus, applying linear and nonlinear extrapolation approaches to the same data yields drinking water estimates that differ by four orders of magnitude.

An alternative to the mg/kg dose metric (above) for the oral tumors might simply be Cr(VI) concentration in drinking water. BMD modeling of the same tumor incidence data using ppm Cr(VI) as the dose metric results in BMD_10_ and BMDL_10_ of 121 and 87.5 ppm, respectively (Fig. 5B). With this dose metric, allometric scaling is unnecessary, and thus, a nonlinear approach to deriving safe drinking water concentrations would result in 2.9 ppm (i.e., 87.5 ppm divided by a 30‐fold uncertainty factor). By contrast, a slope factor would be 0.0011 ppm^−1^, which results in a 10^−6^ risk‐specific dose of 0.0009 ppm Cr(VI).

## DISCUSSION

This study is the first to evaluate potential transcriptomic changes in the oral mucosa associated with exposure to Cr(VI) in drinking water. The main finding from this study is that exposures to ≤180 ppm Cr(VI) for 90 days resulted in minimal changes in gene expression in the oral mucosa of rats or mice. This conclusion is based on multiple lines of evidence. First, two separate statistical approaches both found zero significant DEGs at any dose or exposure duration in the rat, the species that developed oral tumors in the NTP 2‐year cancer bioassay [NTP, 2008b]. In mice, 14 significant DEGs were identified only at the mid‐treatment levels at day 8; no significant DEGs were identified in mice at day 91. Second, when an approach was used in an attempt to identify any potentially relevant changes, relaxed statistical filters identified a subset of pDEGs in the oral mucosa that showed acceptable dose–response curve fits, but these genes were not enriched for any canonical pathways. Together, transcriptomic analyses clearly show that Cr(VI) exposure via drinking water does not cause substantial changes in transcriptional profiles in the oral mucosa of rats and mice.

The lack of transcript response was consistent with the low chromium concentrations in oral tissue. Below ∼10 mg/kg tissue concentration, there were few gene changes in the oral mucosa or small intestine of either species; however, once tissue levels exceeded 10 mg/kg, there were robust changes in gene expression in the duodenum. Indeed, bilinear modeling of DEGs, using methods similar to those described for modeling genetic toxicity data [Johnson et al., 2014], indicated a potential threshold at 22.0 mg Cr/kg tissue. These data further support the observation that tissue absorption above ∼10 mg/kg is likely required to elicit substantial *in vivo* transcriptomic changes.

The general lack of transcriptional changes in the oral mucosa is entirely consistent with previous histopathology data indicating no non‐neoplastic or pre‐neoplastic changes in the oral mucosa of rats or mice [NTP, 2007; NTP, 2008b; Thompson et al., 2011; Thompson et al., 2012]. Likewise, exposure to 180 ppm Cr(VI) did not increase *cII* transgene mutant frequency in TgF344 rats [Thompson et al., 2015]. To date, the only dose‐dependent changes in the oral mucosa in response to Cr(VI) were decreases in GSH and increases in GSSG levels in rats, which were not apparent in mice [Thompson et al., 2011; Thompson et al., 2012]. These new transcriptomic findings suggest that the changes in GSH/GSSG were not in the oral tissue *per se*, but rather, occurred in the saliva that continuously bathes the oral mucosa [Nagler and Dayan, 2006]. It is conceivable that changes in saliva redox status might influence oral health in rats over a chronic exposure scenario.

We suggested previously that the tumors in rats were likely secondary to other adverse effects [Suh et al., 2014]. In the NTP study, water intake was reduced by ∼5 mL per day in the rats in the 180 ppm groups, likely due to poor palatability. Bodyweight was also reduced. Iron levels were decreased in serum, liver, and duodenum, and transcript changes in the duodenum were consistent with iron deficiency [Suh et al., 2014]. Reduced water intake can affect salivary volume and secretion [Fischer and Ship, 1997; Ito et al., 2001], and saliva can affect oral health [Wallenius, 1966; Smiler, 1970; Nagler and Dayan, 2006; Vered et al., 2010]. Iron deficiency may potentially be linked to oral tumors in rats and oral cancers in humans [Prime et al., 1983; Richie et al., 2008], perhaps due to effects of iron deficiency on saliva composition [Johansson and Fagernas, 1994]. Although the data herein do not provide support for a specific MOA underlying the oral tumors in rats, the transcriptomic and chromium disposition data argue strongly against a MOA involving direct action of Cr(VI) in the oral tissue, seemingly precluding both direct cytotoxic and genotoxic MOAs.

The findings herein provide important information for regulators assessing the oral toxicity and carcinogenicity of Cr(VI). Transcriptomic responses have been argued to provide sensitive sentinel responses to chemicals; studies indicate that transcript changes are ∼2‐fold more sensitive than apical/frank effects [Thomas et al., 2013]. The lack of transcript changes in the oral mucosa suggests that Cr(VI) did not impart any effect (direct or indirect) on the mucosa after 90 days of exposure. Importantly, these transcriptomic findings are phenotypically supported by the low chromium tissue concentration and the lack of histopathological or genotoxic responses in the oral cavity. These data indicate that the oral cavity tumors in rats were unlikely to be mediated at the site of contact.

If the oral tumors in rats were the result of systemic effects, they were unlikely due to Cr(VI) or Cr(III) delivery to tissue via the blood. Once absorbed into the bloodstream, Cr(VI) is rapidly reduced to Cr(III) by blood constituents or taken into blood cells and reduced therein. Although the high Cr(VI) concentrations employed in the 2‐year cancer bioassays might have allowed limited systemic delivery of Cr(VI), this would be unlikely to occur at environmentally relevant levels where Cr(VI) is efficiently reduced to Cr(III) by gastric fluid [Proctor et al., 2012; De Flora et al., 2016; Kirman et al., 2016]. Cr(III) could reach the oral mucosa via the bloodstream, but Cr(III) is not carcinogenic to rats or mice [NTP, 2008a]. Considering that neither Cr(VI) nor Cr(III) appear to affect the oral mucosa, the apparent association between Cr(VI) exposure and oral tumor formation likely involves indirect mechanisms, and thus would be incompatible with a linear MOA and linear low‐dose extrapolation methods for setting toxicity criteria. Table 1 summarizes relevant oral mucosa data from rats that support nonlinear low‐dose extrapolation approaches.

**Table 1:** Summary of Effects in the Rat Oral Mucosa

Endpoint	Evidence
Cr(VI) tissue absorption	‐ No significant increases in tissue Cr concentrations following 90 days of exposure to ≤5 ppm Cr(VI) (Fig. 2)
	‐ Significant increases in tissue Cr concentrations following 90 days of exposure ≥20 ppm Cr(VI) (Fig. 2)
Histopathology	‐ No non‐neoplastic or pre‐neoplastic histopathological lesions have been detected in the rat oral mucosa following exposure to ≤180 ppm Cr(VI) for 7 days [Thompson et al., 2012], 13 weeks [Thompson et al., 2012; NTP, 2007], or 2 years [NTP 2008b, Stout et al., 2009]
Biochemical analyses	‐ Decrease in GSH and increase in GSSG at ≥60 ppm Cr(VI) [Thompson et al., 2012]; effects might be due to changes in saliva
Mutation analysis	‐ No increase in mutant frequency in the oral mucosa of Big Blue® TgF344 rats following exposure to 180 ppm Cr(VI) in drinking water [Thompson et al., 2015]
Transcriptomic analyses	‐ No significant DEGs, or pathways associated with dose‐responsive pDEGs, associated with exposure to ≤180 ppm Cr(VI) in drinking water for 7 and 90 days (Fig. 4)

The incompatibility of conducting low‐dose linear extrapolation from the oral tumors is highlighted by the vast difference in safe drinking water levels estimated from BMD modeling of the rat oral tumor data. Based on allometric scaling, the estimated human equivalent 10^−6^ risk‐specific dose results in a drinking water concentration of ∼0.0003 ppm. To put 0.0003 ppm into perspective, chromium levels did not significantly increase in the oral mucosa tissue of rats and mice until >5 ppm, and no significant transcript changes were evident after exposure to 180 ppm. Thus, the notion that “safe” exposure to Cr(VI) (based on oral tumors in rats) occurs only at ≤0.0003 ppm Cr(VI) is incompatible with available pharmacokinetic and pharmacodynamic data for Cr(VI). Using nonlinear extrapolation approaches from the same underlying data results in a drinking water concentration of ∼1 ppm, which is higher than the current MCL for total chromium of 0.1 ppm [U.S. EPA, 1991]. The 1 ppm value is more consistent with pharmacokinetic data indicating that tissue chromium levels begin to increase somewhere above 5 ppm (Fig. 2), and toxicological data indicating no transcript, histopathologic, or genotoxic changes. These new data and analyses indicate that, if oral tumors in rats are to be considered for setting safety criteria for Cr(VI) in drinking water, nonlinear approaches are scientifically justifiable.

Finally, these data have important broad implications for risk assessment. Specifically, negative data are important data. High content transcriptomic data have been argued to provide invaluable and sensitive data for risk assessment purposes [Wilson et al., 2013; Bourdon‐Lacombe et al., 2015]. Although most frequently used to investigate exposures (e.g., environmental, pharmaceutical) that result in tissue and cellular effects (i.e., *positive* responses), a less common application of transcriptomic data is to identify exposures that do not elicit tissue effects (i.e., *negative* responses). The present study demonstrates both applications, wherein positive responses resulting from Cr(VI) exposure are apparent in the intestine and negative responses in the oral mucosa. The consistent lack of transcript response to Cr(VI) in the oral mucosa *vis‐à‐vis* the intestine provide important information for risk assessment—especially considering the consistency of transcript responses with phenotypic anchors of histopathology, pharmacokinetics, and genotoxicity.

As with any study, there are potential limitations. Great care was taken to replicate the NTP study including: use of the same contract research laboratory, same feed, same strains of rodents, same Cr(VI) concentrations, analytical verification of test solutions, rapid storage and preparation of tissue samples, and transcriptomic analyses consistent with the most recent recommendations [Bourdon‐Lacombe et al., 2015]. Toxicogenomic analysis from more animals (i.e., more replicates) may have resulted in slightly different numbers of DEGs or pDEGs. However, we have shown consistent results across four dose‐response datasets (mice and rats at day 8 and 91) with a wide range of Cr(VI) concentrations, including concentrations carcinogenic to rodents. Moreover, the minimal transcript responses are consistent with tissue Cr levels.

The data herein, coupled with previous *in vivo* target tissue data indicate nonlinear risk assessment approaches are scientifically justifiable if oral tumors are to serve as the basis for toxicity criteria for Cr(VI) in drinking water.

## AUTHOR CONTRIBUTIONS

Mark Harris, Laurie Haws, and Chad Thompson designed the in‐life portion of the study. Julia Rager, Rebecca Fry, Caroline Ring, and Chad Thompson conducted these new toxicogenomics analyses. Chad Thompson and Julia Rager prepared draft manuscript, figures and tables, with important intellectual contributions from Mina Suh, Deborah Proctor, Laurie Haws, Mark Harris and Rebecca Fry. All authors approved the final manuscript.

## Supporting information

Supporting Information
