# Supplementary material for: Transcriptomic responses in the oral cavity of F344 rats and B6C3F1 mice following exposure to Cr(VI): Implications for risk assessment
Source: Environ Mol Mutagen. 2016 Nov 15;57(9):706–16. doi: 10.1002/em.22064 (PMC5215477; doi:10.1002/em.22064)
Supplement: Supplementary file 1 — Supporting Information [file EM-57-706-s001.docx]

**Supplementary Material**

**Transcriptomic Responses in the Oral Cavity of F344 Rats and B6C3F1 Mice Following Exposure to Cr(VI): Implications for Risk Assessment**

Chad M. Thompson, Julia E. Rager, Mina Suh, Caroline L. Ring, Deborah M. Proctor,

Laurie C. Haws, Rebecca C. Fry, and Mark A. Harris

**Supplementary Material**

**Table of Contents**

**Supplementary Figure I.** Summary of study design and study history………………........ **Page 1**

**Supplementary Figure II.** Principal component analysis (PCA) of microarray data…….. **Page 2**

**Supplementary Table I.** Summary of Study Design…………………………………........ **Page 3**

**Supplementary Table II.** Significant differentially expressed probes (DEPs) representing differentially expressed genes (DEGs) associated with Cr(VI) exposure in the oral mucosa of the rats and mice……………………………………………………………………………....... **Page 4**

**Supplementary Table III.** Potential differentially expressed probes (pDEPs) representing potential differentially expressed genes (pDEGs) associated with Cr(VI) exposure in the oral mucosa of the rats and mice………………………………………………………………... **Page 5**

**Supplementary Table IV.** BMDExpress dose-response results for potential differentially expressed genes (pDEGs) with goodness-of-fit p > 0.1…………………………………... **Page 50**

**Supplemental Figure 1. Summary of study design and study history.**

**
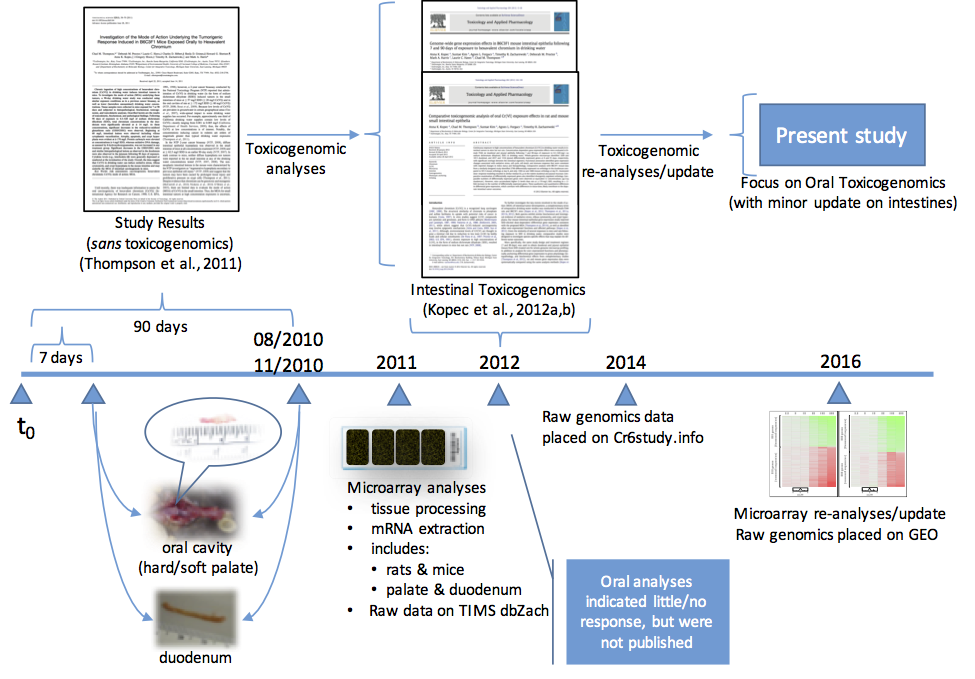
**

**Supplementary Figure II. Principal component analysis (PCA) of microarray data.** PCA plots are shown for all microarray data for the following sample groups: (**A**) Mouse palate day 8 samples, (**B**) Rat palate day 8, (**C**) Mouse palate day 91, (**D**) Rat palate day 91, (**E**) Mouse palate day 8 and 91 combined, and (**F**) Rat palate day 8 and 91 combined.

**Supplementary Table I. Summary of study design.**

|  | **Number of Animals** | | | | | | | |
| --- | --- | --- | --- | --- | --- | --- | --- | --- |
| **SDD**  **(mg/L)** | **Toxicology & Histopathology** | | **Biochemical Evaluations** | | **Gene Expression Analyses^3^** | | **Mutation Analyses** | **Toxicokinetic Analyses** |
|  | **Day 8** | **Day 91** | **Day 8** | **Day 91** | **Day 8** | **Day 91** | **Day 91** | **Day 91** |
| 0 | 5 F | 10 F | 10 F | 20 F | 10 F | 10 F | 10 F | 5 F |
| 0.3 | 5 F | 10 F | 10 F | 20 F | 10 F | 10 F | 10 F | 5 F |
| 4 | 5 F | 10 F | 10 F | 20 F | 10 F | 10 F | 10 F | 5 F |
| 14^1,2^ | 5 F | 10 F | 10 F | 20 F | 10 F | 10 F | 10 F | 5 F |
| 60^2^ | 5 F | 10 F | 10 F | 20 F | 10 F | 10 F | 10 F | 5 F |
| 170^2^ | 5 F | 10 F | 10 F | 20 F | 10 F | 10 F | 10 F | 5 F |
| 520^2^ | 5 F | 10 F | 10 F | 20 F | 10 F | 10 F | 10 F | 5 F |

SDD, sodium dichromate dihydrate; F, female mice

^1^this dose was not included in the F344 rat studies

^2^doses very similar to those used in the NTP 2-year cancer bioassay

^3^three biological replicates were processed for microarray analysis

**Supplementary Table II.** **Significant differentially expressed probes (DEPs) representing differentially expressed genes (DEGs) associated with Cr(VI) exposure in the oral mucosa of the rats and mice.** (**A**) 1 significant DEP representing 0 annotated genes in rats after 7 days of exposure, (**B**) 0 significant DEPs in rats 90 days of exposure, (**C**) 16 significant DEPs representing 14 DEGs in mice after 7 days of exposure, and (**D**) 1 significant DEP representing 1 DEG in mice after 90 days of exposure. Significant DEPs were defined as those with ANOVA p < 0.05, FDR q < 0.05, and fold change ≥ ± 2 (exposed versus unexposed). Fold change values (average exposed / averaged unexposed) are listed beneath each applicable Cr(VI) exposure concentration when a potential DEP met the above statistical criteria.

| **Probeset ID** | **RefSeq Accession** | **Entrez Gene ID** | **Gene Symbol** | **0.1 ppm** | **1.4 ppm** | **5 ppm^1^** | **20 ppm** | **60 ppm** | **180 ppm** |
| --- | --- | --- | --- | --- | --- | --- | --- | --- | --- |
| **(A) Rat Palate Day 8** | | | | | | | | | |
| A_44_P110221 | XM_006227158 |  |  |  |  |  | -2.15 |  |  |
| **(B) Rat Palate Day 91** | | | | | | | | | |
| none |  |  |  |  |  |  |  |  |  |
| **(C) Mouse Palate Day 8** | | | | | | | | | |
| A_51_P138378 | NM_008036 | 14282 | *Fosb* |  |  | 4.39 |  |  |  |
| A_51_P158538 | NM_011756 | 22695 | *Zfp36* |  |  | 2.06 |  |  |  |
| A_51_P181565 | NM_010415 | 15200 | *Hbegf* |  |  | 2.06 |  |  |  |
| A_51_P195958 | NM_009344 | 21664 | *Phlda1* |  |  | 2.27 |  |  |  |
| A_51_P239654 | NM_010444 | 15370 | *Nr4a1* |  |  | 5.25 |  |  |  |
| A_51_P367866 | NM_007913 | 13653 | *Egr1^2^* |  |  | 7.15 |  |  |  |
| A_51_P430900 | NM_013642 | 19252 | *Dusp1* |  |  | 2.31 |  |  |  |
| A_51_P462428 | XM_006519699 |  |  |  |  | 2.46 |  |  |  |
| A_51_P503494 | NM_018790 | 11838 | *Arc* |  |  | 2.48 |  |  |  |
| A_52_P262219 | NM_010234 | 14281 | *Fos^2^* |  |  | 10.26 |  |  |  |
| A_52_P452689 | NM_007498 | 11910 | *Atf3^2^* |  |  | 4.85 |  |  |  |
| A_52_P494622 | NM_013613 | 18227 | *Nr4a2* |  |  | 2.12 |  |  |  |
| A_52_P598732 |  |  |  |  |  | 2.08 |  |  |  |
| A_52_P82741 | NM_010479 | 193740 | *Hspa1a* |  |  | 2.75 |  |  |  |
| A_52_P971150 | XM_011239233 | 105244150 | *LOC105244150* |  |  | 2.02 |  |  |  |
| A_51_P346938 | NM_029796 | 76905 | *Lrg1* |  |  |  | 2.01 |  |  |
| **(D) Mouse Palate Day 91** | | | | | | | | | |
| A_51_P223776 | NM_145434 | 217166 | *Nr1d1* |  |  |  |  | 2.72 |  |

^1^ 5 ppm Cr(VI) was not evaluated in rats

^2^ Also identified with significant differential expression associated with 5 ppm Cr(VI) exposure using NCBI’s GEO2R online tool

**Supplementary Table III. Potential differentially expressed probes (pDEPs) representing potential differentially expressed genes (pDEGs) associated with Cr(VI) exposure in the oral mucosa of the rats and mice.** (**A**) 450 pDEPs representing 288 pDEGs in rats after 7 days of exposure, (**B**) 332 pDEPs representing 168 pDEGs in rats after 90 days of exposure, (**C**) 557 pDEPs representing 446 pDEGs in mice after 7 days of exposure, and (**D**) 345 pDEPs representing 294 pDEGs in mice after 90 days of exposure. pDEPs were defined as those with ANOVA p < 0.05 and fold change ≥ ± 1.5 (exposed versus unexposed). Fold change values (average exposed / averaged unexposed) are listed beneath each applicable Cr(VI) exposure concentration when a pDEP met the above statistical criteria.

| **Probeset ID** | **RefSeq Accession** | **Entrez Gene ID** | **Gene Symbol** | **0.1 ppm** | **1.4 ppm** | **5 ppm^1^** | **20 ppm** | **60 ppm** | **180 ppm** |
| --- | --- | --- | --- | --- | --- | --- | --- | --- | --- |
| **(A) Rat Palate Day 8** | | | | | | | | | |
| A_42_P472590 | NM_001108462 | 361472 | *Mthfd1l* |  |  |  |  | -1.78 |  |
| A_42_P473398 | NM_030845 | 81503 | *Cxcl1* |  |  |  |  |  | 1.90 |
| A_42_P484738 | NM_022266 | 64032 | *Ctgf* | -1.79 | -1.71 |  | -1.61 |  |  |
| A_42_P485961 | NM_012526 | 24259 | *Chgb* |  | 2.30 |  |  |  |  |
| A_42_P491119 | NM_012975 | 25474 | *Lgals4* |  | 4.65 |  |  |  |  |
| A_42_P496725 | NM_031119 | 81783 | *Ssb* |  | -1.71 |  |  |  |  |
| A_42_P513911 | XM_217612 |  |  |  | -1.58 |  |  |  |  |
| A_42_P524707 | NM_001024762 | 301323 | *Prim2* |  | 1.59 |  |  |  |  |
| A_42_P547246 | NM_030865 | 81523 | *Myoc* |  |  |  | 1.55 |  |  |
| A_42_P568943 | NM_001106825 | 300751 | *Rec114* |  |  |  |  |  | 1.67 |
| A_42_P585995 | XM_006231982 | 310376 | *Nim1k* |  |  |  | 1.54 |  |  |
| A_42_P589190 | NM_023104 | 65984 | *Aacs* |  |  |  |  | 1.73 |  |
| A_42_P597242 | NM_201560 | 296346 | *Oser1* | -1.58 |  |  |  |  |  |
| A_42_P648652 | NM_201419 | 362053 | *Clca4* |  | 4.62 |  |  |  |  |
| A_42_P669907 | NM_017302 | 29735 | *Slc16a7* |  | 1.50 |  |  |  |  |
| A_42_P677628 | NM_031642 | 58954 | *Klf6* | -1.57 |  |  |  |  |  |
| A_42_P707872 | NM_019362 | 54348 | *Stk39* |  | 1.50 |  |  |  |  |
| A_42_P708414 | NM_001191579 | 315670 | *Elmod1* |  | -1.65 |  |  |  |  |
| A_42_P708593 | NM_031678 | 63840 | *Per2* |  |  |  | -2.07 | -2.03 | -2.50 |
| A_42_P712801 | NM_178106 | 316077 | *Entpd3* | 1.50 |  |  |  |  |  |
| A_42_P735218 | XM_008768404 |  |  |  | -1.75 |  |  |  |  |
| A_42_P781659 | NM_001106264 | 292944 | *Reps1* |  | 1.68 |  |  |  |  |
| A_42_P786011 | NM_145674 | 246766 | *Ggta1* |  |  |  | 1.60 |  |  |
| A_42_P788302 | NM_001002803 | 407781 | *Btnl3* |  | 2.23 |  |  |  |  |
| A_42_P791512 | NM_013143 | 25684 | *Mep1a* |  | 2.70 |  |  |  |  |
| A_42_P797295 | NM_001034927 | 311642 | *Sulf2* |  | 1.56 |  |  |  |  |
| A_42_P808887 |  |  |  |  | 1.66 |  |  |  |  |
| A_42_P810796 | NM_001108819 | 363490 | *RGD1561958* |  | 2.42 |  |  |  |  |
| A_42_P813325 | XM_006257157 | 103690008 | *LOC103690008* |  | -1.98 |  |  |  |  |
| A_42_P816427 | NM_031716 | 65154 | *Wisp1* |  |  |  |  |  | 1.53 |
| A_42_P828757 | NM_053785 | 116467 | *Tm4sf4* |  | 2.11 |  |  |  |  |
| A_43_P10011 | NM_001025274 | 297942 | *Pnisr* |  | -1.68 |  |  |  |  |
| A_43_P10641 | NM_001106430 | 295037 | *Mgst2* |  | 1.84 |  |  |  |  |
| A_43_P10651 |  |  |  |  |  |  |  | 1.50 |  |
| A_43_P10854 | NM_001011934 | 293408 | *Hbs1l* |  | 1.50 |  |  |  |  |
| A_43_P11086 | NM_001024253 | 303924 | *Upk1b* | 1.96 |  |  |  |  |  |
| A_43_P11441 | NM_012510 | 24217 | *Atp4b* |  |  |  |  |  | -1.58 |
| A_43_P11444 | NM_012521 | 24249 | *S100g* |  | 45.94 |  |  |  |  |
| A_43_P11527 | NM_012715 | 25026 | *Adm* |  |  |  |  |  | 1.51 |
| A_43_P11661 | NM_013018 | 25531 | *Rab3a* |  |  |  | 1.65 |  |  |
| A_43_P11691 | NM_013068 | 25598 | *Fabp2* |  | 21.43 |  |  |  |  |
| A_43_P11723 | NM_013118 | 25656 | *Guca2a* |  | 4.28 |  |  |  |  |
| A_43_P11763 |  | 24170 | *Add1* | -1.65 |  |  |  |  |  |
| A_43_P12137 | NM_021858 | 60449 | *Gnb3* |  | -1.54 |  |  |  |  |
| A_43_P12348 | NM_022946 | 65040 | *Dlgap1* |  | 1.61 |  |  |  |  |
| A_43_P12403 | NM_024145 | 79113 | *Fgr* |  |  |  |  | 1.80 |  |
| A_43_P12843 | NM_053539 | 89784 | *Idi1* |  | 1.76 |  |  |  |  |
| A_43_P12873 | NM_053615 | 113927 | *Csnk1a1* |  | -1.81 |  |  |  |  |
| A_43_P12996 | NM_053955 | 117024 | *Crym* | 1.89 |  |  |  |  |  |
| A_43_P13004 | NM_053977 | 117048 | *Cdh17* |  | 10.19 |  |  |  |  |
| A_43_P13056 | NM_057193 | 117539 | *Il10ra* |  |  |  |  |  | 2.13 |
| A_43_P13424 | NM_173290 | 24919 | *Yy1* | -1.90 |  |  |  |  |  |
| A_43_P13564 | NM_001108589 | 362197 | *Mapkbp1* |  |  |  | 1.62 |  |  |
| A_43_P14040 | NM_134463 | 171497 | *Sgk2* |  | 1.66 |  |  |  |  |
| A_43_P14665 | NM_013097 | 25633 | *Dnase1* |  | 1.62 |  |  |  |  |
| A_43_P14683 | NM_001024790 | 360471 | *Usp7* |  | -2.00 |  |  |  |  |
| A_43_P15057 | NM_147210 | 259241 | *Nr1d2* |  |  |  |  | 1.60 |  |
| A_43_P15447 | NM_013414 | 25295 | *Bglap* |  |  |  |  |  | 2.04 |
| A_43_P15521 | NM_012556 | 24360 | *Fabp1* |  | 58.66 |  |  |  |  |
| A_43_P15781 | NM_053466 | 84598 | *Jak1* |  | -1.50 |  |  |  |  |
| A_43_P16045 |  |  |  | -1.60 |  |  |  |  |  |
| A_43_P16962 |  | 301128 | *Kdm4b* |  |  |  |  |  | -1.52 |
| A_43_P17374 | NM_001115032 | 679462 | *Tspan15* |  |  |  |  | 1.52 |  |
| A_43_P17580 | NM_001108377 | 361042 | *Pck2* |  | 1.54 |  |  |  |  |
| A_43_P17743 | NM_001009474 | 363465 | *Pir* |  | -1.85 |  |  |  |  |
| A_43_P17761 | NM_001109094 | 498420 | *Etaa1* |  | -2.06 |  |  |  |  |
| A_43_P17990 | NM_001012115 | 314638 | *Creb3l3* |  | 3.26 |  |  |  |  |
| A_43_P18207 | XM_003752777 |  |  |  |  |  |  |  | -1.51 |
| A_43_P18601 | XM_219296 |  |  |  | -1.93 |  |  |  |  |
| A_43_P18842 | NM_001007797 | 309945 | *Arrdc3* |  | -1.68 |  |  |  |  |
| A_43_P19143 | NM_001030023 | 287398 | *Map2k4* |  | 1.58 |  |  |  |  |
| A_43_P19157 | XM_006243553 |  |  |  |  |  | 1.52 |  |  |
| A_43_P19527 | NM_001037205 | 360997 | *Abcg3l4* |  | -1.50 |  |  |  |  |
| A_43_P19625 | NM_001105937 | 288743 | *Sgsm1* |  | 1.82 |  |  |  |  |
| A_43_P19657 | NM_001107903 | 312946 | *Tmem68* |  |  |  | -1.60 |  |  |
| A_43_P19791 | XM_003754174 | 100359558 | *Fam177a1* |  | -1.80 |  |  |  |  |
| A_43_P19970 | NM_001108281 | 360563 | *RGD1308139* |  | 1.52 |  |  |  |  |
| A_43_P20191 | NM_001108772 | 363109 | *Pgm3* |  | 1.74 |  |  |  |  |
| A_43_P20534 | NM_001037770 | 300724 | *Fbxo22* |  | 1.60 |  |  |  |  |
| A_43_P20539 | NM_001012142 | 360612 | *Scrn2* |  | 1.53 |  |  |  |  |
| A_43_P20751 | NM_001107445 | 307997 | *Hspa12a* |  |  |  | 1.54 |  |  |
| A_43_P20861 | NM_001127299 | 313969 | *Fam84a* |  |  |  | 1.70 |  |  |
| A_43_P20949 | NM_001025682 | 308958 | *Cdr2* |  | 1.66 |  |  |  |  |
| A_43_P21146 | NM_001106079 | 290673 | *Atp13a1* | 1.52 |  |  |  |  |  |
| A_43_P21191 | XM_008765291 |  |  |  | 1.58 |  |  |  |  |
| A_43_P21256 | NM_001083966 | 309812 | *Rev3l* |  | 1.76 |  |  |  |  |
| A_43_P21813 | XM_008774981 |  |  |  |  |  |  |  | 1.79 |
| A_43_P21836 | XM_008758643 |  |  |  |  |  |  | -1.52 |  |
| A_43_P22909 | XM_008760977 |  |  |  |  |  |  | -1.54 |  |
| A_43_P23023 | NM_001126287 | 361790 | *Ppp1r18* |  | 1.62 |  |  |  |  |
| A_43_P23101 |  |  |  | 2.03 |  |  |  |  |  |
| A_43_P23215 | NM_001025737 | 362619 | *Smpdl3b* |  | 1.68 |  |  |  |  |
| A_44_P100009 | NM_001107393 | 307483 | *Diaph1* |  | 1.53 |  |  |  |  |
| A_44_P1002531 |  |  |  |  |  |  | -2.12 |  |  |
| A_44_P1003716 | NM_001082409 | 361219 | *Fbxw17* |  | 1.54 |  |  |  |  |
| A_44_P1005450 | XM_008768057 |  |  |  | -2.08 |  |  |  |  |
| A_44_P100609 | NM_001290133 | 686103 | *Frg1* |  | -1.53 |  |  |  |  |
| A_44_P1009594 | NM_001024232 | 170509 | *Cyp3a62* |  | 4.07 |  |  |  |  |
| A_44_P1022403 | NM_001004204 | 288616 | *Tmem248* |  | 1.54 |  |  |  |  |
| A_44_P1024721 | NM_031664 | 60423 | *Slc28a2* |  | 2.24 |  |  |  |  |
| A_44_P1028477 | NM_001107196 | 304962 | *Atf6* |  | -1.67 |  |  |  |  |
| A_44_P1030225 | NM_012737 | 25080 | *Apoa4* |  | 8.63 |  |  |  |  |
| A_44_P1034533 | XM_003754543 |  |  |  | -2.80 |  |  |  |  |
| A_44_P1034637 | NM_001012068 | 309070 | *Uros* |  | -1.69 |  |  |  |  |
| A_44_P1036481 | NR_111961 | 360865 | *Prrc2c* |  | -1.65 |  |  |  |  |
| A_44_P1037526 | NM_001047087 | 292148 | *Eif3a* |  | -1.55 |  |  |  |  |
| A_44_P1037706 | NM_001106957 | 302554 | *Glod5* |  | 2.69 |  |  |  |  |
| A_44_P1037806 | NM_053318 | 58917 | *Hpx* |  | 2.38 |  |  |  |  |
| A_44_P1046205 | NM_001106663 | 298144 | *Kdm4c* |  | -1.59 |  |  |  |  |
| A_44_P1046615 | XM_008760848 | 687030 | *Muc3* |  | 2.09 |  |  |  |  |
| A_44_P1049015 |  |  |  |  | -1.53 |  |  |  |  |
| A_44_P1054213 | NM_013083 | 25617 | *Hspa5* |  |  |  | -1.64 |  |  |
| A_44_P105634 | NM_001007658 | 300015 | *Them6* |  |  |  |  |  | 1.52 |
| A_44_P1058179 | XM_006244452 | 316219 | *Trerf1* |  | -1.77 |  |  |  |  |
| A_44_P1059122 | NM_001025683 | 309145 | *RGD1311946* |  | 1.50 |  |  |  |  |
| A_44_P1059463 | NM_001037653 | 654496 | *Fubp1* |  | -1.78 |  |  |  |  |
| A_44_P1060096 | NM_001108869 | 364388 | *Cideb* |  | 1.95 |  |  |  |  |
| A_44_P108217 | XM_003754311 | 680860 | *Eppk1* |  |  |  |  | -1.63 |  |
| A_44_P109977 | NM_001012210 | 364398 | *Trim13* | 1.51 |  |  |  |  |  |
| A_44_P110221 | XM_006227158 |  |  |  |  |  | -2.15 |  |  |
| A_44_P113643 |  |  |  |  | -1.52 |  |  |  |  |
| A_44_P116176 | XM_006243775 |  |  |  |  |  | -1.72 |  |  |
| A_44_P117684 |  |  |  |  |  |  |  |  | 1.78 |
| A_44_P118734 | NM_001001024 | 405909 | *Olr95* | 1.61 |  |  |  |  |  |
| A_44_P119160 | NM_001106375 | 294103 | *Papss2* |  | 1.85 |  |  |  |  |
| A_44_P119682 | XM_003750241 |  |  |  | 1.67 |  |  |  |  |
| A_44_P123537 | NM_001271140 | 116694 | *Capn9* | 1.60 |  |  |  |  |  |
| A_44_P124717 | NM_001014015 | 307956 | *Rbm34* |  | 1.51 |  |  |  |  |
| A_44_P126938 | NM_133622 | 171161 | *Csap1* | 4.96 |  |  |  |  |  |
| A_44_P128186 | NM_053305 | 29407 | *Tas1r1* |  | -1.64 |  |  |  |  |
| A_44_P132346 |  |  |  |  | -1.53 |  |  |  |  |
| A_44_P135413 |  |  |  |  |  |  | -1.51 |  |  |
| A_44_P136161 |  |  |  |  | -1.73 |  |  |  |  |
| A_44_P138167 | NM_001191930 | 498272 | *Uap1* |  | 1.72 |  |  |  |  |
| A_44_P143886 | NM_022392 | 64194 | *Insig1* |  | 1.75 |  |  |  |  |
| A_44_P146448 |  |  |  |  |  |  |  | 1.68 |  |
| A_44_P149370 | NM_198733 | 292728 | *Cyp2b21* | 1.75 |  |  |  |  |  |
| A_44_P149993 | NM_001033680 | 25716 | *Syt1* |  |  |  |  |  | 1.61 |
| A_44_P158441 | NM_013131 | 25672 | *Nr3c2* |  |  |  | -1.91 |  |  |
| A_44_P159218 | NM_001107770 | 311401 | *Sppl2a* |  | 1.90 |  |  |  |  |
| A_44_P159430 | NM_001127575 | 691455 | *Calml4* |  | 3.08 |  |  |  |  |
| A_44_P161052 | NM_001040019 | 501072 | *Acaa1b* |  | 1.59 |  |  |  |  |
| A_44_P161470 | NM_001033076 | 613223 | *Defa6* |  | 2.59 |  |  |  |  |
| A_44_P165421 |  |  |  |  |  |  |  | -1.58 |  |
| A_44_P166046 | NM_001039031 | 361730 | *Tkfc* |  | 1.57 |  |  |  |  |
| A_44_P166206 | NM_001024260 | 311111 | *Nostrin* |  | 1.74 |  |  |  |  |
| A_44_P173698 |  |  |  |  | -1.64 |  |  |  |  |
| A_44_P175041 | NM_145775 | 252917 | *Nr1d1* |  |  |  | 1.69 | 1.66 | 1.84 |
| A_44_P178616 |  |  |  |  | -1.83 |  |  |  |  |
| A_44_P178770 |  |  |  |  | -1.51 |  |  |  |  |
| A_44_P183488 | NM_022260 | 64026 | *Casp7* |  | 1.52 |  |  |  |  |
| A_44_P184273 | NM_012504 | 24211 | *Atp1a1* |  | 1.53 |  |  |  |  |
| A_44_P184553 | NM_148889 | 259271 | *Aptx* | -1.52 |  |  |  |  |  |
| A_44_P187293 |  |  |  |  | 1.57 |  |  |  |  |
| A_44_P189506 |  |  |  |  |  |  |  |  | 1.67 |
| A_44_P190855 |  |  |  |  |  |  | 1.54 |  |  |
| A_44_P192964 | NM_001013994 | 305811 | *Rcor2* |  |  |  |  |  | 1.71 |
| A_44_P194230 | NM_022632 | 360272 | *Slit2* | -1.56 |  |  |  |  |  |
| A_44_P195468 | NM_133423 | 170956 | *Ythdc1* |  | -2.01 |  |  |  |  |
| A_44_P196034 |  |  |  |  | -1.64 |  |  |  |  |
| A_44_P196933 | NM_001014031 | 309656 | *Cmtr1* |  | 1.57 |  |  |  |  |
| A_44_P201028 | NM_053727 | 114519 | *Nfil3* |  |  |  |  | -1.51 |  |
| A_44_P201102 |  | 64621 | *Akp3* |  | 7.28 |  |  |  |  |
| A_44_P201499 |  |  |  |  |  |  | 1.53 |  |  |
| A_44_P202183 |  |  |  | -1.62 |  |  |  |  |  |
| A_44_P205523 | XR_593925 | 103693098 | *LOC103693098* |  |  |  |  | 1.77 |  |
| A_44_P205771 | NM_053880 | 116659 | *Dync1i2* |  | -1.55 |  |  |  |  |
| A_44_P205869 | NM_138891 | 192251 | *Gpr149* |  | 2.16 |  |  |  |  |
| A_44_P208021 | NM_001000773 | 405048 | *Olr1452* | 1.74 |  |  |  |  |  |
| A_44_P210240 | NM_012998 | 25506 | *P4hb* |  | 1.72 |  |  |  |  |
| A_44_P211630 |  |  |  |  |  |  |  |  | 1.72 |
| A_44_P220827 | NM_001012051 | 306695 | *Zfp367* |  |  |  |  |  | -1.52 |
| A_44_P222761 | XM_008763844 | 502957 | *RGD1560884* |  |  |  | -1.74 |  |  |
| A_44_P223766 |  |  |  | -1.53 |  |  |  |  |  |
| A_44_P224853 | XM_008760473 |  |  |  | -1.52 |  |  |  |  |
| A_44_P226162 |  |  |  |  | 1.71 |  |  |  |  |
| A_44_P229887 | NM_001012013 | 303577 | *Acbd4* | -1.57 |  |  |  |  |  |
| A_44_P231180 |  |  |  |  | 1.55 |  |  |  |  |
| A_44_P231994 | XM_008762849 |  |  |  | 1.51 |  |  |  |  |
| A_44_P234624 | NM_001107061 | 303614 | *Smurf2* |  | -1.80 |  |  |  |  |
| A_44_P234675 |  |  |  |  | -1.60 |  |  |  |  |
| A_44_P235165 | NM_001012148 | 360823 | *Ankrd13a* | -1.58 |  |  |  |  |  |
| A_44_P236350 |  |  |  |  | -1.65 |  |  |  |  |
| A_44_P239849 |  |  |  |  |  |  | -1.52 |  |  |
| A_44_P242588 |  |  |  |  |  |  |  | -1.51 |  |
| A_44_P243292 | NM_022665 | 24197 | *Alpi* |  | 6.12 |  |  |  |  |
| A_44_P243439 |  |  |  |  |  |  | 1.69 |  |  |
| A_44_P247108 |  |  |  |  | -1.64 |  |  |  |  |
| A_44_P252435 | NM_201416 | 292067 | *Zc3h18* |  | -1.57 |  |  |  |  |
| A_44_P256102 |  |  |  |  | -1.56 |  |  |  |  |
| A_44_P256188 | NM_001195471 | 497860 | *Rpl39l* |  | 2.12 |  |  |  |  |
| A_44_P257378 | NM_001000635 | 404828 | *Olr660* |  | -1.82 |  |  |  |  |
| A_44_P260433 | NM_001163273 | 292051 | *Cmip* |  | -1.52 |  |  |  |  |
| A_44_P260709 |  |  |  | 1.71 |  |  |  |  |  |
| A_44_P261259 |  |  |  |  | -1.76 |  |  |  |  |
| A_44_P263635 | NM_017243 | 29562 | *Prps1* |  | 1.70 |  |  |  |  |
| A_44_P267799 | NM_001107107 | 304063 | *Ets2* |  | 1.62 |  |  |  |  |
| A_44_P269690 | NM_001107691 | 310641 | *Trim46* |  |  |  |  |  | -1.66 |
| A_44_P270843 |  |  |  |  | -1.71 |  |  |  |  |
| A_44_P271658 | NM_053289 | 24618 | *Reg3b* |  | 8.06 |  |  |  |  |
| A_44_P272773 |  |  |  | 2.57 |  |  |  |  |  |
| A_44_P274036 | NM_057202 | 117550 | *Kif5b* |  | -1.53 |  |  |  |  |
| A_44_P274976 | NM_001191778 | 299699 | *Aldh1l2* |  | 1.69 |  |  |  |  |
| A_44_P277851 |  |  |  |  |  |  | -1.51 |  |  |
| A_44_P280062 |  |  |  |  |  |  | -1.59 |  |  |
| A_44_P284200 | NM_001014058 | 312688 | *Usp18* |  | 1.66 |  |  |  |  |
| A_44_P286008 | NM_001109562 | 689984 | *Gpr137* |  | -2.06 |  |  |  |  |
| A_44_P286364 |  |  |  |  | 1.58 |  |  |  |  |
| A_44_P287250 | NM_022521 | 64313 | *Oat* |  | 1.51 |  |  |  |  |
| A_44_P288241 | XM_001059692 |  |  |  | -1.85 |  |  | -1.55 |  |
| A_44_P291175 | XM_008767423 | 301701 | *Ndc80* |  | 1.62 |  |  |  |  |
| A_44_P299870 | NM_175761 | 299331 | *Hsp90aa1* |  | -1.84 |  |  |  |  |
| A_44_P304323 | NM_031116 | 81780 | *Ccl5* |  | 1.51 |  |  |  |  |
| A_44_P309160 | NM_001013984 | 304860 | *Npl* | -1.51 |  |  |  |  |  |
| A_44_P310714 |  |  |  |  | -1.80 |  |  |  |  |
| A_44_P311324 | NM_001198676 | 361523 | *Cyp2b2* | 1.78 |  |  |  |  |  |
| A_44_P311762 | NM_001015032 | 366061 | *Galnt3* |  | 1.56 |  |  |  |  |
| A_44_P312089 | NM_001014100 | 316326 | *Neurl3* |  |  |  |  | 1.54 |  |
| A_44_P312745 | NM_001108006 | 313825 | *Vps13d* |  |  |  |  |  | 1.61 |
| A_44_P316194 | NM_017156 | 29295 | *Cyp2b12* | 1.81 |  |  |  |  |  |
| A_44_P319035 |  |  |  |  | 1.76 |  |  |  |  |
| A_44_P321675 | NM_001107058 | 303604 | *Map3k3* |  | 1.54 |  |  |  |  |
| A_44_P321735 |  |  |  |  |  |  | -1.52 |  |  |
| A_44_P323304 | NM_013188 | 25739 | *Pygb* |  | 1.66 |  |  |  |  |
| A_44_P324093 | NM_001191790 | 315327 | *Spryd3* |  |  |  | 1.57 |  |  |
| A_44_P328338 | NM_001025683 | 309145 | *RGD1311946* |  | 1.65 |  |  |  |  |
| A_44_P329090 | NM_001015026 | 362326 | *Tspan12* |  | 1.52 |  |  |  |  |
| A_44_P330318 | NM_001012035 | 305242 | *Cdkl2* |  |  |  | -1.63 |  |  |
| A_44_P330555 | XM_008769016 |  |  |  |  |  | 1.54 |  |  |
| A_44_P330778 | XM_008771602 | 306889 | *RGD1564786* |  |  |  |  |  | 1.64 |
| A_44_P332971 |  |  |  | -1.57 |  |  |  |  |  |
| A_44_P335898 | NM_001008863 | 408223 | *Usp54* |  | 1.59 |  |  |  |  |
| A_44_P338184 |  |  |  |  | -1.69 |  |  |  |  |
| A_44_P342721 |  |  |  |  | -1.82 |  |  |  |  |
| A_44_P344444 | NM_022589 | 64521 | *Tspan2* |  | -1.64 |  |  |  |  |
| A_44_P345207 |  |  |  |  | 1.62 |  |  |  |  |
| A_44_P346572 | NM_001008891 | 361233 | *Ssr1* |  | 1.53 |  |  |  |  |
| A_44_P347431 |  |  |  |  |  |  | -1.96 |  |  |
| A_44_P348418 | XM_008771429 | 290963 | *Npepo* |  | -1.85 |  |  |  |  |
| A_44_P354911 | NM_001108416 | 361256 | *Svil* |  | -1.82 |  |  |  |  |
| A_44_P358828 | XM_006254627 |  |  |  | 1.55 |  |  |  |  |
| A_44_P360486 | NM_016999 | 24307 | *Cyp4b1* | -1.63 |  |  |  |  |  |
| A_44_P361416 | XM_008766354 | 315652 | *Colca2* |  |  |  |  |  | 1.73 |
| A_44_P362992 | NM_013176 | 25720 | *Tcf12* |  | -1.58 |  |  |  |  |
| A_44_P366192 |  |  |  |  | 1.72 |  |  |  |  |
| A_44_P366979 |  |  |  |  | -1.51 |  |  |  |  |
| A_44_P368249 | NM_001173555 | 362508 | *Spag8* | 1.52 |  |  |  |  |  |
| A_44_P371050 |  |  |  |  |  |  |  |  | 1.52 |
| A_44_P371125 | NM_022513 | 64305 | *Sult1b1* |  | 1.59 |  |  |  |  |
| A_44_P371984 |  | 25253 | *Dpp4* |  | 3.36 |  |  |  |  |
| A_44_P372439 | NM_017041 | 24674 | *Ppp3ca* |  | -1.64 |  |  |  |  |
| A_44_P372601 | XM_001059157 |  |  |  | -1.50 |  |  |  |  |
| A_44_P372602 |  |  |  |  | -1.53 |  |  |  |  |
| A_44_P373389 | XM_008769295 |  |  |  |  |  |  | -1.50 |  |
| A_44_P376449 |  |  |  |  | -1.54 |  |  |  |  |
| A_44_P377670 | NM_001107917 | 313087 | *Cpne3* |  | -1.54 |  |  |  |  |
| A_44_P377835 | NM_001105714 | 24881 | *Wnt1* |  | -1.76 |  |  |  |  |
| A_44_P378362 | NM_001109404 | 680419 | *Rsu1* |  | 1.56 |  |  |  |  |
| A_44_P379412 | NM_212489 | 406160 | *Btnl8* |  | 1.54 |  |  |  |  |
| A_44_P380243 |  |  |  |  | -1.51 |  |  |  |  |
| A_44_P380727 | XM_006246054 |  |  |  | -1.70 |  |  |  |  |
| A_44_P382593 |  | 303313 | *Scarf1* |  | 1.64 |  |  |  |  |
| A_44_P383231 |  |  |  |  | 1.67 |  |  |  |  |
| A_44_P387017 |  |  |  |  |  |  | -1.63 |  |  |
| A_44_P390062 |  |  |  |  |  |  |  |  | 1.55 |
| A_44_P390873 |  |  |  |  | -1.51 |  |  |  |  |
| A_44_P393706 | XM_008770176 |  |  |  | -1.83 |  |  |  |  |
| A_44_P397424 |  |  |  |  | -1.50 |  |  |  |  |
| A_44_P397836 | NM_001002025 | 432367 | *Npc1l1* |  | 3.31 |  |  |  |  |
| A_44_P398388 | XM_008768802 |  |  | 1.67 |  |  |  |  |  |
| A_44_P402318 |  |  |  | -1.55 |  |  |  |  |  |
| A_44_P402956 | NM_001077645 | 293508 | *Prkacb* |  |  |  | -1.73 |  |  |
| A_44_P403501 | NM_001127597 | 690043 | *Rnf168* |  | -1.62 |  |  |  |  |
| A_44_P403532 | XM_008761788 |  |  |  | 1.64 |  |  |  |  |
| A_44_P404367 |  |  |  |  | 1.51 |  |  |  |  |
| A_44_P405177 | NM_012553 | 24332 | *Cela2a* |  |  |  | -1.58 |  |  |
| A_44_P406074 | NM_022392 | 64194 | *Insig1* |  | 1.59 |  |  |  |  |
| A_44_P414582 | NM_133603 | 171138 | *Kcne2* |  |  |  | 1.64 |  |  |
| A_44_P416824 | NM_001100566 | 305858 | *Otx2* | 1.50 |  |  |  |  |  |
| A_44_P417031 | NM_001108490 | 361600 | *Tm6sf1* |  | 1.58 |  |  |  |  |
| A_44_P417658 | NM_138870 | 85423 | *Tfpt* |  | -1.59 |  |  |  |  |
| A_44_P419162 | NM_013130 | 25671 | *Smad1* |  | 1.53 |  |  |  |  |
| A_44_P421547 | NM_053295 | 25403 | *Cast* |  | -1.92 |  |  |  |  |
| A_44_P422346 | NM_001100502 | 288333 | *Gbe1* |  | 1.66 |  |  |  |  |
| A_44_P425027 |  |  |  |  | -1.56 |  |  |  |  |
| A_44_P425150 | NM_134418 | 171459 | *Gp2* |  |  |  |  |  | -1.50 |
| A_44_P426578 |  |  |  | -1.52 |  |  |  |  |  |
| A_44_P427596 | NM_012556 | 24360 | *Fabp1* |  | 34.94 |  |  |  |  |
| A_44_P428326 | NM_173094 | 24450 | *Hmgcs2* |  |  |  | 1.83 |  |  |
| A_44_P429476 | NM_001107105 | 304056 | *Zfp295* |  |  |  |  | 1.65 |  |
| A_44_P430027 |  |  |  |  | 1.69 |  |  |  |  |
| A_44_P430437 |  |  |  |  |  |  | -1.77 |  |  |
| A_44_P436191 | XM_214434 |  |  |  | 2.86 |  |  |  |  |
| A_44_P437346 |  |  |  |  |  |  | -1.57 |  |  |
| A_44_P438355 | NM_001109454 | 685076 | *Pgm2l1* |  |  |  | -1.53 |  |  |
| A_44_P439276 | NM_001305175 | 291031 | *Fam8a1* |  |  |  |  |  | 1.68 |
| A_44_P440556 | XM_008768788 |  |  |  | 5.25 |  |  |  |  |
| A_44_P440987 | NM_001108531 | 361832 | *Bicc1* |  | -1.65 |  |  |  |  |
| A_44_P441339 | NM_001013081 | 296621 | *Phyhd1* | 1.53 |  |  |  |  |  |
| A_44_P441881 |  |  |  |  | 1.52 |  |  |  |  |
| A_44_P446409 | NM_031018 | 81647 | *Atf2* |  |  |  | 1.56 |  |  |
| A_44_P448928 |  |  |  |  | -1.55 |  |  |  |  |
| A_44_P449522 | NM_001100698 | 310375 | *Zfp131* |  | -1.55 |  |  |  |  |
| A_44_P451357 | NM_012656 | 24791 | *Sparc* | -1.64 |  |  |  |  |  |
| A_44_P452546 | NM_001107725 | 310855 | *Gstcd* |  | 1.64 |  |  |  |  |
| A_44_P453975 |  |  |  |  | -1.88 |  |  |  |  |
| A_44_P454645 | NM_001047103 | 360882 | *Cadm3* | -1.66 |  |  |  |  |  |
| A_44_P459052 |  | 60423 | *Slc28a2* |  | 1.72 |  |  |  |  |
| A_44_P461187 | NM_203337 | 363040 | *St3gal4* |  | 1.90 |  |  |  |  |
| A_44_P463749 | NM_001270954 | 64455 | *Rasd1* |  | 1.55 |  |  |  |  |
| A_44_P466108 | NM_031566 | 29227 | *Nfib* |  | -1.60 |  |  |  |  |
| A_44_P466183 | NM_133410 | 170923 | *Rap2b* |  | -1.55 |  |  |  |  |
| A_44_P466331 | NM_001024752 | 293691 | *Snx15* |  | 1.60 |  |  |  |  |
| A_44_P468360 | NM_019340 | 54293 | *Rgs3* |  | 1.53 |  |  |  |  |
| A_44_P469531 | NM_031537 | 24906 | *LOC24906* |  |  |  | 1.62 |  |  |
| A_44_P471217 |  |  |  |  |  |  |  |  | -1.85 |
| A_44_P471853 | XM_003752989 |  |  |  | -1.64 |  |  |  |  |
| A_44_P472996 | NM_013132 | 25673 | *Anxa5* |  | 1.57 |  |  |  |  |
| A_44_P473389 | XM_008771913 | 291356 | *Etl4* |  | 1.58 |  |  |  |  |
| A_44_P474660 |  |  |  |  | -1.53 |  |  |  |  |
| A_44_P478939 |  |  |  | -1.62 |  |  |  |  |  |
| A_44_P480329 | NM_001047900 | 360811 | *Naa25* |  | -1.72 |  |  |  |  |
| A_44_P482753 |  |  |  |  | -2.01 |  |  |  |  |
| A_44_P486055 | NM_053459 | 84590 | *Rab27b* |  | -1.56 |  |  |  |  |
| A_44_P487000 | NM_001000651 | 404848 | *Olr623* |  |  |  | -1.60 |  |  |
| A_44_P487112 | NM_001012742 | 308937 | *Wee1* |  |  |  | -1.53 |  | -1.63 |
| A_44_P487240 | NM_017268 | 29637 | *Hmgcs1* |  | 2.11 |  |  |  |  |
| A_44_P488467 | NM_001108655 | 362484 | *Plekhf2* |  | -1.58 |  |  |  |  |
| A_44_P491187 |  |  |  |  | -1.77 |  |  |  |  |
| A_44_P491674 | NM_022852 | 29535 | *Pdx1* |  | 2.22 |  |  |  |  |
| A_44_P492214 | NM_001106463 | 295361 | *Eps8l3* |  | 3.87 |  |  |  |  |
| A_44_P494274 | NM_053686 | 114246 | *Trpv6* |  | 2.15 |  |  |  |  |
| A_44_P494608 | NM_001108444 | 361388 | *Gab1* |  |  |  | 1.58 |  |  |
| A_44_P495480 | NM_022197 | 314322 | *Fos* |  |  |  |  |  | 3.34 |
| A_44_P497262 | XM_006220668 |  |  |  |  |  |  | -1.51 |  |
| A_44_P500013 | NM_031012 | 81641 | *Anpep* |  | 1.57 |  |  |  |  |
| A_44_P500740 |  |  |  | -1.52 |  |  |  |  |  |
| A_44_P503004 |  |  |  |  | 1.64 |  |  |  |  |
| A_44_P506064 | NM_001191778 | 299699 | *Aldh1l2* |  | 1.55 |  |  |  |  |
| A_44_P508691 | NM_001127654 | 689029 | *Tm6sf2* |  | 2.04 |  |  |  |  |
| A_44_P511114 | NM_001170606 | 361319 | *Spink5* |  | -1.65 |  |  | -1.83 |  |
| A_44_P512828 | NM_053322 | 58958 | *Nup210* | 1.53 |  |  |  |  |  |
| A_44_P513877 |  |  |  |  | 1.68 |  |  |  |  |
| A_44_P516101 | NM_001025285 | 362957 | *Ankrd54* |  | 1.58 |  |  |  |  |
| A_44_P518528 | NM_001108276 | 360534 | *Trim11* |  | 1.62 |  |  |  |  |
| A_44_P520789 | XM_002726857 |  |  |  |  |  | 1.56 |  |  |
| A_44_P522915 |  |  |  |  |  |  |  |  | -1.50 |
| A_44_P523000 |  |  |  |  | 2.45 |  |  |  |  |
| A_44_P525203 | XM_008769328 |  |  |  | -1.54 |  |  |  |  |
| A_44_P525817 | NM_001100903 | 362844 | *Brd4* |  | -1.63 |  |  |  |  |
| A_44_P527680 | NM_001106639 | 297911 | *Otud6b* |  | 1.51 |  |  |  |  |
| A_44_P529418 | NM_001037093 | 246150 | *Akap9* |  | -1.62 |  |  |  |  |
| A_44_P529950 | XM_006222692 |  |  |  |  |  |  | 1.55 |  |
| A_44_P531245 |  |  |  |  |  |  |  |  | 1.71 |
| A_44_P531647 | NM_017168 | 29337 | *Plcg2* |  | 1.51 |  |  |  |  |
| A_44_P532548 | NM_001014120 | 360627 | *Fkbp10* |  | 1.85 |  |  |  |  |
| A_44_P536086 | NM_134409 | 171449 | *Zg16* |  | 4.18 |  |  |  |  |
| A_44_P537780 |  |  |  |  | -1.56 |  |  |  |  |
| A_44_P539349 | NM_001107015 | 303256 | *Tm4sf5* |  | 8.41 |  |  |  |  |
| A_44_P543965 |  |  |  |  | 1.59 |  |  |  |  |
| A_44_P545314 | NM_145678 | 246772 | *Vps4a* |  |  |  |  | -1.58 |  |
| A_44_P547168 | NM_022392 | 64194 | *Insig1* |  | 1.67 |  |  |  |  |
| A_44_P547191 |  |  |  | -1.53 |  |  |  |  |  |
| A_44_P548079 |  |  |  |  |  |  |  |  | -1.69 |
| A_44_P548303 | NM_001009688 | 309041 | *Thumpd1* |  | -1.66 |  |  |  |  |
| A_44_P548481 | NM_001108884 | 364814 | *Psma8* |  | -1.59 |  |  |  |  |
| A_44_P548726 | NM_001009664 | 299647 | *Zfp414* |  |  |  |  |  | 1.51 |
| A_44_P551872 |  |  |  | -1.64 |  |  |  |  |  |
| A_44_P551963 |  |  |  |  | -1.57 |  |  |  |  |
| A_44_P552692 | NM_019287 | 54225 | *Apob* |  | 8.96 |  |  |  |  |
| A_44_P554299 |  |  |  |  | 1.56 |  |  |  |  |
| A_44_P561191 | NR_126581 | 104845260 | *Lnc215* | -1.54 |  |  |  |  |  |
| A_44_P562314 | XM_006231066 | 59267 | *Syt7* |  |  |  |  | 1.51 |  |
| A_44_P562582 | NM_001107814 | 311783 | *Il36rn* | -1.61 |  |  |  |  |  |
| A_44_P568560 | XM_008767138 | 81646 | *Creb1* |  | -1.82 |  |  |  |  |
| A_44_P574106 |  |  |  |  | -1.89 |  |  |  |  |
| A_44_P578376 |  |  |  |  |  |  |  |  | 1.61 |
| A_44_P578428 |  |  |  |  | -1.57 |  |  |  |  |
| A_44_P578505 | XM_008774984 | 100364027 | *LOC100364027* |  | 3.86 |  |  |  |  |
| A_44_P579385 | NM_001191951 | 191572 | *Ahnak* |  | -2.12 |  |  |  |  |
| A_44_P579975 |  |  |  |  | -1.50 |  |  |  |  |
| A_44_P582756 |  |  |  |  | 1.63 |  |  |  |  |
| A_44_P589989 | XM_008771855 | 291318 | *Nmt2* |  | 1.55 |  |  |  |  |
| A_44_P590658 |  |  |  |  | -1.59 |  |  |  |  |
| A_44_P591698 |  |  |  |  | -1.64 |  |  |  |  |
| A_44_P592915 |  |  |  |  |  |  | -1.56 |  |  |
| A_44_P593668 | XM_008771429 | 290963 | *Npepo* |  |  |  |  |  | 2.07 |
| A_44_P594610 | NM_001025050 | 500545 | *Cdca8* |  |  |  |  |  | -1.56 |
| A_44_P603086 |  |  |  |  | -1.52 |  |  |  |  |
| A_44_P606251 | XM_006240348 | 500693 | *Ccdc176* |  |  |  |  |  | 1.57 |
| A_44_P607556 |  |  |  |  |  |  |  |  | 1.96 |
| A_44_P609306 | XM_008774059 | 498819 | *Greb1l* |  | 1.54 |  |  |  |  |
| A_44_P612314 |  |  |  |  | 2.64 |  |  |  |  |
| A_44_P613130 | XM_008762204 | 311348 | *Cdan1* |  |  |  |  |  | -1.53 |
| A_44_P623560 | XR_146000 | 313553 | *Rimkla* | 1.50 |  |  |  |  |  |
| A_44_P623737 |  |  |  | 1.57 |  |  |  |  |  |
| A_44_P636423 | XM_008774810 | 103695060 | *LOC103695060* | 2.53 |  |  |  |  |  |
| A_44_P639283 |  |  |  |  | 1.72 |  |  |  |  |
| A_44_P640131 | XM_002729786 | 688019 | *Thap2* |  | -1.77 |  |  |  |  |
| A_44_P640421 |  |  |  |  | 1.55 |  |  |  |  |
| A_44_P652617 | NM_022933 | 65027 | *Chd8* |  | -1.57 |  |  |  |  |
| A_44_P653165 |  |  |  |  | 2.19 |  |  |  |  |
| A_44_P653701 | NM_001106536 | 296344 | *Mybl2* |  |  |  |  |  | -1.53 |
| A_44_P663759 |  |  |  |  | -1.79 |  |  |  |  |
| A_44_P667896 | NM_001195564 | 315732 | *Uaca* |  | -1.63 |  |  |  |  |
| A_44_P671265 |  |  |  |  |  |  | 1.54 |  |  |
| A_44_P683862 | NM_133582 | 171113 | *Blcap* | -1.52 |  |  |  |  |  |
| A_44_P684922 | NM_001107001 | 303132 | *Aff4* |  | -1.60 |  |  |  |  |
| A_44_P685023 |  |  |  |  | -1.66 |  |  |  |  |
| A_44_P690298 |  |  |  | -1.69 |  |  |  |  |  |
| A_44_P699780 | XM_008771446 | 59328 | *Smad5* |  | -1.71 |  |  |  |  |
| A_44_P701038 |  |  |  |  | 1.66 |  |  |  |  |
| A_44_P730874 |  |  |  |  | -1.65 |  |  |  |  |
| A_44_P740845 |  |  |  | -1.53 |  |  |  |  |  |
| A_44_P744605 | NM_001047887 | 311013 | *Arsj* |  |  |  |  |  | 1.67 |
| A_44_P764517 | XM_008770481 | 117955 | *Slc4a7* |  | 1.58 |  |  |  |  |
| A_44_P778944 | XM_008776764 | 300807 | *Rora* |  | -1.54 |  |  |  |  |
| A_44_P780510 |  |  |  | 1.62 |  |  |  | 1.58 |  |
| A_44_P782882 |  |  |  | -1.75 |  |  |  |  |  |
| A_44_P792495 | XM_008773556 | 302492 | *Mbnl3* | -1.64 |  |  |  |  |  |
| A_44_P804460 |  |  |  |  |  |  |  | 1.61 |  |
| A_44_P807091 | NM_001106297 | 293505 | *Cd2bp2* |  | 2.00 |  |  |  |  |
| A_44_P819401 | NM_133315 | 170840 | *Slc40a1* |  | 1.94 |  |  |  |  |
| A_44_P823790 | XM_008772321 | 50664 | *Gnao1* |  |  |  | 1.61 |  |  |
| A_44_P824812 |  |  |  |  |  |  |  |  | -1.80 |
| A_44_P839849 | XM_006249933 | 289051 | *Dennd1b* |  | -1.66 |  |  |  |  |
| A_44_P856998 |  |  |  |  | 1.68 |  |  |  |  |
| A_44_P883154 | NM_019149 | 29150 | *Matr3* |  | -1.98 |  |  |  |  |
| A_44_P886074 | XM_006250875 | 305288 | *Cep135* |  |  |  |  |  | -1.66 |
| A_44_P889987 |  |  |  | -1.54 |  |  |  |  |  |
| A_44_P915286 | XM_008762909 | 500131 | *Creb5* |  | -2.05 |  |  |  |  |
| A_44_P928928 |  |  |  |  | 1.82 |  |  |  |  |
| A_44_P932059 | NM_001109381 | 679890 | *Gpkow* |  | 1.51 |  |  |  |  |
| A_44_P935273 | NM_001009349 | 300886 | *Mthfs* |  |  |  |  |  | -1.74 |
| A_44_P940953 |  |  |  | -1.54 |  |  |  |  |  |
| A_44_P944159 |  |  |  |  | 1.61 |  |  |  |  |
| A_44_P958227 |  |  |  | 1.52 |  |  |  |  |  |
| A_44_P958404 | NM_053481 | 85243 | *Pik3cb* |  | 1.55 |  |  |  |  |
| A_44_P959220 | NM_001109103 | 498534 | *RGD1565212* | 1.62 |  |  |  |  |  |
| A_44_P959463 |  |  |  |  | 1.94 |  |  |  |  |
| A_44_P959944 | NM_001136261 | 100192313 | *Atxn7l3b* |  | -1.76 |  |  |  |  |
| A_44_P966141 | XM_006253805 | 306842 | *Gfod1* |  |  |  |  |  | 1.52 |
| A_44_P974107 | NM_001108319 | 360720 | *Rabl3* |  | 1.51 |  |  |  |  |
| A_44_P976336 | XM_006251728 | 361015 | *Atxn7* |  | -1.64 |  |  |  |  |
| A_44_P980148 |  |  |  | -1.66 |  |  |  |  |  |
| A_44_P982636 |  |  |  | 1.65 |  |  |  |  |  |
| A_44_P989457 |  |  |  | -1.51 |  |  |  |  |  |
| A_44_P990142 | NM_001005533 | 288416 | *N4bp2l2* |  | -1.69 |  |  |  |  |
| A_44_P992578 | NM_001025740 | 362720 | *Rrm2* |  | 1.52 |  |  |  |  |
| A_44_P994550 | NM_001106643 | 297969 | *Rars2* |  | 1.51 |  |  |  |  |
| **(B) Rat Palate Day 91** | | | | | | | | | |
| A_42_P485589 | NM_017246 | 29568 | *Map2k5* | 2.48 |  |  |  |  |  |
| A_42_P498708 | NM_024367 | 79208 | *Epha5* |  |  |  | -1.69 |  |  |
| A_42_P509876 | NM_001109065 | 498179 | *RGD1563482* |  |  |  |  |  | -1.56 |
| A_42_P529780 | NM_138855 | 192209 | *Spdya* |  |  |  |  |  | 1.68 |
| A_42_P540711 | NM_133568 | 171099 | *Rasd2* |  |  |  | -1.76 |  |  |
| A_42_P545100 | NM_001009643 | 292875 | *Aspdh* | 1.66 |  |  |  |  |  |
| A_42_P553706 | NM_001009533 | 494320 | *Ccdc8* |  |  |  |  | 1.64 |  |
| A_42_P598679 | NM_001107564 | 309135 | *Ano1* |  | 1.78 |  |  |  |  |
| A_42_P623666 | NM_001122947 | 301295 | *Efhc1* |  |  |  | 2.02 |  |  |
| A_42_P623925 | NM_001106564 | 296608 | *RGD1306233* |  |  |  | 1.52 |  |  |
| A_42_P686427 |  |  |  |  |  |  | 2.12 |  |  |
| A_42_P702242 | NM_001170563 | 306790 | *Sema4d* | -1.68 |  |  |  |  |  |
| A_42_P713089 | XM_006234221 |  |  | -1.58 |  |  |  |  |  |
| A_42_P745356 | NM_080403 | 140586 | *Sox9* | -1.83 |  |  |  |  |  |
| A_42_P760326 | NM_001011945 | 294336 | *Pcbp3* |  |  |  | -1.56 |  |  |
| A_42_P762924 | XM_001075834 |  |  | -1.59 |  |  |  |  |  |
| A_42_P763224 | NM_001107561 | 309100 | *Scgb1c1* |  |  |  | 2.73 |  |  |
| A_42_P825274 | NM_130431 | 161476 | *Hspb2* |  |  |  |  | -1.50 |  |
| A_42_P833106 | NM_001134520 | 363135 | *Rad54l2* | -1.78 |  |  |  |  |  |
| A_42_P836063 | NM_001108121 | 315394 | *Kbtbd3* | 1.51 |  |  |  |  |  |
| A_43_P11674 | NM_013039 | 25559 | *Abcc8* | 1.59 |  |  |  |  |  |
| A_43_P12614 | NM_031613 | 58814 | *Tmod2* |  | -1.51 |  |  |  |  |
| A_43_P12707 | NM_031975 | 83801 | *Ptms* | -1.93 |  |  |  |  |  |
| A_43_P13086 | NM_080780 | 113995 | *P2rx5* |  |  |  | 1.63 |  |  |
| A_43_P13386 | NM_139333 | 246216 | *Prpf19* |  |  |  |  | 1.67 |  |
| A_43_P14867 | NM_001106194 | 292077 | *Sult5a1* |  | 1.58 |  |  |  |  |
| A_43_P15669 | NM_019330 | 687008 | *Pcca* |  | 1.85 |  |  |  |  |
| A_43_P16128 | NM_001012219 | 365963 | *Lhx8* |  | -2.67 |  |  |  |  |
| A_43_P16203 | XM_008771022 | 252959 | *Dnah12* |  |  |  | 3.02 |  |  |
| A_43_P16277 |  |  |  | 1.68 |  |  |  |  |  |
| A_43_P16636 | NM_001011978 | 298541 | *Dhdds* |  | 1.54 |  |  |  |  |
| A_43_P17458 | NM_001014105 | 317191 | *Apool* | 1.51 |  |  |  |  |  |
| A_43_P17545 | XM_008776256 | 690217 | *Pcnxl4* |  |  |  |  |  | -1.59 |
| A_43_P17569 | XM_341223 | 360945 | *Sel1l3* |  |  |  | -1.73 |  |  |
| A_43_P17650 | NM_001034093 | 365668 | *Skiv2l2* |  | -2.25 |  |  |  |  |
| A_43_P17786 | NM_001025048 | 500281 | *Setmar* | 1.58 |  |  |  |  |  |
| A_43_P17914 | NM_001191818 | 299186 | *Dpf3* |  | -2.06 |  |  |  |  |
| A_43_P18361 | NM_001107074 | 303747 | *Cd7* |  |  |  | 1.57 |  |  |
| A_43_P18523 | NM_001034924 | 304431 | *Sfswap* | -1.55 |  |  |  |  |  |
| A_43_P18653 | XM_003751494 |  |  |  | -1.54 |  |  |  |  |
| A_43_P19065 | XM_006223621 |  |  |  |  |  |  |  | -1.73 |
| A_43_P19640 | XM_006234485 |  |  | -1.59 |  |  |  |  |  |
| A_43_P19769 | NM_001108706 | 362727 | *Efcab10* |  |  |  | 2.49 |  |  |
| A_43_P20314 | XM_008759207 |  |  | -1.52 |  |  |  |  |  |
| A_43_P20539 | NM_001012142 | 360612 | *Scrn2* | 1.68 |  |  |  |  |  |
| A_43_P20567 | NM_001107431 | 307859 | *Chst5* |  | 1.77 |  |  |  |  |
| A_43_P21114 | NM_053392 | 84407 | *Cdh11* |  | 1.53 |  |  |  |  |
| A_43_P21165 | NM_001015029 | 362607 | *Kpna6* |  | 1.56 |  |  |  |  |
| A_43_P21348 | XM_001077762 |  |  |  |  |  | -1.62 |  |  |
| A_43_P21800 | NM_001024254 | 304530 | *Taok3* | -1.64 |  |  |  |  |  |
| A_43_P22080 | NM_001012044 | 306071 | *Lcp1* |  |  |  | 1.74 |  |  |
| A_43_P22189 |  |  |  |  | 1.58 |  |  |  |  |
| A_43_P22280 | NM_001191584 | 316740 | *Myom1* |  |  |  |  | -1.71 |  |
| A_43_P23463 |  |  |  |  | -1.80 |  |  |  |  |
| A_44_P1020467 | XM_008767599 |  |  | -1.56 |  |  |  |  |  |
| A_44_P1023847 | NM_001191634 | 309451 | *Gbf1* | -1.63 |  |  |  |  |  |
| A_44_P1027964 | XM_008767356 |  |  |  |  |  | 2.63 |  |  |
| A_44_P1028477 | NM_001107196 | 304962 | *Atf6* | -1.80 |  |  |  |  |  |
| A_44_P1031380 | NM_001135158 | 287408 | *Myh1* |  |  |  |  | -2.61 |  |
| A_44_P1031457 | NM_181694 | 296610 | *Rsb66* |  |  |  | 1.94 |  |  |
| A_44_P103170 | NM_001107910 | 313027 | *Eya3* | -1.62 |  |  |  |  |  |
| A_44_P1037806 | NM_053318 | 58917 | *Hpx* |  |  |  | -1.57 |  |  |
| A_44_P1039444 | NM_032613 | 29278 | *Lasp1* |  | -2.50 |  |  |  |  |
| A_44_P104221 |  |  |  | 1.51 |  |  |  |  |  |
| A_44_P1044878 | NM_001100719 | 314633 | *Ap3d1* | -1.84 |  |  |  |  |  |
| A_44_P1045820 | XM_008774195 |  |  |  |  |  | 1.80 |  |  |
| A_44_P1049236 | NM_001034928 | 312444 | *Immt* | -1.57 |  |  |  |  |  |
| A_44_P1051765 | NM_001108695 | 362667 | *Thap3* | -1.51 |  |  |  |  |  |
| A_44_P1052817 |  |  |  | 1.58 |  |  |  |  |  |
| A_44_P1053578 | NM_001012134 | 316583 | *B3gnt7* | -2.09 |  |  | -2.30 | -1.99 |  |
| A_44_P1056788 | NM_001109433 | 680945 | *Sdf2l1* | -2.01 |  |  |  |  |  |
| A_44_P106396 |  |  |  | 1.84 |  |  |  |  |  |
| A_44_P106566 |  |  |  | 2.09 |  |  |  |  |  |
| A_44_P108682 | NM_057116 | 117256 | *Ppp2r2c* |  |  |  | 1.90 |  |  |
| A_44_P110491 |  |  |  |  | -2.02 |  |  |  |  |
| A_44_P111941 | NM_053767 | 114767 | *Ptpre* |  | -2.27 |  |  |  |  |
| A_44_P114034 | NM_001034125 | 287422 | *Per1* |  | 2.02 |  |  |  |  |
| A_44_P115932 | NM_001036626 | 298765 | *Zfp36l2* | -1.51 |  |  |  |  |  |
| A_44_P118326 |  |  |  | 1.54 |  |  |  |  |  |
| A_44_P118415 |  |  |  |  |  |  | 2.01 |  |  |
| A_44_P119499 | NM_001191107 | 690450 | *Taf1b* | 1.53 |  |  |  |  |  |
| A_44_P121039 | NM_172008 | 29144 | *Canx* |  | 1.63 |  |  |  |  |
| A_44_P121875 | XM_001075066 | 300381 | *RGD1304745* |  | -1.77 |  |  |  |  |
| A_44_P123119 |  |  |  |  |  |  | -1.59 |  |  |
| A_44_P125160 |  |  |  | 1.68 |  |  |  |  |  |
| A_44_P128554 | XM_008771787 | 498764 | *RGD1562037* |  |  |  | 2.14 |  |  |
| A_44_P129298 | NM_001135868 | 304785 | *Slc45a3* |  | 1.93 |  |  |  |  |
| A_44_P133226 | NM_001134863 | 306459 | *Stox2* | -1.59 |  |  |  |  |  |
| A_44_P133386 | XM_006236581 | 362375 | *Lancl2* |  |  |  |  | -1.60 |  |
| A_44_P135554 |  |  |  |  | -1.73 |  |  |  |  |
| A_44_P138495 |  |  |  |  |  |  | -1.52 |  |  |
| A_44_P139232 |  |  |  | 1.60 |  |  |  |  |  |
| A_44_P149370 | NM_198733 | 292728 | *Cyp2b21* |  | 1.98 |  |  |  |  |
| A_44_P152120 | NM_001014151 | 361351 | *Hdhd2* |  | -2.00 |  |  |  |  |
| A_44_P154793 | NM_001014230 | 363796 | *Iqcg* |  |  |  | 2.45 |  |  |
| A_44_P159061 |  |  |  |  |  |  |  |  | 1.75 |
| A_44_P162956 |  |  |  |  | -1.92 |  |  |  |  |
| A_44_P164804 |  |  |  | 1.80 |  |  |  |  |  |
| A_44_P166206 | NM_001024260 | 311111 | *Nostrin* |  |  |  | 1.64 |  |  |
| A_44_P169917 |  |  |  | 1.71 |  |  |  |  |  |
| A_44_P173151 | NM_001012039 | 305604 | *Efemp1* | 1.50 |  |  |  |  |  |
| A_44_P175041 | NM_145775 | 252917 | *Nr1d1* |  |  |  |  | 1.66 | 1.73 |
| A_44_P175654 |  |  |  |  | 1.62 |  |  |  |  |
| A_44_P179046 |  |  |  | 1.55 |  |  |  |  |  |
| A_44_P179999 | XM_006255125 | 83686 | *Cngb1* | -1.82 |  |  |  |  |  |
| A_44_P181927 |  |  |  |  |  |  |  | -1.90 |  |
| A_44_P184909 |  |  |  |  |  |  |  | -1.69 |  |
| A_44_P188950 |  |  |  | 1.89 |  |  |  |  |  |
| A_44_P190639 | XM_002724507 | 117251 | *Dnah9* |  |  |  | 2.46 |  |  |
| A_44_P191962 | NM_001007734 | 361465 | *Echdc1* |  | 2.00 |  |  |  |  |
| A_44_P192543 | NM_001013105 | 300211 | *Fkbp11* |  |  |  | -1.53 |  |  |
| A_44_P196146 | NM_031349 | 83518 | *Aplnr* |  |  |  |  |  | -1.56 |
| A_44_P200661 |  |  |  | 1.63 |  |  |  |  |  |
| A_44_P200824 |  |  |  |  |  |  | -1.53 |  |  |
| A_44_P201883 | NM_001107040 | 303469 | *Dlx4* |  |  |  | -1.59 |  |  |
| A_44_P203914 |  |  |  | -1.54 |  |  |  |  |  |
| A_44_P208930 | NM_001034917 | 287612 | *Mks1* |  |  |  | 1.71 |  |  |
| A_44_P215459 | NM_001107699 | 310683 | *Itga10* |  | -1.68 |  |  |  |  |
| A_44_P216782 |  |  |  | 1.69 |  |  |  |  |  |
| A_44_P219055 |  |  |  |  |  |  | -1.64 |  |  |
| A_44_P220696 |  |  |  |  | -1.95 |  |  |  |  |
| A_44_P221437 | NM_001047855 | 293774 | *Dtx4* |  |  |  | -1.98 |  |  |
| A_44_P221461 |  |  |  |  |  |  |  |  | -1.59 |
| A_44_P230851 |  |  |  | 1.63 |  |  |  |  |  |
| A_44_P232851 |  |  |  | 1.74 |  |  |  |  |  |
| A_44_P233080 |  |  |  |  | -2.04 |  |  |  |  |
| A_44_P233450 |  |  |  | 1.61 |  |  |  |  |  |
| A_44_P233557 | NM_182669 | 293180 | *Micalcl* |  | -1.62 |  |  |  |  |
| A_44_P238756 | XM_008760294 |  |  |  |  |  | 1.71 |  |  |
| A_44_P239985 | NM_013217 | 26955 | *Mllt4* | -1.60 |  |  |  |  |  |
| A_44_P242895 | NM_138867 | 192235 | *Hyou1* | -1.90 |  |  |  |  |  |
| A_44_P243520 | NM_001047902 | 360845 | *Zc3h11a* | -1.78 |  |  |  |  |  |
| A_44_P245019 |  |  |  |  |  |  | 1.71 |  |  |
| A_44_P245530 | NM_053783 | 116465 | *Ifngr1* |  | 1.56 |  |  |  |  |
| A_44_P247213 |  |  |  | 1.72 |  |  |  |  |  |
| A_44_P247810 | NM_001000249 | 293765 | *Olr327* | -1.53 |  |  |  |  |  |
| A_44_P248542 |  |  |  | -1.60 |  |  |  |  |  |
| A_44_P248764 |  |  |  | 1.76 |  |  |  |  |  |
| A_44_P248983 |  |  |  | -1.55 | -1.62 |  |  |  |  |
| A_44_P251124 | NM_001013111 | 301333 | *Ptpn18* |  | 1.89 |  |  |  |  |
| A_44_P253940 | NM_198783 | 362495 | *Ndufaf4* |  |  |  | 1.60 |  |  |
| A_44_P254071 |  |  |  | 1.74 |  |  |  |  |  |
| A_44_P254201 |  |  |  |  | -1.63 |  |  |  |  |
| A_44_P256822 |  |  |  | 1.80 |  |  |  |  |  |
| A_44_P257018 | NM_001106974 | 302920 | *Rbfox1* | 2.07 |  |  |  |  |  |
| A_44_P257522 | NM_033650 | 25582 | *Agrp* |  | -1.94 |  |  |  |  |
| A_44_P265278 | XR_592993 | 298931 | *Akr1b1-ps1* |  | 1.61 |  |  |  |  |
| A_44_P267155 |  |  |  |  | 1.77 |  |  |  |  |
| A_44_P268864 | NM_138502 | 29254 | *Mgll* |  | 2.18 |  |  |  |  |
| A_44_P270952 |  |  |  |  |  |  | 1.73 |  |  |
| A_44_P271978 |  |  |  |  |  |  |  |  | 1.79 |
| A_44_P280506 |  |  |  | 1.73 |  |  |  |  |  |
| A_44_P286273 | NM_001277334 | 298138 | *Rasef* | 1.57 |  |  |  |  |  |
| A_44_P288287 | NM_001305281 | 297865 | *Dsel* |  |  |  | 1.53 |  |  |
| A_44_P288785 |  |  |  |  |  |  | -1.52 |  |  |
| A_44_P289277 |  |  |  |  | -1.78 |  |  |  |  |
| A_44_P292082 |  |  |  | 1.81 |  |  |  |  |  |
| A_44_P293243 |  |  |  |  |  |  | 1.93 |  |  |
| A_44_P298033 |  |  |  | -1.56 |  |  |  |  |  |
| A_44_P298268 | NM_133421 | 170946 | *Marf1* |  |  |  | -2.12 |  |  |
| A_44_P301560 |  |  |  | -1.84 |  |  |  |  |  |
| A_44_P306339 | NM_012511 | 24218 | *Atp7b* |  |  |  | -1.68 |  |  |
| A_44_P308877 | NM_001005244 | 24907 | *Dmd* |  | -1.95 |  |  |  |  |
| A_44_P312606 |  |  |  |  |  |  |  |  | -1.55 |
| A_44_P315117 |  |  |  | 1.90 |  |  |  |  |  |
| A_44_P315649 | NM_001163156 | 362733 | *Etv1* |  |  |  | -2.05 |  |  |
| A_44_P316194 | NM_017156 | 29295 | *Cyp2b12* |  | 2.28 |  |  |  |  |
| A_44_P324155 | XM_001056810 |  |  |  |  |  | -1.56 |  |  |
| A_44_P329823 |  |  |  |  | -1.87 |  |  |  |  |
| A_44_P332138 |  |  |  | 2.31 |  |  |  |  |  |
| A_44_P332896 | XM_006223411 |  |  |  | 1.53 |  |  |  |  |
| A_44_P335898 | NM_001008863 | 408223 | *Usp54* |  |  |  | 1.66 |  |  |
| A_44_P335974 | NM_017272 | 29651 | *Aldh1a7* |  |  |  | 1.50 |  |  |
| A_44_P338893 |  |  |  |  |  |  |  | -1.76 |  |
| A_44_P341133 | NM_001170459 | 360980 | *Tns3* | -1.63 |  |  |  |  |  |
| A_44_P343768 |  |  |  | 1.59 |  |  |  |  |  |
| A_44_P348614 | NM_001034137 | 301570 | *Sp110* | 1.51 |  |  |  |  |  |
| A_44_P352331 | NM_001077590 | 365603 | *Bves* |  |  |  | -1.54 |  |  |
| A_44_P353003 |  |  |  |  | -1.66 |  |  |  |  |
| A_44_P354507 |  |  |  | 1.86 |  |  |  |  |  |
| A_44_P356962 | NM_001013155 | 309548 | *Shoc2* |  | -1.67 |  |  |  |  |
| A_44_P359684 | NM_001107095 | 304005 | *Nfkbiz* | 1.52 |  |  |  |  |  |
| A_44_P362469 |  |  |  | 1.67 |  |  |  |  |  |
| A_44_P364776 |  |  |  |  | -2.05 |  |  |  |  |
| A_44_P368249 | NM_001173555 | 362508 | *Spag8* |  |  |  | 1.66 |  |  |
| A_44_P371091 |  |  |  |  |  |  |  |  | -1.76 |
| A_44_P375173 | XM_008770656 | 103693841 | *LOC103693841* |  |  |  | 1.66 |  |  |
| A_44_P376364 |  |  |  |  |  |  |  | 1.60 |  |
| A_44_P377107 | XM_008765821 |  |  | 1.59 |  |  |  |  |  |
| A_44_P378129 | XM_008766953 | 316312 | *Zfp451* | 1.57 |  |  |  |  |  |
| A_44_P378383 | NM_001113748 | 300659 | *Rnf26* |  | 1.90 |  |  |  |  |
| A_44_P379699 | XM_006224337 | 63881 | *Rapgef1* | -1.73 |  |  |  |  |  |
| A_44_P381848 | NM_053655 | 114114 | *Dnm1l* | -1.67 |  |  |  |  |  |
| A_44_P383847 |  |  |  |  | -1.57 |  |  |  |  |
| A_44_P386263 |  |  |  | 1.80 |  |  |  |  |  |
| A_44_P387120 | XM_006227365 |  |  | -1.58 |  |  |  |  |  |
| A_44_P393631 |  |  |  | -1.59 |  |  |  |  |  |
| A_44_P395073 |  |  |  | 1.52 |  |  |  |  |  |
| A_44_P403415 | NM_001130569 | 362971 | *Mpped1* | 2.95 |  |  |  |  |  |
| A_44_P403701 | NM_001134635 | 500992 | *Nxpe1* |  |  |  |  | -1.59 |  |
| A_44_P405443 | NM_001031655 | 310864 | *Manba* |  | 1.57 |  |  |  |  |
| A_44_P415298 |  |  |  | -1.73 |  |  |  |  |  |
| A_44_P416751 | NM_001047843 | 287524 | *Rpa1* |  | 1.61 |  |  |  |  |
| A_44_P417470 | XR_594055 | 363087 | *Vps13c* |  | 1.55 |  |  |  |  |
| A_44_P417629 |  |  |  |  |  |  |  | 1.69 |  |
| A_44_P419991 |  |  |  |  | -1.62 |  |  |  |  |
| A_44_P420215 |  |  |  |  | 1.55 |  |  |  |  |
| A_44_P423263 |  |  |  | 2.05 |  |  |  |  |  |
| A_44_P428997 | NM_001107661 | 310244 | *Zc2hc1a* |  |  |  | 1.60 |  |  |
| A_44_P429334 | NM_001134993 | 499566 | *Car13* | -1.79 |  |  |  |  |  |
| A_44_P430208 |  |  |  | 1.53 |  |  |  |  |  |
| A_44_P433526 | XM_003751199 | 100909836 | *LOC100909836* |  |  |  |  |  | -1.52 |
| A_44_P435324 |  |  |  | 1.81 |  |  |  |  |  |
| A_44_P436844 |  |  |  |  | -2.32 |  |  |  |  |
| A_44_P438473 | XM_008761022 | 103691558 | *LOC103691558* |  |  |  | 1.85 |  |  |
| A_44_P446668 |  |  |  | 1.74 |  |  |  |  |  |
| A_44_P448154 | NM_001134980 | 313373 | *Cyp2j10* |  | 1.79 |  |  |  |  |
| A_44_P449567 | NM_181377 | 303311 | *Rtn4rl1* | 1.52 |  |  |  |  |  |
| A_44_P453738 |  |  |  | 1.64 |  |  |  |  |  |
| A_44_P454157 | NM_021598 | 29269 | *Mcpt8* |  |  |  | -1.54 |  |  |
| A_44_P454259 | XR_593200 | 500717 | *RGD1566401* |  |  |  | -1.63 |  |  |
| A_44_P456584 | NM_001001125 | 406046 | *Olr471* | 1.88 |  |  |  |  |  |
| A_44_P460596 |  |  |  | 1.76 |  |  |  |  |  |
| A_44_P460928 |  |  |  | 2.10 |  |  |  |  |  |
| A_44_P462505 |  |  |  | 1.90 |  |  |  |  |  |
| A_44_P463749 | NM_001270954 | 64455 | *Rasd1* |  |  |  |  |  | 1.73 |
| A_44_P464196 | NM_001012164 | 361383 | *Adgre5* |  | 1.58 |  |  |  |  |
| A_44_P468681 | NM_001106444 | 295243 | *Krtcap2* | -1.64 |  |  |  |  |  |
| A_44_P469746 |  |  |  |  | -1.63 |  |  |  |  |
| A_44_P470945 |  |  |  |  | -1.55 |  |  |  |  |
| A_44_P471217 |  |  |  |  | 1.50 |  |  |  |  |
| A_44_P471426 | NM_001012204 | 363286 | *Traf3ip1* |  |  |  | 1.78 |  |  |
| A_44_P473499 | NM_001109016 | 367786 | *Bmx* | 1.67 |  |  |  |  |  |
| A_44_P474900 | NM_001013875 | 289753 | *Rhbdd3* |  |  |  |  |  | -1.60 |
| A_44_P476936 |  |  |  | -1.68 |  |  |  |  |  |
| A_44_P478534 | XR_589202 | 103690250 | *LOC103690250* |  |  |  |  |  | 1.57 |
| A_44_P480939 | NM_001107008 | 303212 | *Zfp287* | -1.69 |  |  |  |  |  |
| A_44_P482444 | NM_001287614 | 102551365 | *LOC102551365* |  | -1.83 |  |  |  |  |
| A_44_P483152 | XM_234236 |  |  |  |  |  |  |  | 1.64 |
| A_44_P484826 | NM_017276 | 29662 | *Gdi2* | 1.62 |  |  |  |  |  |
| A_44_P485462 | XM_006225533 | 313040 | *Csmd2* |  |  |  | -1.74 |  |  |
| A_44_P489244 | NM_147144 | 259170 | *Casc3* | 1.60 |  |  |  |  |  |
| A_44_P493005 | NM_022858 | 64826 | *Foxq1* | -1.79 |  |  |  |  |  |
| A_44_P495236 |  |  |  |  | -2.08 |  |  |  |  |
| A_44_P502022 |  | 289315 | *Sde2* |  | -1.64 |  |  |  |  |
| A_44_P504120 | NM_001107990 | 313663 | *Crocc* |  | 1.53 |  |  |  |  |
| A_44_P506166 |  |  |  |  |  |  | -1.82 |  |  |
| A_44_P508901 | NM_001014273 | 367902 | *Armcx3* | 2.20 |  |  |  |  |  |
| A_44_P509070 |  |  |  |  |  |  | -1.89 |  |  |
| A_44_P513877 |  |  |  |  | 1.57 |  |  |  |  |
| A_44_P513903 |  |  |  | 1.94 |  |  |  |  |  |
| A_44_P515119 |  |  |  | 1.72 |  |  |  |  |  |
| A_44_P519465 |  |  |  |  |  |  |  |  | -1.69 |
| A_44_P521505 |  |  |  | 1.59 |  |  |  |  |  |
| A_44_P522891 | XM_006251682 | 102547093 | *LOC102547093* |  | -1.61 |  |  |  |  |
| A_44_P523294 | XM_006220702 |  |  |  | 1.94 |  |  |  |  |
| A_44_P526552 |  |  |  | 1.67 |  |  |  |  |  |
| A_44_P527858 |  |  |  |  | 1.58 |  |  |  |  |
| A_44_P528965 |  |  |  | 1.54 |  |  |  |  |  |
| A_44_P528988 |  |  |  |  | 1.68 |  |  |  |  |
| A_44_P539917 |  |  |  | 1.62 |  |  |  |  |  |
| A_44_P541692 | NM_001107054 | 303580 | *Hexim2* |  |  |  |  |  | -1.59 |
| A_44_P543711 |  |  |  | -1.50 |  |  |  |  |  |
| A_44_P544134 |  |  |  |  | -1.85 |  |  |  |  |
| A_44_P544784 |  |  |  |  |  |  |  |  | 1.80 |
| A_44_P546000 |  |  |  | -1.54 |  |  |  |  |  |
| A_44_P548903 |  |  |  |  | -2.13 |  |  |  |  |
| A_44_P551963 |  |  |  | -1.56 |  |  |  |  |  |
| A_44_P552181 |  |  |  |  |  |  | -2.05 |  |  |
| A_44_P552624 | NM_001000222 | 293402 | *Olr259* | 1.54 |  |  |  |  |  |
| A_44_P558157 | NM_001122947 | 301295 | *Efhc1* |  |  |  | 1.63 |  |  |
| A_44_P558666 |  |  |  |  |  |  |  | 1.51 |  |
| A_44_P560207 |  |  |  |  | -1.64 |  |  |  |  |
| A_44_P596814 |  |  |  |  | -1.50 |  |  |  |  |
| A_44_P602851 | XM_008773651 | 363509 | *Arhgef6* |  |  |  |  |  | -1.56 |
| A_44_P609668 |  |  |  |  | -1.66 |  |  |  |  |
| A_44_P622265 |  |  |  | 1.53 |  |  |  |  |  |
| A_44_P623737 |  |  |  |  |  |  | -1.54 |  |  |
| A_44_P640345 | XM_001076997 | 367181 | *Gadl1* |  |  |  |  | -1.76 |  |
| A_44_P643560 |  |  |  |  |  |  | -1.61 |  |  |
| A_44_P645108 | NM_138519 | 171548 | *Dkk3* |  |  |  | 1.52 |  |  |
| A_44_P645975 |  |  |  | 1.82 |  |  |  |  |  |
| A_44_P649412 |  |  |  |  | -2.07 |  |  |  |  |
| A_44_P655502 |  |  |  | 1.57 |  |  |  |  |  |
| A_44_P655613 | NM_001134747 | 684961 | *Zmat4* |  |  |  | 2.16 |  |  |
| A_44_P680689 |  |  |  |  |  |  | -1.69 |  |  |
| A_44_P682849 | XM_001081465 |  |  |  | 1.50 |  |  |  |  |
| A_44_P685542 |  |  |  | 1.80 |  |  |  |  |  |
| A_44_P700917 |  |  |  | -1.55 |  |  |  |  |  |
| A_44_P711321 |  |  |  | 1.60 |  |  |  |  |  |
| A_44_P716741 |  |  |  | 1.53 |  |  |  |  |  |
| A_44_P716867 | NM_001025772 | 500566 | *Stpg1* |  |  |  | 1.58 |  |  |
| A_44_P728688 | XM_006221127 |  |  |  | -1.93 |  |  |  |  |
| A_44_P732571 | XM_008762074 | 100362814 | *LOC100362814* |  | -1.82 |  |  |  |  |
| A_44_P742668 |  |  |  |  |  |  | -1.61 |  |  |
| A_44_P744605 | NM_001047887 | 311013 | *Arsj* |  |  |  |  |  | 1.80 |
| A_44_P746860 | NM_001106277 | 293061 | *Saxo2* |  |  |  | 1.84 |  |  |
| A_44_P760315 |  |  |  | 1.62 |  |  |  |  |  |
| A_44_P762089 | NM_001277394 | 685890 | *Srrm3* |  |  |  |  | -1.67 |  |
| A_44_P775907 |  |  |  |  |  |  | 1.93 |  |  |
| A_44_P782378 |  |  |  |  |  |  | 1.69 |  |  |
| A_44_P806961 |  |  |  | 1.69 |  |  |  |  |  |
| A_44_P810617 | XM_006226362 | 315530 | *Arhgap32* | 1.61 |  |  |  |  |  |
| A_44_P821544 | XM_001062582 |  |  |  | -2.21 |  |  |  |  |
| A_44_P821851 |  |  |  | 2.26 |  |  |  |  |  |
| A_44_P823790 | XM_008772321 | 50664 | *Gnao1* |  |  |  | 1.61 |  |  |
| A_44_P837764 | XM_006235066 | 311437 | *Rassf2* | 1.52 |  |  |  |  |  |
| A_44_P845395 |  |  |  | 1.63 |  |  |  |  |  |
| A_44_P853300 |  |  |  | -1.63 |  |  |  |  |  |
| A_44_P853694 |  |  |  |  |  |  | 1.69 |  |  |
| A_44_P858839 |  |  |  |  | -1.56 |  |  |  |  |
| A_44_P865695 |  |  |  |  |  |  | 2.36 |  |  |
| A_44_P883146 | NM_001007682 | 305889 | *Thtpa* |  | 1.53 |  |  |  |  |
| A_44_P886074 | XM_006250875 | 305288 | *Cep135* |  |  |  |  |  | -1.66 |
| A_44_P890340 |  |  |  |  |  |  | 2.71 |  |  |
| A_44_P899127 | NM_001024334 | 500300 | *LOC500300* |  |  |  | 2.09 |  |  |
| A_44_P902018 | NM_001106940 | 302378 | *Lpar4* | 2.22 |  |  |  |  |  |
| A_44_P917549 |  |  |  |  | -1.53 |  |  |  |  |
| A_44_P921994 |  |  |  |  | -1.90 |  |  |  |  |
| A_44_P930192 |  |  |  |  | -1.96 |  |  |  |  |
| A_44_P933802 | XM_003750604 | 301065 | *Ttc21a* |  |  |  | 1.93 |  |  |
| A_44_P937141 |  |  |  | 1.53 |  |  |  |  |  |
| A_44_P963185 |  |  |  |  |  |  | -1.71 |  |  |
| A_44_P970555 |  |  |  | 1.54 |  |  |  |  |  |
| A_44_P973186 | NM_001164060 | 85385 | *Shc1* | -1.51 |  |  |  |  |  |
| A_44_P973964 |  |  |  |  | -1.97 |  |  |  |  |
| A_44_P979417 | XM_006227465 | 680282 | *Tceal5* |  |  |  | 1.97 |  |  |
| A_44_P982738 |  |  |  |  | -1.77 |  |  |  |  |
| A_44_P985640 |  |  |  |  |  |  | -1.58 |  |  |
| A_44_P986792 | NM_053519 | 300652 | *Sorl1* | 1.52 |  |  |  |  |  |
| A_44_P997444 | NM_001108536 | 361856 | *Tube1* | 1.85 |  |  |  |  |  |
| A_44_P999115 | NM_001012216 | 365360 | *Tmc5* | -1.74 |  |  |  |  |  |
| **(C) Mouse Palate Day 8** | | | | | | | | | |
| A_51_P100856 | NM_010233 | 14268 | *Fn1* |  |  |  | 1.68 |  |  |
| A_51_P101545 | NM_019447 | 54426 | *Hgfac* |  |  |  |  |  | 1.51 |
| A_51_P102257 |  |  |  |  |  |  | 1.51 |  |  |
| A_51_P103007 | NM_008401 | 16409 | *Itgam* |  |  |  |  |  | -1.68 |
| A_51_P103397 | NM_011708 | 22371 | *Vwf* |  | 1.60 |  | 1.96 |  |  |
| A_51_P103406 | NM_011708 | 22371 | *Vwf* |  | 1.53 |  | 1.83 |  |  |
| A_51_P105927 | NM_001033158 | 70784 | *Rasl12* |  |  |  |  |  | 1.68 |
| A_51_P106538 | NM_030127 | 78558 | *Htra3* |  |  |  | 1.75 |  | 1.61 |
| A_51_P107315 | NM_145423 | 216225 | *Slc5a8* |  | 1.94 |  |  |  |  |
| A_51_P108581 | NM_177078 | 320129 | *Adrbk2* |  |  |  |  |  | 1.54 |
| A_51_P109548 |  | 70311 | *2510042O18Rik* | -1.56 |  |  |  |  |  |
| A_51_P111492 | NM_017373 | 18030 | *Nfil3* |  |  | 1.62 |  |  |  |
| A_51_P112308 | NM_026931 | 69068 | *1810011O10Rik* |  |  |  |  |  | -1.59 |
| A_51_P113403 | NM_178743 | 268512 | *Slc26a11* |  |  |  | 1.53 |  |  |
| A_51_P114314 | NM_008748 | 18218 | *Dusp8* |  |  |  |  | -1.73 |  |
| A_51_P116906 | NM_144850 | 223864 | *Rapgef3* |  |  |  |  |  | 1.82 |
| A_51_P117952 | NM_010479 | 193740 | *Hspa1a* |  |  | 2.69 |  |  |  |
| A_51_P118237 | NM_011361 | 20393 | *Sgk1* |  |  |  |  | -2.15 |  |
| A_51_P118527 |  |  |  |  |  |  |  |  | 1.73 |
| A_51_P122030 | NM_013728 | 27216 | *Olfr154* |  |  |  |  | -1.54 |  |
| A_51_P122246 | NM_029720 | 76737 | *Creld2* |  |  | 2.05 |  |  |  |
| A_51_P123655 | NM_008438 | 16545 | *Kera* |  | 3.51 |  |  |  | 3.78 |
| A_51_P124254 | NM_009931 | 12826 | *Col4a1* |  |  |  | 1.74 |  |  |
| A_51_P124315 | XM_006498501 |  |  |  |  |  |  |  | 2.17 |
| A_51_P124550 | NM_139292 | 70335 | *Reep6* |  |  |  |  |  | 1.66 |
| A_51_P125607 | NM_176954 | 319586 | *Celf5* | 1.92 |  |  |  |  |  |
| A_51_P126437 | NM_007930 | 13803 | *Enc1* |  | 1.55 | 1.63 |  |  | 1.76 |
| A_51_P127297 | NM_008288 | 15483 | *Hsd11b1* |  |  |  |  |  | 1.90 |
| A_51_P127475 | NM_183175 | 239126 | *C1qtnf9* |  |  |  |  |  | 1.86 |
| A_51_P128491 | NM_015749 | 21452 | *Tcn2* |  |  |  |  |  | 1.81 |
| A_51_P129464 | NM_009128 | 20250 | *Scd2* |  |  | -1.56 | -1.54 |  |  |
| A_51_P130028 | NM_013509 | 13807 | *Eno2* |  |  |  |  | 1.50 |  |
| A_51_P130332 | NM_028306 | 72630 | *Hspa12b* |  |  |  |  |  | 1.55 |
| A_51_P130957 | NM_030198 | 78833 | *Gins3* |  |  |  | -1.61 |  |  |
| A_51_P131408 | NM_013749 | 27279 | *Tnfrsf12a* |  |  | 1.71 |  |  |  |
| A_51_P134228 | NM_172563 | 217082 | *Hlf* |  | -1.78 |  |  |  |  |
| A_51_P137322 | NM_031161 | 12424 | *Cck* |  |  |  | -1.73 |  |  |
| A_51_P138378 | NM_008036 | 14282 | *Fosb* |  |  | 4.39 |  |  |  |
| A_51_P138744 | NM_019933 | 19258 | *Ptpn4* |  |  |  | -1.57 |  |  |
| A_51_P142813 | NM_025711 | 66695 | *Aspn* |  |  | -1.63 |  |  |  |
| A_51_P142972 | NM_029947 | 77630 | *Prdm8* |  |  |  | 1.57 |  | 2.04 |
| A_51_P143162 | NM_080728 | 140781 | *Myh7* |  |  |  | 2.54 |  | 2.84 |
| A_51_P144349 | NM_172442 | 207521 | *Dtx4* |  | 1.69 |  |  |  |  |
| A_51_P147942 | NM_010512 | 16000 | *Igf1* |  |  |  |  | -1.82 |  |
| A_51_P148105 | NM_011234 | 19361 | *Rad51* |  |  |  |  | -1.53 |  |
| A_51_P151722 |  |  |  |  | 1.51 |  | 1.58 |  |  |
| A_51_P151862 | NM_144862 | 225341 | *Lims2* |  |  |  | 1.97 |  |  |
| A_51_P153423 | NM_001081416 | 68655 | *Fndc1* |  |  |  | 1.53 |  | 1.70 |
| A_51_P154513 | NM_145837 | 239114 | *Il17d* |  |  |  |  |  | 1.58 |
| A_51_P154596 | NM_008721 | 18146 | *Npdc1* |  |  |  |  |  | 1.73 |
| A_51_P156218 | NM_001081178 | 224792 | *Adgrf5* |  |  |  | 1.52 |  |  |
| A_51_P156955 | NM_013459 | 11537 | *Cfd* |  |  |  | 3.45 |  |  |
| A_51_P157193 | NM_023275 | 80837 | *Rhoj* | 1.52 | 1.53 |  |  |  | 1.69 |
| A_51_P157840 | NM_023850 | 76969 | *Chst1* |  |  |  |  |  | 1.82 |
| A_51_P158538 | NM_011756 | 22695 | *Zfp36* |  |  | 2.06 |  |  |  |
| A_51_P159194 | NM_008416 | 16477 | *Junb* |  |  | 1.66 |  |  |  |
| A_51_P159201 | NM_008416 | 16477 | *Junb* |  |  | 1.72 |  |  |  |
| A_51_P159895 | NM_007869 | 13418 | *Dnajc1* |  | 1.58 |  |  |  |  |
| A_51_P160544 | NM_021474 | 58859 | *Efemp2* |  |  | -1.63 |  |  |  |
| A_51_P160870 | NM_194054 | 68585 | *Rtn4* |  |  |  | 1.58 |  |  |
| A_51_P161086 | NM_008908 | 19038 | *Ppic* |  |  |  | 1.71 |  | 2.02 |
| A_51_P161946 | NM_175332 | 103551 | *E130012A19Rik* |  |  |  |  | 1.54 |  |
| A_51_P163624 |  |  |  |  |  |  |  |  | 1.55 |
| A_51_P167527 | NM_008524 | 17022 | *Lum* |  |  | -1.96 |  |  |  |
| A_51_P168894 | NM_178642 | 101772 | *Ano1* |  | 1.79 |  | 1.64 |  |  |
| A_51_P170178 | NM_146184 | 232984 | *B3gnt8* |  |  |  |  | 1.52 |  |
| A_51_P171616 | NM_009518 | 22409 | *Wnt10a* |  |  |  | -1.51 |  |  |
| A_51_P171728 | NM_007543 | 26367 | *Ceacam2* |  | 2.13 |  |  |  |  |
| A_51_P173043 | NM_010118 | 13654 | *Egr2* |  |  | 1.70 |  |  |  |
| A_51_P175303 | NM_019725 | 21886 | *Tle2* |  |  |  | 1.64 |  | 1.75 |
| A_51_P180492 | NM_016974 | 13170 | *Dbp* |  |  |  |  | 2.95 |  |
| A_51_P181565 | NM_010415 | 15200 | *Hbegf* |  |  | 2.06 |  |  |  |
| A_51_P183165 | NM_146129 | 228866 | *Pcif1* |  | -1.89 |  |  |  |  |
| A_51_P183446 | NM_001039186 | 26365 | *Ceacam1* |  | 1.62 |  |  |  |  |
| A_51_P183561 | NM_145473 | 105859 | *Csdc2* |  |  |  |  |  | 2.00 |
| A_51_P183746 |  | 20204 | *Prrx2* |  |  | -1.59 |  |  |  |
| A_51_P184300 | NM_010087 | 13527 | *Dtna* |  |  |  |  |  | 1.68 |
| A_51_P188271 | NM_054042 | 70445 | *Cd248* |  |  |  | 1.56 |  | 1.71 |
| A_51_P189361 | NM_027950 | 71839 | *Osgin1* |  |  |  | -1.52 |  |  |
| A_51_P190111 | NM_008566 | 17218 | *Mcm5* |  |  |  | -1.69 |  |  |
| A_51_P193173 | NM_146118 | 227731 | *Slc25a25* |  |  | 1.51 |  |  |  |
| A_51_P193176 | NM_146118 | 227731 | *Slc25a25* |  |  | 1.64 |  |  |  |
| A_51_P193894 | NM_027375 | 70297 | *Gcc2* |  | 1.95 |  |  |  |  |
| A_51_P195958 | NM_009344 | 21664 | *Phlda1* |  |  | 2.27 |  |  |  |
| A_51_P196687 | NM_008338 | 15980 | *Ifngr2* |  | 1.53 |  |  |  |  |
| A_51_P201174 | NM_026334 | 67717 | *Lipf* |  | 4.30 |  |  |  | 4.34 |
| A_51_P201187 | NM_026385 | 67801 | *Pllp* |  |  |  |  |  | 1.60 |
| A_51_P206165 | NR_027961 | 231253 | *9130230L23Rik* |  |  |  |  |  | 1.82 |
| A_51_P207031 | NM_010876 | 17969 | *Ncf1* |  | 1.62 |  |  |  |  |
| A_51_P210510 | NM_010097 | 13602 | *Sparcl1* |  |  |  | 1.70 |  |  |
| A_51_P210970 | NM_009522 | 22416 | *Wnt3a* |  |  |  |  | 1.86 |  |
| A_51_P211765 | NM_145495 | 225870 | *Rin1* |  |  |  |  | 1.89 |  |
| A_51_P212592 | NM_173749 | 210622 | *Pamr1* |  |  |  | 2.21 |  | 2.23 |
| A_51_P212682 | NM_027290 | 70024 | *Mcm10* |  |  |  | -1.71 | -1.61 |  |
| A_51_P213334 | NM_144919 | 232232 | *Hdac11* |  |  |  | 1.72 |  |  |
| A_51_P214209 | NM_026282 | 67629 | *Spc24* |  |  |  |  | -1.70 |  |
| A_51_P215106 | NM_008047 | 14314 | *Fstl1* |  | 1.53 |  |  |  |  |
| A_51_P216108 | NM_146244 | 238323 | *Rps6kl1* |  |  |  |  |  | 1.96 |
| A_51_P216965 | NM_010221 | 14230 | *Fkbp10* |  |  | -1.53 |  |  |  |
| A_51_P222773 | NM_010446 | 15376 | *Foxa2* |  | 3.07 |  |  |  | 3.14 |
| A_51_P223776 | NM_145434 | 217166 | *Nr1d1* |  |  |  |  | 1.72 |  |
| A_51_P228171 | NM_025495 | 66336 | *Cenpp* |  |  |  |  | -1.61 |  |
| A_51_P228295 | NM_001001880 | 68481 | *Mpzl1* |  | 1.67 |  |  |  | 1.67 |
| A_51_P232771 | NM_025745 | 66753 | *Erlec1* |  |  |  |  |  | 1.89 |
| A_51_P236846 | NM_010171 | 14066 | *F3* |  |  |  |  |  | -1.61 |
| A_51_P239654 | NM_010444 | 15370 | *Nr4a1* |  |  | 5.25 |  |  |  |
| A_51_P239737 | NM_011082 | 18703 | *Pigr* |  | 2.88 |  | 2.52 |  |  |
| A_51_P239984 | NM_012012 | 26909 | *Exo1* |  |  |  | -1.87 |  |  |
| A_51_P240453 | NM_133851 | 108907 | *Nusap1* |  |  |  |  | -1.84 |  |
| A_51_P240501 | NM_026358 | 67749 | *Mgarp* |  |  | -1.74 | -1.72 |  | -1.72 |
| A_51_P240993 | NR_028310 | 75030 | *4930503E24Rik* |  | 1.76 |  |  |  |  |
| A_51_P241995 | NM_016919 | 53867 | *Col5a3* |  |  |  | 1.87 |  |  |
| A_51_P243609 | NM_001163818 | 52040 | *Ppp1r10* |  |  |  |  | -1.64 |  |
| A_51_P244577 | NM_145836 | 238330 | *Irf2bpl* |  |  | 1.61 |  |  |  |
| A_51_P245558 | NM_011326 | 20278 | *Scnn1g* |  |  |  |  |  | -1.79 |
| A_51_P245796 | NM_029083 | 74747 | *Ddit4* |  |  | -1.56 |  |  |  |
| A_51_P246181 |  |  |  | 1.93 |  |  |  |  |  |
| A_51_P248122 | NM_133234 | 170770 | *Bbc3* |  | 1.69 |  | 1.53 | 1.52 | 1.67 |
| A_51_P250217 |  |  |  |  |  |  | 2.38 |  |  |
| A_51_P252157 | NM_011623 | 21973 | *Top2a* |  |  |  |  | -2.17 |  |
| A_51_P252859 | NM_010516 | 16007 | *Cyr61* | -1.56 |  | 1.62 |  |  |  |
| A_51_P255699 | NM_010809 | 17392 | *Mmp3* |  |  |  | 1.69 |  |  |
| A_51_P255765 | NM_001039710 | 67876 | *Coq10b* |  | -1.85 |  |  |  |  |
| A_51_P258570 | NM_011177 | 19144 | *Klk6* |  | -1.68 |  |  |  |  |
| A_51_P259009 | NM_007581 | 12297 | *Cacnb3* |  |  |  |  |  | 1.56 |
| A_51_P262757 | NM_001077348 | 66968 | *Plin5* |  |  | 2.56 |  |  |  |
| A_51_P266861 | NM_008615 | 17436 | *Me1* |  |  |  |  |  | -1.58 |
| A_51_P268193 | NM_017394 | 53896 | *Slc7a10* |  |  |  | 1.80 |  |  |
| A_51_P271503 | NM_008362 | 16177 | *Il1r1* |  |  |  |  | -1.60 |  |
| A_51_P272493 |  |  |  |  |  |  | 1.80 |  |  |
| A_51_P272553 | NM_011498 | 20893 | *Bhlhe40* |  |  |  |  | 1.72 |  |
| A_51_P274259 | NM_001081277 | 229949 | *Ak5* | 1.87 |  |  |  |  |  |
| A_51_P276652 | NM_029397 | 75710 | *Rbm12* |  |  |  |  | 1.51 |  |
| A_51_P278368 | NM_152915 | 227325 | *Dner* |  |  | -1.84 |  |  |  |
| A_51_P278519 | NM_023268 | 104009 | *Qsox1* |  | 1.62 |  |  |  |  |
| A_51_P279038 | NM_008904 | 19017 | *Ppargc1a* |  | 2.00 |  |  |  |  |
| A_51_P279437 | NM_029662 | 76574 | *Mfsd2a* | -1.57 | -1.77 |  |  |  |  |
| A_51_P279606 | NM_009896 | 12703 | *Socs1* |  |  |  |  | 1.66 |  |
| A_51_P282584 | NM_177068 | 320078 | *Olfml2b* |  |  | -1.77 |  |  |  |
| A_51_P282667 | NM_010421 | 15211 | *Hexa* |  |  |  |  |  | 1.56 |
| A_51_P284565 | NM_133706 | 69071 | *Tmem97* |  |  | -1.50 |  |  |  |
| A_51_P284946 | NM_028810 | 74194 | *Rnd3* |  |  | 1.69 |  |  |  |
| A_51_P289482 |  | 101179 | *6430519N07Rik* |  |  |  |  |  | -1.91 |
| A_51_P291713 | NM_134094 | 52589 | *Ncald* |  |  | 2.56 |  |  |  |
| A_51_P292008 | NM_008161 | 14778 | *Gpx3* |  |  |  |  |  | -1.51 |
| A_51_P293938 | NM_026878 | 68939 | *Rasl11b* |  |  |  |  | 1.77 |  |
| A_51_P295085 | NM_008760 | 18295 | *Ogn* |  |  | -1.77 |  |  |  |
| A_51_P295355 | NM_011957 | 26427 | *Creb3l1* |  |  |  |  |  | 1.91 |
| A_51_P296429 | NM_025781 | 66817 | *Tmem170* |  |  |  |  |  | 1.57 |
| A_51_P297993 |  |  |  |  |  | -1.56 |  |  |  |
| A_51_P298107 | NM_028813 | 74199 | *Vit* |  |  |  |  |  | 1.55 |
| A_51_P299858 | NM_028589 | 73634 | *1700125H20Rik* |  |  |  |  |  | 1.91 |
| A_51_P301930 | NM_028977 | 74511 | *Lrrc17* |  |  | -1.77 |  |  |  |
| A_51_P302626 |  | 21405 | *Hnf1a* |  | -1.58 |  |  |  |  |
| A_51_P303332 |  |  |  | -1.60 |  |  |  |  |  |
| A_51_P305770 | NM_145158 | 246707 | *Emilin2* |  |  |  | 1.52 |  | 1.67 |
| A_51_P308796 | NM_010235 | 14283 | *Fosl1* |  |  | 1.84 |  |  |  |
| A_51_P309056 | NM_009868 | 12562 | *Cdh5* |  |  |  |  |  | 1.80 |
| A_51_P314517 | NM_030697 | 80880 | *Kank3* |  |  |  | 1.71 |  |  |
| A_51_P315411 | NM_013813 | 13823 | *Epb4.1l3* |  | -1.54 |  |  |  |  |
| A_51_P316951 | NM_001167860 | 330319 | *Wipf3* |  |  |  |  |  | 1.88 |
| A_51_P321046 | NM_178790 | 320712 | *Abi3bp* |  | -1.71 |  |  |  |  |
| A_51_P321341 | NM_133670 | 20887 | *Sult1a1* |  |  |  | 1.64 |  |  |
| A_51_P321643 | NM_173371 | 100198 | *H6pd* |  |  |  | 1.75 |  |  |
| A_51_P324934 |  | 17215 | *Mcm3* | -1.57 |  |  |  |  |  |
| A_51_P325914 | NM_010591 | 16476 | *Jun* |  |  | 1.58 |  |  |  |
| A_51_P326631 | NM_013811 | 13417 | *Dnah8* |  |  | -1.54 |  |  | -1.56 |
| A_51_P327451 | NM_009653 | 11656 | *Alas2* |  | 3.62 |  |  |  |  |
| A_51_P327511 | NM_008013 | 14190 | *Fgl2* |  | 2.05 |  | 1.94 |  |  |
| A_51_P327564 | NM_009752 | 12091 | *Glb1* |  | 1.63 |  |  |  | 1.76 |
| A_51_P332414 |  | 328277 | *Gm5085* |  | 1.50 |  |  |  |  |
| A_51_P333438 | XM_006511442 |  |  |  |  |  |  |  | 1.69 |
| A_51_P337089 | NM_027014 | 69270 | *Gins1* |  | -1.56 |  |  |  |  |
| A_51_P337246 | NM_023130 | 19383 | *Raly* |  |  | -1.56 |  |  |  |
| A_51_P337355 | NM_012016 | 26918 | *Ern2* |  |  |  |  |  | 2.18 |
| A_51_P338443 | NM_020581 | 57875 | *Angptl4* |  |  |  | 2.46 |  |  |
| A_51_P341041 | NM_183089 | 72107 | *Dscc1* |  |  |  | -1.71 |  |  |
| A_51_P341832 | NM_008743 | 18207 | *Nthl1* | -1.52 |  |  |  |  |  |
| A_51_P343377 | NM_007827 | 13137 | *Daf2* |  |  |  | -1.74 |  |  |
| A_51_P346938 | NM_029796 | 76905 | *Lrg1* | 1.54 | 1.74 |  | 2.01 |  | 1.95 |
| A_51_P349673 | NM_021411 | 58222 | *Rab37* | 1.61 |  |  |  |  |  |
| A_51_P352738 | NM_033564 | 93734 | *Mpv17l* |  |  |  |  |  | 1.64 |
| A_51_P353452 |  |  |  |  |  | 1.78 |  |  |  |
| A_51_P354792 | NM_008521 | 17001 | *Ltc4s* |  |  |  | 1.62 |  |  |
| A_51_P356413 | NM_199068 | 17425 | *Foxk1* |  | -1.53 |  |  |  |  |
| A_51_P358152 | NM_029100 | 74777 | *Sepn1* |  |  |  | 1.56 |  |  |
| A_51_P358633 | NM_010790 | 17279 | *Melk* |  |  |  |  | -1.56 |  |
| A_51_P361220 | NM_008055 | 14366 | *Fzd4* |  |  |  |  |  | 1.66 |
| A_51_P361678 | NM_026599 | 68178 | *Cgnl1* |  |  |  |  |  | 2.07 |
| A_51_P362638 | NM_133977 | 22041 | *Trf* |  |  |  | 1.55 |  |  |
| A_51_P363187 | NM_008176 | 14825 | *Cxcl1* |  |  | 1.61 |  |  |  |
| A_51_P363461 | XR_865717 | 105244000 | *Gm39695* |  | 1.65 |  |  |  |  |
| A_51_P363681 | NM_015776 | 50530 | *Mfap5* |  | 1.70 |  |  |  |  |
| A_51_P364250 | NM_172471 | 209378 | *Itih5* |  |  |  |  |  | 1.56 |
| A_51_P365008 | NM_027884 | 21961 | *Tns1* |  |  |  | 1.51 |  |  |
| A_51_P365138 | NM_027587 | 100504195 | *Micalcl* |  |  |  |  |  | -1.61 |
| A_51_P366000 | NM_176902 | 319370 | *Ubald2* |  |  |  | 1.53 |  |  |
| A_51_P367310 | NM_028083 | 110749 | *Chaf1b* |  | -1.56 |  | -1.51 |  |  |
| A_51_P367866 | NM_007913 | 13653 | *Egr1* |  |  | 7.15 |  |  |  |
| A_51_P371942 | NM_008788 | 18542 | *Pcolce* |  |  | -1.55 |  |  |  |
| A_51_P374468 | NM_008220 | 101488143 | *Hbb-bt* |  | 3.96 |  | 3.89 |  | 3.56 |
| A_51_P374476 | NM_008220 | 101488143 | *Hbb-bt* |  | 1.54 |  | 1.64 | 1.53 | 1.64 |
| A_51_P374752 | NM_011255 | 19662 | *Rbp4* |  |  | -1.73 |  |  | -1.56 |
| A_51_P376934 | NM_019517 | 56175 | *Bace2* |  |  |  |  |  | 2.11 |
| A_51_P378051 | NM_009675 | 11754 | *Aoc3* |  |  |  | 1.67 |  |  |
| A_51_P379385 | NM_153145 | 217258 | *Abca8a* |  |  |  |  |  | 1.77 |
| A_51_P380078 | NM_001122603 | 215384 | *Fcgbp* |  |  |  | 1.88 |  | 2.10 |
| A_51_P380750 | NM_009824 | 12398 | *Cbfa2t3* |  |  |  |  |  | 1.66 |
| A_51_P381440 | NM_009555 | 22700 | *Zfp40* | 1.60 |  |  |  |  |  |
| A_51_P382214 | NM_008998 | 19329 | *Rab17* |  |  |  |  |  | 1.61 |
| A_51_P382369 | NM_175751 | 269023 | *Zfp608* |  |  |  | 1.50 |  |  |
| A_51_P382393 | NM_133753 | 74155 | *Errfi1* |  |  |  |  | -1.53 |  |
| A_51_P382700 | NR_027851 | 67261 | *2900005J15Rik* | 1.50 |  | 1.52 |  |  |  |
| A_51_P383489 | NM_021891 | 60530 | *Fignl1* |  | -1.54 |  | -1.67 |  |  |
| A_51_P383644 | NM_007446 | 11722 | *Amy1* |  |  | 2.93 |  |  |  |
| A_51_P384136 | XM_011246974 |  |  |  |  |  |  | 1.64 |  |
| A_51_P384382 | NM_018776 | 54394 | *Crlf3* |  |  | -1.64 |  |  |  |
| A_51_P385786 | NM_010495 | 15901 | *Id1* |  |  | 1.55 |  |  |  |
| A_51_P387138 | NM_172867 | 242466 | *Zfp462* |  | 1.50 |  |  |  |  |
| A_51_P389864 | NM_178879 | 97440 | *B3gnt9* |  |  | -1.58 |  |  |  |
| A_51_P391367 | NM_031182 | 83383 | *Tfap4* |  |  |  |  | 1.50 |  |
| A_51_P393305 | NM_198034 | 320007 | *Sidt1* |  |  |  |  |  | 1.63 |
| A_51_P397296 | NM_028584 | 73608 | *Marveld3* |  | 1.71 |  |  |  |  |
| A_51_P398683 | NM_026555 | 52377 | *Rcn3* |  |  | -1.64 |  |  |  |
| A_51_P401451 | NM_009862 | 12544 | *Cdc45* |  |  |  | -1.59 |  |  |
| A_51_P403243 | NM_029779 | 76872 | *Ccdc116* |  |  |  |  | 1.94 |  |
| A_51_P404193 | NM_022435 | 64406 | *Sp5* |  |  |  |  |  | 2.07 |
| A_51_P404565 | NM_183027 | 252903 | *Ap1s3* |  | -1.51 |  |  |  |  |
| A_51_P407323 | NM_007976 | 14067 | *F5* |  |  |  |  |  | 2.59 |
| A_51_P408946 | NM_007633 | 12447 | *Ccne1* |  | -1.59 |  |  |  |  |
| A_51_P409311 | NM_199303 | 228796 | *Bpifb6* |  |  |  |  |  | 2.63 |
| A_51_P412846 | NM_028889 | 98363 | *Efhd1* |  |  |  | 1.57 |  | 1.54 |
| A_51_P415059 | NM_011496 | 20877 | *Aurkb* | -1.51 |  |  |  |  |  |
| A_51_P415395 | NM_001081314 | 75697 | *C2cd4b* |  |  | 1.88 |  |  |  |
| A_51_P416126 | NM_146019 | 216848 | *Chd3* |  | 1.86 |  |  |  |  |
| A_51_P419027 |  | 330108 | *Gm20558* |  |  | 1.92 |  |  |  |
| A_51_P420338 |  | 13512 | *Dsg3* |  |  |  | -1.54 | -1.66 |  |
| A_51_P425071 | NM_030888 | 81799 | *C1qtnf3* | 1.67 |  |  |  |  |  |
| A_51_P428582 | NM_009647 | 11639 | *Ak4* |  |  |  | -1.64 |  |  |
| A_51_P428730 |  |  |  |  | -1.67 |  |  |  |  |
| A_51_P430900 | NM_013642 | 19252 | *Dusp1* | -2.57 |  | 2.31 | -2.17 | -2.69 |  |
| A_51_P431179 |  | 74579 | *4833419E13Rik* |  |  |  | -1.62 |  |  |
| A_51_P431888 | NM_178702 | 231858 | *Radil* |  |  |  | 1.55 |  |  |
| A_51_P433228 | NM_009829 | 12444 | *Ccnd2* |  |  |  |  | 1.60 |  |
| A_51_P436051 | NM_025383 | 66147 | *Necap2* |  |  | -1.62 |  |  |  |
| A_51_P436727 | NM_177231 | 109689 | *Arrb1* |  |  |  | 1.56 |  |  |
| A_51_P436861 |  | 213027 | *Evi5l* |  |  |  |  | -1.52 |  |
| A_51_P438200 | NM_030749 | 81500 | *Sil1* |  |  |  |  |  | 1.75 |
| A_51_P440210 | NM_011160 | 19091 | *Prkg1* |  |  |  |  |  | 1.58 |
| A_51_P440790 | NM_031843 | 83768 | *Dpp7* |  | 1.50 |  |  |  |  |
| A_51_P441843 | NM_145946 | 208836 | *Fanci* |  |  |  |  | -1.75 |  |
| A_51_P443902 | NM_008454 | 16615 | *Klk1b16* |  |  | 2.80 |  |  |  |
| A_51_P447976 | NM_001142952 | 74645 | *Fam46c* |  | 2.06 |  |  |  |  |
| A_51_P449777 | NM_022995 | 65112 | *Pmepa1* |  |  |  |  | -1.55 |  |
| A_51_P450623 | NM_009434 | 22113 | *Phlda2* |  |  | 1.69 |  |  |  |
| A_51_P451338 | NM_010588 | 16450 | *Jag2* |  |  |  |  | 1.54 |  |
| A_51_P454152 | NM_001081221 | 319955 | *Ercc6* |  | -1.69 |  |  |  |  |
| A_51_P456113 | NM_030728 | 80982 | *Cemip* |  |  |  |  | -1.77 |  |
| A_51_P456114 |  | 80982 | *Cemip* |  |  |  |  | -1.80 |  |
| A_51_P456721 | NM_013478 | 12007 | *Azgp1* |  | 2.35 |  |  |  | 2.69 |
| A_51_P458384 | NM_175121 | 67760 | *Slc38a2* |  |  |  | 1.55 |  |  |
| A_51_P458451 | NM_009605 | 11450 | *Adipoq* |  |  |  | 2.35 |  |  |
| A_51_P462428 | XM_006519699 |  |  |  |  | 2.46 | 2.12 | 1.79 | 1.61 |
| A_51_P463428 | NM_178149 | 216505 | *Pik3ip1* |  |  |  |  | 1.75 |  |
| A_51_P464387 | NM_030704 | 80888 | *Hspb8* |  |  | 1.62 |  |  |  |
| A_51_P465281 | NM_008495 | 16852 | *Lgals1* |  |  | -1.76 |  |  |  |
| A_51_P466685 | NM_175206 | 74165 | *Fbxl22* |  |  | -1.53 |  |  |  |
| A_51_P468876 | NM_178118 | 330938 | *Dixdc1* |  | 1.78 |  |  |  |  |
| A_51_P470751 | NM_033354 | 89867 | *Sec16b* |  |  |  | 1.68 |  | 1.60 |
| A_51_P474454 | NM_026001 | 67153 | *Rnaseh2b* |  | -1.53 |  |  |  |  |
| A_51_P474459 | NM_007707 | 12702 | *Socs3* |  |  | 1.80 |  |  |  |
| A_51_P475995 | NM_178060 | 21833 | *Thra* |  |  |  | 1.55 | 1.58 |  |
| A_51_P477682 | NM_008939 | 19142 | *Prss12* |  |  |  |  |  | 1.86 |
| A_51_P477850 | NM_011986 | 26562 | *Ncdn* |  |  |  |  |  | 1.55 |
| A_51_P479304 |  | 234267 | *Gpm6a* |  | -1.59 |  |  |  |  |
| A_51_P479321 | NM_080575 | 68738 | *Acss1* |  |  |  |  |  | 2.10 |
| A_51_P480861 | NM_021453 | 58803 | *Pga5* | 1.83 | 1.93 |  |  |  |  |
| A_51_P481325 | NM_010499 | 15936 | *Ier2* |  |  | 1.64 |  |  |  |
| A_51_P482121 | NM_007902 | 13615 | *Edn2* |  |  |  |  | 2.89 |  |
| A_51_P483617 | NM_029554 | 76261 | *0610040J01Rik* |  |  |  |  |  | 2.13 |
| A_51_P484584 |  |  |  |  |  | 1.61 |  |  |  |
| A_51_P489887 | NM_025491 | 66329 | *Susd3* |  |  |  | 1.80 |  |  |
| A_51_P489996 | NM_010081 | 13518 | *Dst* |  |  |  |  | 1.62 |  |
| A_51_P490136 | NM_011896 | 24063 | *Spry1* |  |  | 1.53 |  |  |  |
| A_51_P490286 | NM_145601 | 333329 | *Cngb1* |  |  |  | 1.63 |  |  |
| A_51_P490456 | NM_178373 | 14311 | *Cidec* |  |  |  | 3.04 |  |  |
| A_51_P491350 | NM_009932 | 12827 | *Col4a2* |  |  |  | 1.74 |  |  |
| A_51_P492410 | NM_026784 | 68603 | *Pmvk* |  |  |  |  |  | -1.53 |
| A_51_P492830 | NM_021886 | 26886 | *Cenph* |  |  |  | -1.76 | -1.65 |  |
| A_51_P493522 | NM_178929 | 107250 | *Kazald1* |  |  | -1.56 |  |  |  |
| A_51_P495641 | NM_019641 | 16765 | *Stmn1* |  |  |  |  | -1.55 |  |
| A_51_P495780 | NM_020568 | 57435 | *Plin4* |  |  | 3.96 |  |  |  |
| A_51_P498922 | NM_026106 | 13486 | *Dr1* |  |  |  | -1.50 |  |  |
| A_51_P500984 | NM_008655 | 17873 | *Gadd45b* |  |  |  |  | 1.70 |  |
| A_51_P501844 | NM_175475 | 232174 | *Cyp26b1* |  |  | 1.72 |  |  |  |
| A_51_P502082 | NM_009103 | 20133 | *Rrm1* |  |  |  | -1.58 |  |  |
| A_51_P502203 | NM_001008232 | 230837 | *Asap3* |  |  |  | 1.67 |  | 1.59 |
| A_51_P503162 | NM_011803 | 23849 | *Klf6* |  |  | 1.51 |  |  |  |
| A_51_P503494 | NM_018790 | 11838 | *Arc* |  |  | 2.48 |  |  |  |
| A_51_P505530 | NM_031176 | 81877 | *Tnxb* |  |  |  |  |  | 2.22 |
| A_51_P506045 | NM_176073 | 54381 | *Cpq* |  | 2.03 |  | 2.09 |  | 1.84 |
| A_51_P506204 | NM_009866 | 12552 | *Cdh11* |  |  | -1.59 |  | -1.61 | -1.66 |
| A_51_P507242 | NM_008037 | 14284 | *Fosl2* |  |  | 1.56 |  |  |  |
| A_51_P508088 | NM_029888 | 77264 | *Zfp142* | -1.71 |  |  |  |  |  |
| A_51_P512384 | NM_001044384 | 21857 | *Timp1* |  |  | -1.55 |  |  |  |
| A_51_P514700 | NM_025565 | 66442 | *Spc25* |  |  |  |  | -1.77 |  |
| A_51_P516125 |  |  |  |  |  | 1.69 |  |  |  |
| A_51_P516133 |  |  |  |  |  | 1.59 |  |  |  |
| A_51_P517430 | NM_007639 | 12479 | *Cd1d1* | 1.91 |  |  |  |  |  |
| A_52_P102651 |  | 17169 | *Mark3* | -1.50 |  |  |  |  |  |
| A_52_P103101 | NM_001012324 | 407800 | *Ecm2* |  |  |  | 1.58 |  |  |
| A_52_P105020 | NR_038060 | 76062 | *5830428M24Rik* |  | 1.58 |  |  |  |  |
| A_52_P105537 | NM_010930 | 18133 | *Nov* |  |  |  |  |  | 1.61 |
| A_52_P1076740 | XR_862928 | 105243058 | *Gm39090* |  | -1.54 |  |  |  |  |
| A_52_P10793 | NM_008973 | 19242 | *Ptn* |  |  |  |  | -1.51 |  |
| A_52_P110218 | NM_025769 | 66793 | *Efcab1* | -1.60 |  |  |  |  |  |
| A_52_P1116825 |  |  |  |  |  | -1.53 |  |  |  |
| A_52_P111830 | NM_029956 | 77697 | *Mmab* |  | -1.51 |  |  |  |  |
| A_52_P1132414 |  |  |  |  |  |  | 1.59 |  |  |
| A_52_P1139509 |  |  |  |  |  |  |  |  | 1.78 |
| A_52_P1156269 |  |  |  |  |  |  | -1.69 |  |  |
| A_52_P116204 | NM_001025600 | 54725 | *Cadm1* |  |  |  |  |  | 1.79 |
| A_52_P1164051 |  |  |  |  | 1.51 |  |  |  |  |
| A_52_P1174 | NM_001033416 | 330217 | *Gal3st4* |  |  |  |  | -1.66 |  |
| A_52_P1189908 |  |  |  | 1.58 |  |  |  |  |  |
| A_52_P1196772 |  | 12306 | *Anxa2* |  |  | 1.88 |  |  |  |
| A_52_P121502 | NM_026385 | 67801 | *Pllp* |  |  |  |  |  | 1.79 |
| A_52_P127682 | NM_198114 | 269060 | *Dagla* |  |  |  |  |  | 1.90 |
| A_52_P129140 |  | 18970 | *Polb* |  |  |  |  | -1.59 |  |
| A_52_P130196 |  |  |  |  |  | 1.55 |  |  |  |
| A_52_P13109 |  |  |  |  |  |  |  | 1.66 |  |
| A_52_P135282 | NM_212447 | 73608 | *Marveld3* |  |  |  |  |  | 1.64 |
| A_52_P135455 |  | 71853 | *Pdia6* |  |  |  | -1.67 |  |  |
| A_52_P13802 | NM_009824 | 12398 | *Cbfa2t3* |  |  |  |  |  | 1.64 |
| A_52_P139347 | NM_181412 | 223773 | *Zbed4* |  |  |  | -1.54 |  |  |
| A_52_P148553 | NM_021891 | 60530 | *Fignl1* |  |  |  | -1.53 |  |  |
| A_52_P162298 | NM_026940 | 69101 | *Ydjc* |  |  |  |  | 1.51 |  |
| A_52_P169082 | NM_021294 | 13168 | *Dbil5* |  |  |  | -1.66 | -2.05 |  |
| A_52_P176659 |  |  |  | 1.64 |  |  |  |  | 1.71 |
| A_52_P177324 | NM_010052 | 13386 | *Dlk1* |  |  |  |  | 1.70 |  |
| A_52_P179698 | NM_025412 | 66194 | *Pycrl* |  | -1.50 |  |  |  |  |
| A_52_P180310 |  |  |  |  |  |  |  |  | 1.70 |
| A_52_P18116 | NM_019577 | 56221 | *Ccl24* |  |  |  |  |  | 1.90 |
| A_52_P183368 | NM_011535 | 21386 | *Tbx3* |  |  | 1.57 |  |  |  |
| A_52_P186362 |  |  |  |  |  |  | 1.53 |  |  |
| A_52_P188099 | NM_015828 | 50798 | *Gne* | 2.20 |  |  |  |  |  |
| A_52_P193322 | NM_023348 | 67474 | *Snap29* |  | -1.52 |  |  |  |  |
| A_52_P195922 | NM_028274 | 72544 | *Exosc6* |  | -1.53 |  |  |  |  |
| A_52_P202991 |  |  |  |  | 1.98 |  |  |  |  |
| A_52_P20391 |  |  |  |  | 1.69 |  |  |  |  |
| A_52_P204944 | NM_181541 | 232560 | *Caprin2* | 1.55 |  |  |  |  |  |
| A_52_P208681 |  | 110257 | *Hba-a2* | 2.16 | 2.29 |  | 2.23 | 2.04 | 2.38 |
| A_52_P209264 | NM_027462 | 70560 | *Wars2* |  |  |  | 1.53 |  |  |
| A_52_P211223 | NM_175384 | 108912 | *Cdca2* |  |  | 1.59 |  |  |  |
| A_52_P212650 | NM_053169 | 94092 | *Trim16* |  |  | 1.58 |  |  |  |
| A_52_P213909 | NM_008220 | 101488143 | *Hbb-bt* | 2.25 | 2.49 |  | 2.79 | 2.59 | 3.05 |
| A_52_P214630 | NM_011448 | 20682 | *Sox9* |  |  |  |  |  | 1.97 |
| A_52_P223239 | NR_045312 | 319454 | *Rptoros* |  |  |  |  |  | 2.09 |
| A_52_P223446 |  | 20403 | *Itsn2* | 1.90 |  |  |  |  |  |
| A_52_P223618 | NM_026555 | 52377 | *Rcn3* |  |  | -1.52 |  |  |  |
| A_52_P224801 | NM_007993 | 14118 | *Fbn1* |  |  |  |  |  | 1.57 |
| A_52_P226127 | NM_011805 | 23856 | *Dido1* |  |  |  | 1.51 | 1.67 |  |
| A_52_P226489 |  |  |  |  |  |  | -1.77 |  |  |
| A_52_P232453 | NM_153591 | 244141 | *Nars2* |  |  | 1.65 |  |  |  |
| A_52_P237048 | NR_024139 | 99890 | *Prmt6* |  | -1.54 |  |  |  |  |
| A_52_P241676 | NM_023449 | 65962 | *Slc9a3r2* |  |  |  |  |  | 1.87 |
| A_52_P246229 | NM_199222 | 235416 | *Lman1l* |  |  |  |  |  | 2.76 |
| A_52_P249611 | NM_021313 | 57751 | *Rnf25* |  |  | -1.51 |  |  |  |
| A_52_P250517 | NM_011743 | 20402 | *Zfp106* |  |  |  |  |  | 2.13 |
| A_52_P250803 |  |  |  |  |  |  | -1.52 |  |  |
| A_52_P25193 | NM_146008 | 216198 | *Tcp11l2* |  |  |  | 1.57 |  |  |
| A_52_P257625 | NM_023612 | 71690 | *Esm1* |  |  |  |  |  | 1.55 |
| A_52_P260346 | NM_008220 | 101488143 | *Hbb-bt* | 2.30 | 2.61 |  | 2.41 | 2.25 | 2.82 |
| A_52_P262219 | NM_010234 | 14281 | *Fos* |  |  | 10.26 |  |  |  |
| A_52_P263658 | NM_008236 | 15206 | *Hes2* |  | -1.71 |  |  |  |  |
| A_52_P266132 | NM_008013 | 14190 | *Fgl2* | 1.82 | 1.93 |  |  |  | 1.79 |
| A_52_P266268 |  |  |  |  | -1.57 |  |  |  |  |
| A_52_P275354 | NM_001081434 | 71240 | *Osbpl7* |  |  |  |  |  | 1.51 |
| A_52_P276348 | NM_026945 | 69117 | *Adh6a* |  |  | 1.53 |  |  |  |
| A_52_P276666 | NM_009881 | 12593 | *Cdyl* |  |  |  |  | 1.59 | 1.77 |
| A_52_P278538 | NM_008218 | 15122 | *Hba-a1* | 2.17 | 2.37 |  | 2.22 |  | 2.68 |
| A_52_P279651 | NM_019778 | 56490 | *Zbtb20* |  | 1.52 |  | 1.54 |  |  |
| A_52_P285024 | NM_198247 | 214791 | *Sertad4* |  | 1.52 |  | 1.67 | 1.69 |  |
| A_52_P285182 | NM_021720 | 60364 | *Donson* | -1.58 |  |  |  |  |  |
| A_52_P289835 | NM_183186 | 71375 | *Foxn3* |  |  | 1.55 |  |  |  |
| A_52_P299535 | NM_134117 | 106522 | *Pkdcc* |  |  |  |  |  | 1.73 |
| A_52_P302284 | NM_183366 | 13522 | *Adam28* |  | 1.72 |  |  |  | 1.80 |
| A_52_P302304 | NM_023268 | 104009 | *Qsox1* |  | 1.72 |  |  |  |  |
| A_52_P304902 | NM_010478 | 15511 | *Hspa1b* |  |  | 1.80 |  |  |  |
| A_52_P306065 | XM_011243603 |  |  |  |  | 1.51 |  |  |  |
| A_52_P306305 | NM_001035533 | 11641 | *Akap2* |  | 1.63 |  |  |  |  |
| A_52_P306327 |  | 16000 | *Igf1* |  |  | 1.73 |  |  |  |
| A_52_P309389 | NM_009888 | 12628 | *Cfh* |  |  |  | 1.54 |  |  |
| A_52_P312102 | XM_006518821 |  |  |  |  | 1.53 |  |  |  |
| A_52_P31246 | NM_013883 | 29871 | *Scmh1* |  |  |  |  |  | 1.64 |
| A_52_P313861 |  |  |  |  | -1.95 |  |  |  |  |
| A_52_P313905 | NM_134122 | 106582 | *Nrm* | -1.52 |  |  |  |  |  |
| A_52_P314705 | NM_010931 | 18140 | *Uhrf1* |  |  |  | -1.74 |  |  |
| A_52_P31543 | NM_007570 | 12227 | *Btg2* |  | 1.55 | 1.78 |  |  |  |
| A_52_P319161 | NM_019641 | 16765 | *Stmn1* |  |  |  |  | -1.54 |  |
| A_52_P322181 | NM_007419 | 11554 | *Adrb1* |  |  |  |  | 1.76 |  |
| A_52_P326548 | NM_175276 | 225288 | *Fhod3* |  |  |  | 1.76 |  | 1.92 |
| A_52_P326664 | NM_199252 | 381058 | *Unc93a* |  |  | 1.67 |  |  |  |
| A_52_P327854 |  | 224432 | *Scaf4* | -1.74 |  |  |  |  |  |
| A_52_P328102 | XR_380545 | 320700 | *A930033H14Rik* |  |  | 1.58 |  |  |  |
| A_52_P32886 |  | 100503463 | *AI256396* | 1.78 |  |  |  |  |  |
| A_52_P33067 |  |  |  |  |  | 1.61 |  |  |  |
| A_52_P335354 | NM_001079876 | 237436 | *Gas2l3* |  | 1.51 |  |  |  |  |
| A_52_P342828 | XM_006510229 |  |  |  |  |  |  |  | 1.54 |
| A_52_P343617 | NM_008953 | 19194 | *Bpifa2* |  |  |  | 2.53 |  |  |
| A_52_P344152 |  |  |  |  |  |  | 1.65 |  |  |
| A_52_P344601 | NM_027220 | 69814 | *Prss32* |  |  |  |  |  | 2.08 |
| A_52_P348648 | NM_175456 | 223513 | *Abra* |  |  | 3.84 |  |  |  |
| A_52_P351116 | NM_010512 | 16000 | *Igf1* |  |  |  |  | -1.52 |  |
| A_52_P35217 | NM_007570 | 12227 | *Btg2* |  |  | 1.68 |  |  |  |
| A_52_P362772 |  |  |  | -1.60 |  |  |  |  |  |
| A_52_P363951 | NM_011580 | 21825 | *Thbs1* |  |  |  | 2.64 |  |  |
| A_52_P364776 | NM_008563 | 17215 | *Mcm3* | -1.52 |  |  |  |  |  |
| A_52_P366116 | NM_028233 | 72416 | *Lrpprc* |  |  |  |  | 1.63 |  |
| A_52_P366525 | NM_001039710 | 67876 | *Coq10b* |  | -1.54 |  |  |  |  |
| A_52_P371074 |  |  |  |  |  | -1.74 |  |  |  |
| A_52_P371918 | NM_028148 | 72193 | *Scaf11* |  |  |  | 1.57 |  |  |
| A_52_P373335 |  | 320709 | *Tmem117* |  | 1.81 |  |  |  |  |
| A_52_P374960 | NM_198112 | 239790 | *Ostn* |  |  | -2.03 |  | -1.79 | -2.25 |
| A_52_P375931 | NM_146215 | 234728 | *Cmtr2* |  |  |  | -1.53 |  |  |
| A_52_P37774 | NR_027966 | 67576 | *4930429B21Rik* |  |  | 1.51 |  |  |  |
| A_52_P380263 | NM_013723 | 27205 | *Podxl* |  |  |  |  |  | 1.67 |
| A_52_P384831 | NM_001166067 | 232156 | *Slc4a5* |  |  | 1.77 |  |  |  |
| A_52_P386558 |  | 77097 | *Tanc2* |  |  | 1.71 |  |  |  |
| A_52_P387458 |  | 380773 | *Slirp* |  |  |  |  | -1.70 |  |
| A_52_P389553 |  |  |  | 1.71 |  |  |  |  |  |
| A_52_P390700 |  |  |  |  |  | -1.55 |  |  |  |
| A_52_P392456 | NM_028810 | 74194 | *Rnd3* |  |  | 1.60 |  |  |  |
| A_52_P393314 | NM_011027 | 18439 | *P2rx7* |  |  |  | 1.53 |  |  |
| A_52_P39505 | NM_173864 | 317677 | *C1s2* |  | 1.87 |  |  |  |  |
| A_52_P40504 | XR_389234 | 102640779 | *LOC102640779* |  |  |  |  | 1.87 |  |
| A_52_P413646 | NM_007556 | 12161 | *Bmp6* |  |  |  |  |  | 1.59 |
| A_52_P413756 | NM_023734 | 74116 | *Pi16* |  |  |  |  |  | 1.58 |
| A_52_P420138 | NM_029332 | 75547 | *Akap13* |  | 1.56 |  |  |  |  |
| A_52_P421992 |  | 319603 | *A730009E18Rik* |  | 1.54 |  |  |  |  |
| A_52_P426605 |  | 57738 | *Slc15a2* |  | 1.96 |  |  |  |  |
| A_52_P430194 |  | 228608 | *Smox* |  | 1.56 |  |  |  |  |
| A_52_P431615 | NM_001277183 | 434223 | *Gm1966* |  |  |  |  | 1.67 |  |
| A_52_P432170 | XM_011249729 | 100040353 | *2810416G20Rik* |  |  |  |  | 1.53 |  |
| A_52_P43503 | NM_172503 | 212168 | *Zswim4* |  |  | 1.74 |  |  |  |
| A_52_P436282 |  |  |  | -1.72 |  |  |  |  |  |
| A_52_P437795 | NM_009866 | 12552 | *Cdh11* |  |  | -1.68 |  |  |  |
| A_52_P441949 | NM_025799 | 66848 | *Fuca2* |  |  |  |  | -1.70 |  |
| A_52_P441954 | NM_019935 | 18426 | *Ovol1* |  |  | 1.59 |  |  |  |
| A_52_P442126 | NM_001081220 | 217143 | *Gpr179* |  |  | 1.74 |  |  |  |
| A_52_P446724 |  |  |  |  |  |  |  | -1.71 |  |
| A_52_P447567 | XM_006501411 | 229722 | *5330417C22Rik* |  |  |  |  |  | 2.35 |
| A_52_P452689 | NM_007498 | 11910 | *Atf3* |  |  | 4.85 |  |  |  |
| A_52_P453374 | NM_001013019 | 68852 | *Lrrn4cl* |  |  |  |  |  | 1.78 |
| A_52_P457028 |  |  |  |  |  |  |  |  | 1.81 |
| A_52_P459046 |  | 67760 | *Slc38a2* |  |  |  | 1.59 |  |  |
| A_52_P460537 | NM_020028 | 53978 | *Lpar2* |  | -1.56 |  |  |  |  |
| A_52_P474551 | NR_001461 | 63830 | *Kcnq1ot1* |  | 1.59 |  |  |  |  |
| A_52_P474949 | NM_007690 | 12648 | *Chd1* |  | -1.50 |  |  |  |  |
| A_52_P476029 | NM_001001986 | 329540 | *Nol4l* |  |  |  |  |  | 1.57 |
| A_52_P48218 | NM_001002241 | 83561 | *Tdrd1* |  |  |  |  |  | -1.72 |
| A_52_P482897 | NM_009704 | 11839 | *Areg* |  |  | 1.69 |  |  |  |
| A_52_P483983 |  |  |  |  |  |  | 2.72 |  |  |
| A_52_P484903 | NM_008037 | 14284 | *Fosl2* |  |  |  |  | -1.61 | -1.58 |
| A_52_P485573 | NM_153484 | 21685 | *Tef* |  |  |  |  | 2.01 |  |
| A_52_P486063 |  |  |  |  |  | 1.69 |  |  |  |
| A_52_P486260 | NM_054077 | 116847 | *Prelp* |  | 1.54 |  | 1.84 |  | 1.81 |
| A_52_P489295 | NM_009621 | 11504 | *Adamts1* | -1.53 |  |  |  |  |  |
| A_52_P494622 | NM_013613 | 18227 | *Nr4a2* |  |  | 2.12 |  |  |  |
| A_52_P496142 | NM_009932 | 12827 | *Col4a2* |  |  |  | 1.69 |  |  |
| A_52_P509752 | NM_011235 | 19364 | *Rad51d* |  | 1.66 |  |  |  |  |
| A_52_P509853 | XM_006523087 |  |  |  |  | 1.63 |  |  |  |
| A_52_P512301 | NM_019479 | 55927 | *Hes6* |  |  | -1.87 |  |  |  |
| A_52_P514391 | NM_009829 | 12444 | *Ccnd2* |  |  |  |  | 1.50 |  |
| A_52_P515959 |  | 231991 | *Creb5* |  |  | 1.59 |  |  |  |
| A_52_P518827 | NM_009931 | 12826 | *Col4a1* |  |  |  |  |  | 1.58 |
| A_52_P525183 | NM_134188 | 171210 | *Acot2* |  |  | 1.64 |  |  |  |
| A_52_P527800 | NM_145158 | 246707 | *Emilin2* |  |  |  | 1.60 |  | 1.74 |
| A_52_P527977 | XM_006533253 |  |  |  | 1.54 |  |  |  |  |
| A_52_P52803 | NM_010478 | 15511 | *Hspa1b* |  |  | 2.28 |  |  |  |
| A_52_P529360 | NM_001003955 | 52055 | *Rab11fip5* |  |  |  | 1.54 |  | 1.53 |
| A_52_P533585 |  |  |  |  |  |  |  | -2.21 |  |
| A_52_P53596 | NM_001013370 | 140742 | *Sesn1* |  |  | -1.52 |  |  |  |
| A_52_P536434 | NM_184052 | 16000 | *Igf1* |  |  |  |  | -1.85 |  |
| A_52_P536735 | NM_016677 | 53602 | *Hpcal1* |  |  |  |  |  | 1.59 |
| A_52_P540219 | NM_011594 | 21858 | *Timp2* |  |  |  | 1.54 |  |  |
| A_52_P5454 | NM_054042 | 70445 | *Cd248* |  |  |  |  |  | 1.53 |
| A_52_P549184 | NM_178712 | 237175 | *Adgrg2* |  |  | 1.51 |  |  |  |
| A_52_P552955 | NM_010081 | 13518 | *Dst* |  |  |  |  | 1.55 |  |
| A_52_P556448 | NM_010667 | 16679 | *Krt86* |  |  |  | 2.23 |  |  |
| A_52_P558401 | NM_172301 | 268697 | *Ccnb1* |  |  |  |  | -1.74 |  |
| A_52_P577624 | NM_138664 | 246738 | *Dnajc28* |  | -2.40 |  |  |  |  |
| A_52_P578922 | NM_008255 | 15357 | *Hmgcr* |  |  |  | -1.57 |  |  |
| A_52_P58066 | XR_374791 | 102634512 | *Gm13791* | 1.74 |  |  |  |  |  |
| A_52_P583050 | NM_181649 | 53951 | *Gpatch11* |  |  |  |  | -2.39 |  |
| A_52_P590781 | NM_024244 | 71721 | *Fam13c* |  |  | -1.53 |  |  |  |
| A_52_P592466 | NM_023275 | 80837 | *Rhoj* |  | 1.62 |  |  |  |  |
| A_52_P593318 |  |  |  | 1.55 |  |  |  |  |  |
| A_52_P5945 | NM_008904 | 19017 | *Ppargc1a* |  |  |  | 1.97 |  |  |
| A_52_P59784 |  |  |  |  |  |  | 1.51 |  |  |
| A_52_P598732 |  |  |  |  |  | 2.08 |  |  |  |
| A_52_P604629 | NM_153287 | 215418 | *Csrnp1* | -1.70 |  |  |  |  |  |
| A_52_P607507 | NM_001030294 | 380924 | *Olfm4* |  |  |  | 3.59 |  | 3.67 |
| A_52_P609120 | NM_020271 | 57028 | *Pdxp* |  | -1.58 |  |  |  |  |
| A_52_P609972 | NM_007925 | 13717 | *Eln* |  |  |  | 1.52 |  |  |
| A_52_P624155 |  |  |  |  |  |  | -2.50 | -1.82 |  |
| A_52_P624969 |  |  |  |  | -1.67 |  |  |  |  |
| A_52_P63343 | NM_001033302 | 229599 | *Ciart* |  |  |  |  | 1.98 |  |
| A_52_P633979 |  |  |  |  | 1.90 |  |  |  |  |
| A_52_P637172 | NM_029011 | 74580 | *Pyroxd2* | 1.67 |  |  |  |  | 2.18 |
| A_52_P63728 | NM_011859 | 23967 | *Osr1* |  | 1.59 |  | 1.55 |  |  |
| A_52_P639461 |  |  |  |  |  |  | 2.10 |  |  |
| A_52_P642167 | NM_009230 | 20652 | *Soat1* |  | 1.60 |  |  |  |  |
| A_52_P644005 |  | 20680 | *Sox7* | 1.54 | 1.65 |  |  |  |  |
| A_52_P646762 | NM_022315 | 64074 | *Smoc2* |  |  |  |  |  | 1.52 |
| A_52_P648524 | NM_172119 | 107585 | *Dio3* |  |  | 1.74 | 1.77 |  |  |
| A_52_P650453 | NM_011463 | 20731 | *Spink4* | 2.79 |  |  |  |  |  |
| A_52_P650553 | NM_001039710 | 67876 | *Coq10b* | -1.63 | -1.81 |  |  |  |  |
| A_52_P651948 | NM_183254 | 66337 | *Fam229b* |  |  |  |  | 1.57 |  |
| A_52_P657883 | NM_027494 | 70650 | *Zcchc8* |  |  |  |  |  | -2.08 |
| A_52_P663303 | NM_146039 | 217935 | *Wdr60* |  | 1.61 |  |  |  |  |
| A_52_P665675 | NM_013454 | 11303 | *Abca1* |  |  |  | 1.53 |  |  |
| A_52_P671132 | NM_152915 | 227325 | *Dner* |  |  | -1.86 |  |  |  |
| A_52_P67892 |  |  |  |  |  | 1.63 |  |  |  |
| A_52_P679152 | NM_001042634 | 12747 | *Clk1* |  |  |  |  | -1.52 |  |
| A_52_P680827 | NM_172961 | 268860 | *Abat* | 1.56 |  |  |  |  | 1.81 |
| A_52_P681771 | NM_025802 | 66853 | *Pnpla2* |  |  |  | 1.58 |  |  |
| A_52_P682456 |  |  |  |  | -1.61 |  |  |  |  |
| A_52_P706621 | NM_001081229 | 72033 | *Tsc22d2* |  |  |  |  | -1.64 |  |
| A_52_P715191 |  |  |  |  |  | 1.61 |  |  |  |
| A_52_P739682 |  |  |  |  |  | 1.56 |  |  |  |
| A_52_P764117 | XM_006522462 | 665119 | *Sec14l5* |  |  |  |  |  | 2.14 |
| A_52_P793384 | NR_033324 | 436230 | *BC065397* |  |  | -1.55 |  |  |  |
| A_52_P79889 | NM_011065 | 18626 | *Per1* | -3.47 | -3.04 |  |  | -2.58 |  |
| A_52_P80305 |  |  |  |  |  |  | -1.51 |  |  |
| A_52_P811736 |  |  |  | 1.99 |  |  |  |  |  |
| A_52_P82741 | NM_010479 | 193740 | *Hspa1a* |  |  | 2.75 |  |  |  |
| A_52_P828218 | NM_001030294 | 380924 | *Olfm4* |  |  |  | 4.32 |  | 3.82 |
| A_52_P853177 | NM_011923 | 26360 | *Angptl2* |  |  |  |  |  | 1.57 |
| A_52_P85495 |  | 100049162 | *BC001981* |  |  |  |  |  | -1.59 |
| A_52_P868419 | NM_026346 | 67731 | *Fbxo32* |  |  | 1.68 |  |  |  |
| A_52_P878314 | XR_867748 | 102632865 | *Gm30827* |  |  | 1.55 |  |  |  |
| A_52_P88033 | NM_080728 | 140781 | *Myh7* |  |  |  |  |  | 2.80 |
| A_52_P8863 |  |  |  |  | -1.57 |  |  |  |  |
| A_52_P88878 | NM_020043 | 56741 | *Igdcc4* |  |  |  |  |  | 1.78 |
| A_52_P89273 |  |  |  |  | -1.66 |  | -1.61 |  |  |
| A_52_P915194 |  |  |  | -2.10 | -1.76 |  |  | -1.88 |  |
| A_52_P92398 | NM_001008502 | 241950 | *Bbs12* |  |  |  |  | 1.53 |  |
| A_52_P925197 | NM_007570 | 12227 | *Btg2* |  | 1.51 | 1.62 |  |  |  |
| A_52_P925277 | NM_001079883 | 58208 | *Bcl11b* | -1.54 | -1.64 |  | -1.52 |  |  |
| A_52_P932813 | XM_006504241 | 102634072 | *Gm38457* |  |  | 1.63 |  | 1.72 |  |
| A_52_P94830 | NM_001081421 | 108760 | *Galnt16* |  |  |  | 1.51 |  | 1.55 |
| A_52_P956261 | XM_006506528 | 68169 | *Ndnf* |  |  | -1.54 |  |  |  |
| A_52_P971150 | XM_011239233 | 105244150 | *LOC105244150* |  |  | 2.02 |  |  |  |
| A_52_P99082 |  |  |  |  |  |  |  | 1.90 |  |
| A_52_P996384 |  |  |  |  |  | 1.81 |  |  |  |
| A_52_P997209 |  |  |  |  |  | 1.92 |  |  |  |
| **(D) Mouse Palate Day 90** | | | | | | | | | |
| A_51_P101347 | NM_001033210 | 102502 | *Pls1* | -1.75 |  |  |  |  |  |
| A_51_P102471 | NM_021319 | 57757 | *Pglyrp2* |  | 1.77 |  |  |  |  |
| A_51_P103364 |  |  |  | 2.23 |  |  |  |  |  |
| A_51_P108767 | NM_009015 | 19366 | *Rad54l* |  |  |  |  | -1.55 |  |
| A_51_P109050 | NM_023824 | 76498 | *Paqr4* |  |  | -1.59 |  |  |  |
| A_51_P110640 | NM_010640 | 16613 | *Klk1b11* | -1.69 |  | -1.70 |  |  |  |
| A_51_P110689 |  | 20135 | *Rrm2* |  |  |  |  |  | -1.57 |
| A_51_P111492 | NM_017373 | 18030 | *Nfil3* |  |  |  |  |  | -1.53 |
| A_51_P115005 | NM_010104 | 13614 | *Edn1* |  |  | -1.57 |  |  |  |
| A_51_P116298 | NM_183161 | 228993 | *Slc17a9* | -1.88 | -1.77 | -1.93 |  |  |  |
| A_51_P117477 | NM_011977 | 26457 | *Slc27a1* |  |  |  |  | -1.69 |  |
| A_51_P124550 | NM_139292 | 70335 | *Reep6* | -1.70 |  |  |  |  |  |
| A_51_P125135 | NM_026410 | 67849 | *Cdca5* |  |  |  |  |  | -1.51 |
| A_51_P131561 | NM_029639 | 76509 | *Plet1* | -1.78 | -1.65 | -1.68 |  |  |  |
| A_51_P133005 | NM_134086 | 105727 | *Slc38a1* |  |  | -1.92 |  |  |  |
| A_51_P135012 | NM_027897 | 52428 | *Rhpn2* | -1.73 | -1.68 |  |  |  |  |
| A_51_P135092 | NM_134471 | 73804 | *Kif2c* |  |  | 1.53 |  |  |  |
| A_51_P137018 | XM_001477470 | 73988 | *4930438A08Rik* |  | 1.77 |  |  |  |  |
| A_51_P137433 | NM_001195298 | 100502766 | *Kifc1* |  |  | 2.44 |  |  |  |
| A_51_P140576 | NM_001038609 | 17762 | *Mapt* |  |  | -2.26 |  |  |  |
| A_51_P143162 | NM_080728 | 140781 | *Myh7* | -1.93 |  | -2.41 |  |  |  |
| A_51_P148756 | XM_011243484 | 327799 | *Usp44* |  |  |  |  | 1.71 |  |
| A_51_P149469 | NM_009608 | 11464 | *Actc1* |  |  | -1.61 |  |  |  |
| A_51_P150912 | NM_011497 | 20878 | *Aurka* |  | 1.69 |  |  |  |  |
| A_51_P153683 | NM_146117 | 227618 | *Lrrc26* | -2.07 | -1.72 | -1.83 |  |  |  |
| A_51_P154596 | NM_008721 | 18146 | *Npdc1* | -1.58 |  | -1.51 |  |  |  |
| A_51_P156222 | NM_175522 | 243312 | *Elfn1* | -1.56 |  | -1.61 |  |  |  |
| A_51_P157840 | NM_023850 | 76969 | *Chst1* |  |  |  |  | 1.52 |  |
| A_51_P161086 | NM_008908 | 19038 | *Ppic* | -1.64 |  | -1.75 |  |  |  |
| A_51_P164014 | NM_173762 | 229841 | *Cenpe* |  | 1.50 | 1.57 |  |  |  |
| A_51_P167313 | NM_171824 | 209966 | *Pgbd5* | 1.59 |  |  |  |  |  |
| A_51_P171180 |  |  |  |  |  |  |  |  | 1.53 |
| A_51_P176086 | NM_146187 | 233079 | *Ffar2* | -2.12 |  |  |  |  |  |
| A_51_P180492 | NM_016974 | 13170 | *Dbp* |  |  |  |  | 2.91 | 3.07 |
| A_51_P183543 |  |  |  |  |  |  |  |  | 1.53 |
| A_51_P183561 | NM_145473 | 105859 | *Csdc2* | -2.25 |  | -1.94 |  |  |  |
| A_51_P185882 | NM_201362 | 381175 | *Ccdc68* | -1.50 |  |  |  |  |  |
| A_51_P185897 |  |  |  |  |  | -2.33 |  |  |  |
| A_51_P186552 | NM_175454 | 217310 | *Hid1* | -1.87 |  | -1.86 |  |  |  |
| A_51_P188911 | NM_172693 | 230145 | *Galnt12* | -2.15 |  | -1.93 |  |  |  |
| A_51_P196657 |  |  |  |  |  |  | 1.62 |  |  |
| A_51_P197645 | NM_001291001 | 70426 | *Tekt5* |  |  |  |  | 1.52 |  |
| A_51_P200667 | NM_053155 | 94040 | *Clmn* | -1.58 | -1.66 | -1.83 |  |  |  |
| A_51_P204966 | NM_010671 | 16699 | *Krtap13* |  |  |  |  | 2.44 |  |
| A_51_P206165 | NR_027961 | 231253 | *9130230L23Rik* | -1.82 |  | -1.66 |  |  |  |
| A_51_P212592 | NM_173749 | 210622 | *Pamr1* | -1.81 |  | -2.03 |  |  |  |
| A_51_P219542 | NM_019425 | 54342 | *Gnpnat1* |  |  | -1.53 |  |  |  |
| A_51_P219722 | NM_025990 | 67135 | *Bpifa5* |  | 1.69 |  |  |  |  |
| A_51_P221998 | NM_207237 | 230815 | *Man1c1* | -1.74 |  |  |  |  |  |
| A_51_P223776 | NM_145434 | 217166 | *Nr1d1* |  |  |  | 1.81 | 2.72 | 2.56 |
| A_51_P225827 | NM_019935 | 18426 | *Ovol1* |  |  | 1.50 |  |  |  |
| A_51_P228295 | NM_001001880 | 68481 | *Mpzl1* |  | -1.58 |  |  |  |  |
| A_51_P229675 | NM_011636 | 22038 | *Plscr1* |  | -1.81 |  |  |  |  |
| A_51_P229676 | NM_011636 | 22038 | *Plscr1* |  | -1.69 |  |  |  |  |
| A_51_P229875 | XM_006506138 | 243780 | *E330009J07Rik* |  |  | -1.69 |  |  |  |
| A_51_P231499 | NM_008813 | 18605 | *Enpp1* |  |  | -1.51 |  |  |  |
| A_51_P232393 | NM_001081322 | 208943 | *Myo5c* | -1.68 | -1.66 | -2.00 |  |  |  |
| A_51_P232771 | NM_025745 | 66753 | *Erlec1* | -1.60 |  | -1.62 |  |  |  |
| A_51_P237668 | NM_009749 | 12069 | *Bex2* | -2.09 |  | -2.03 |  |  |  |
| A_51_P237688 | NM_133697 | 68552 | *Smim14* | -1.50 |  | -1.63 |  |  |  |
| A_51_P239984 | NM_012012 | 26909 | *Exo1* |  |  |  |  |  | -1.64 |
| A_51_P245412 | NM_008915 | 19057 | *Ppp3cc* |  |  |  |  | -1.51 |  |
| A_51_P245414 | NM_010639 | 16612 | *Klk1* |  |  | -1.76 |  |  |  |
| A_51_P248629 | NM_001037727 | 232201 | *Arhgap25* | 1.61 |  |  |  |  |  |
| A_51_P250058 | NM_010137 | 13819 | *Epas1* |  |  |  | -1.59 |  |  |
| A_51_P252157 | NM_011623 | 21973 | *Top2a* |  |  |  |  |  | -1.51 |
| A_51_P253642 | NM_010121 | 13666 | *Eif2ak3* | -1.72 |  | -1.72 |  |  |  |
| A_51_P255456 | NM_009994 | 13078 | *Cyp1b1* |  | 2.21 | 2.59 | 2.27 |  |  |
| A_51_P256665 | NM_173006 | 269823 | *Pon3* |  |  | -1.55 |  |  |  |
| A_51_P262871 | NM_053179 | 94181 | *Nans* |  |  | -1.62 |  |  |  |
| A_51_P263246 | NM_008748 | 18218 | *Dusp8* |  |  |  |  | -1.58 |  |
| A_51_P265026 | NM_010872 | 17948 | *Naip2* |  | -1.59 |  |  |  |  |
| A_51_P268234 | NM_029646 | 76527 | *Il34* |  | 1.58 |  |  |  |  |
| A_51_P270635 | NM_013528 | 14583 | *Gfpt1* | -2.03 | -2.06 | -2.18 |  |  |  |
| A_51_P274124 | NM_026345 | 67729 | *Mansc1* | -2.05 |  |  |  |  |  |
| A_51_P275623 | NM_015828 | 50798 | *Gne* | -1.70 |  |  |  |  |  |
| A_51_P276560 | NM_020279 | 56838 | *Ccl28* | -1.64 |  | -1.54 |  |  |  |
| A_51_P278653 | NM_023396 | 67874 | *Rprm* | -1.80 |  |  |  |  |  |
| A_51_P279693 | NM_009992 | 13076 | *Cyp1a1* |  | 2.32 | 2.10 | 2.08 | 2.10 |  |
| A_51_P280117 | NM_008812 | 18600 | *Padi2* |  |  | -1.79 |  |  |  |
| A_51_P281593 | NM_001293703 | 71910 | *Ppapdc1b* |  |  | -1.69 |  |  |  |
| A_51_P287093 | NM_172301 | 268697 | *Ccnb1* |  | 1.54 |  |  |  |  |
| A_51_P289862 | NM_001081155 | 110351 | *Rap1gap* | -1.83 |  |  |  |  |  |
| A_51_P290207 | NM_153526 | 231070 | *Insig1* |  |  |  |  |  | 1.73 |
| A_51_P291078 | NM_172710 | 231238 | *Sel1l3* | -2.20 |  | -2.05 |  |  |  |
| A_51_P292460 |  |  |  |  |  |  |  | -1.67 |  |
| A_51_P295206 | NM_178309 | 237911 | *Brip1* |  |  |  |  |  | -1.65 |
| A_51_P295896 | NM_028934 | 74430 | *4930452B06Rik* | -1.64 |  |  |  |  |  |
| A_51_P299107 |  | 56773 | *Chst5* | -2.27 |  | -2.30 |  |  |  |
| A_51_P303180 | NM_026667 | 68303 | *Fam114a1* | -1.74 |  | -1.57 |  |  |  |
| A_51_P307721 | NM_019626 | 12404 | *Cbln1* |  |  |  | -1.58 |  |  |
| A_51_P310164 | NM_001144992 | 72792 | *2810459M11Rik* | -1.87 |  |  |  |  |  |
| A_51_P311611 | NM_010045 | 13349 | *Ackr1* |  |  |  |  |  | -1.52 |
| A_51_P312673 | XM_006498048 | 241391 | *Galnt5* | -1.85 |  | -2.04 |  |  |  |
| A_51_P315682 | NM_183029 | 319765 | *Igf2bp2* | -1.79 |  |  |  |  |  |
| A_51_P318755 | NM_028930 | 74424 | *Tmc5* | -1.87 | -1.85 | -1.90 |  |  |  |
| A_51_P320281 | NM_010574 | 16372 | *Irx2* | -1.75 | -1.56 | -1.60 |  |  |  |
| A_51_P324814 | NM_010664 | 16668 | *Krt18* | -1.98 | -1.69 | -1.92 |  |  |  |
| A_51_P327511 | NM_008013 | 14190 | *Fgl2* | -1.76 | -1.67 |  |  |  |  |
| A_51_P328333 | NM_007634 | 12449 | *Ccnf* |  | 1.64 |  |  |  |  |
| A_51_P336056 | NM_013570 | 16671 | *Krt33b* |  |  | 1.53 |  |  |  |
| A_51_P337523 | NM_153534 | 210044 | *Adcy2* |  |  | -1.84 |  |  |  |
| A_51_P341041 | NM_183089 | 72107 | *Dscc1* |  |  |  |  |  | -1.71 |
| A_51_P346815 | NM_029541 | 76219 | *Arxes1* |  |  |  |  |  | 1.53 |
| A_51_P350817 | NM_009922 | 12797 | *Cnn1* | -2.07 |  | -2.52 |  |  |  |
| A_51_P351923 | NR_027827 | 80515 | *Chd3os* |  |  | -1.56 |  |  |  |
| A_51_P352782 | NM_011104 | 18754 | *Prkce* |  |  | -1.52 |  |  |  |
| A_51_P354913 | NM_027402 | 384061 | *Fndc5* |  |  | -2.43 |  |  |  |
| A_51_P357533 | NM_172614 | 224090 | *Tmem44* | -1.64 |  | -1.70 |  |  |  |
| A_51_P359262 | NM_025468 | 66286 | *Sec11c* |  |  | -1.58 |  |  |  |
| A_51_P361022 | NM_023223 | 107995 | *Cdc20* |  |  | 1.52 |  |  |  |
| A_51_P361678 | NM_026599 | 68178 | *Cgnl1* | -1.86 |  | -1.89 |  |  |  |
| A_51_P362969 | NM_025445 | 66251 | *Arfgap3* | -1.57 |  |  |  |  |  |
| A_51_P362973 | NM_025445 | 66251 | *Arfgap3* | -1.51 | -1.64 | -1.68 |  |  |  |
| A_51_P363187 | NM_008176 | 14825 | *Cxcl1* |  |  |  |  | -2.08 |  |
| A_51_P366542 | NM_011925 | 26364 | *Adgre5* |  | -1.51 |  |  |  |  |
| A_51_P367263 | NM_199222 | 235416 | *Lman1l* | -2.08 |  | -2.49 |  |  |  |
| A_51_P367866 | NM_007913 | 13653 | *Egr1* |  |  |  | 1.95 |  |  |
| A_51_P372550 | NM_026770 | 68567 | *Cgref1* | -2.52 |  |  |  |  |  |
| A_51_P374707 | NM_173007 | 269831 | *Tspan12* |  |  | -1.89 |  |  |  |
| A_51_P376789 | NM_145387 | 212974 | *Athl1* | -1.53 |  |  |  |  |  |
| A_51_P376934 | NM_019517 | 56175 | *Bace2* | -1.86 |  | -1.94 |  |  |  |
| A_51_P379997 |  | 14425 | *Galnt3* |  |  | -1.70 |  |  |  |
| A_51_P380005 | NM_015736 | 14425 | *Galnt3* | -1.66 |  | -1.74 |  |  |  |
| A_51_P380078 | NM_001122603 | 215384 | *Fcgbp* | -2.10 |  | -1.63 |  |  |  |
| A_51_P382912 | NM_130451 | 170441 | *Slc2a10* | -1.54 |  |  |  |  |  |
| A_51_P383194 | NM_008804 | 18585 | *Pde9a* | -1.52 |  |  |  |  |  |
| A_51_P383489 | NM_021891 | 60530 | *Fignl1* |  |  |  |  |  | -1.56 |
| A_51_P386810 | NM_177910 | 331026 | *Gmppb* | -1.57 |  |  |  |  |  |
| A_51_P391805 | NM_015789 | 50722 | *Dkkl1* |  | 1.51 |  |  |  |  |
| A_51_P393310 | NM_198034 | 320007 | *Sidt1* | -1.72 |  |  |  |  |  |
| A_51_P395309 |  |  |  | -2.08 |  | -2.36 |  |  |  |
| A_51_P397296 | NM_028584 | 73608 | *Marveld3* | -1.60 |  |  |  |  |  |
| A_51_P400269 | NM_172479 | 209837 | *Slc38a5* | -2.48 |  | -2.23 |  |  |  |
| A_51_P407323 | NM_007976 | 14067 | *F5* | -2.13 |  | -2.18 |  |  |  |
| A_51_P409311 | NM_199303 | 228796 | *Bpifb6* | -2.48 |  | -2.40 |  |  |  |
| A_51_P409408 | NM_010921 | 18095 | *Nkx3-1* | -2.61 |  |  |  |  |  |
| A_51_P410260 | NM_008846 | 18719 | *Pip5k1b* | -1.57 |  |  |  |  |  |
| A_51_P414072 | NM_008935 | 19126 | *Prom1* |  | -1.97 |  |  |  |  |
| A_51_P414243 | NM_153540 | 215494 | *Pomgnt2* |  |  | -1.57 |  |  |  |
| A_51_P415395 | NM_001081314 | 75697 | *C2cd4b* |  |  |  |  | -2.66 | -2.35 |
| A_51_P421790 | NM_013891 | 30051 | *Spdef* |  |  | -2.21 |  |  |  |
| A_51_P424338 | NM_008706 | 18104 | *Nqo1* |  | 1.51 |  |  |  |  |
| A_51_P427505 | NM_025673 | 66629 | *Golph3* | -1.60 |  | -1.51 |  |  |  |
| A_51_P428056 |  |  |  | 1.76 |  |  |  |  |  |
| A_51_P433192 | NM_029815 | 76960 | *Bcas1* | -2.03 |  | -2.04 |  |  |  |
| A_51_P433194 | NM_029815 | 76960 | *Bcas1* | -2.32 |  | -2.42 |  |  |  |
| A_51_P434758 | NM_001033170 | 73813 | *Fam83e* | -2.19 |  |  |  |  |  |
| A_51_P435588 | NM_011470 | 20758 | *Sprr2d* |  |  |  | -1.79 | -1.71 |  |
| A_51_P441469 | NM_021310 | 57748 | *Jmy* |  |  |  |  |  | 1.74 |
| A_51_P442889 |  |  |  | 1.76 |  |  |  |  |  |
| A_51_P443723 | NM_211358 | 228368 | *Slc35c1* |  |  | -1.54 |  |  |  |
| A_51_P444015 |  |  |  |  |  |  |  | 1.64 |  |
| A_51_P451151 | NM_026785 | 68612 | *Ube2c* |  |  |  |  |  | -1.73 |
| A_51_P451186 | NM_001003950 | 216363 | *Rab3ip* |  |  | -1.56 |  |  |  |
| A_51_P453475 | NM_009201 | 20514 | *Slc1a5* |  | -1.57 |  |  |  |  |
| A_51_P455647 | NM_009801 | 12349 | *Car2* | -1.69 |  | -1.72 |  |  |  |
| A_51_P456465 | NM_021386 | 58187 | *Cldn10* | -2.72 |  | -2.47 |  |  |  |
| A_51_P462533 | NM_173068 | 54525 | *Syt7* |  |  | -2.05 |  |  |  |
| A_51_P463816 | NM_001081426 | 208440 | *Dip2c* |  |  |  | -1.50 |  |  |
| A_51_P465292 | NM_080462 | 140483 | *Hnmt* | -1.61 |  | -1.61 |  |  |  |
| A_51_P468876 | NM_178118 | 330938 | *Dixdc1* |  | -1.98 |  |  |  |  |
| A_51_P469449 | NM_001040691 | 22256 | *Ung* |  |  | -1.80 |  |  |  |
| A_51_P469550 | NM_172523 | 214084 | *Slc18a2* |  | 1.56 |  |  |  |  |
| A_51_P472217 | NM_001081085 | 72080 | *Sapcd2* |  | 1.51 | 1.58 |  |  |  |
| A_51_P475948 |  | 234683 | *Elmo3* | 1.58 |  |  |  |  |  |
| A_51_P476618 | NM_134251 | 171286 | *Slc12a8* | -2.16 |  |  |  |  |  |
| A_51_P479321 | NM_080575 | 68738 | *Acss1* |  |  | -1.68 |  |  |  |
| A_51_P480602 | NM_009363 | 21785 | *Tff2* | -1.64 |  |  |  |  |  |
| A_51_P482121 | NM_007902 | 13615 | *Edn2* |  |  | 1.51 |  | 1.58 |  |
| A_51_P482503 | NM_009413 | 21987 | *Tpd52l1* |  |  | -1.97 |  |  |  |
| A_51_P483544 | NM_013930 | 30956 | *Aass* | -1.95 |  | -2.03 |  |  |  |
| A_51_P483617 | NM_029554 | 76261 | *0610040J01Rik* | -2.24 |  | -2.11 |  |  |  |
| A_51_P484289 | NM_178798 | 330836 | *Slc7a6* |  |  |  |  |  | -1.59 |
| A_51_P487178 | NM_172522 | 214058 | *Megf11* |  |  |  |  | -1.76 |  |
| A_51_P487973 | NM_001167883 | 99696 | *Ankrd50* |  |  |  |  | -1.61 |  |
| A_51_P487999 | NM_028232 | 72415 | *Sgol1* |  | 1.52 |  |  |  |  |
| A_51_P488538 | NM_054100 | 117172 | *2310034C09Rik* |  |  |  |  | 2.28 |  |
| A_51_P489788 | NM_134086 | 105727 | *Slc38a1* |  |  | -1.85 |  |  |  |
| A_51_P491667 | NM_024440 | 70377 | *Derl3* |  |  | -1.68 |  |  |  |
| A_51_P492047 | NM_020622 | 52793 | *Fam3b* | -1.72 |  |  |  |  |  |
| A_51_P499233 | NM_172616 | 224171 | *C330027C09Rik* |  | 1.50 |  |  |  |  |
| A_51_P501550 | XM_006496227 |  |  |  |  |  | 1.62 |  |  |
| A_51_P502437 | NM_009785 | 12294 | *Cacna2d3* | 1.54 |  |  |  |  |  |
| A_51_P508959 | NM_030024 | 78004 | *Prr15* | -1.62 |  |  |  |  |  |
| A_51_P514139 | NM_027504 | 70673 | *Prdm16* |  |  |  |  | 1.88 |  |
| A_51_P514421 | NM_011895 | 24060 | *Slc35a1* | -1.79 |  | -1.70 |  |  |  |
| A_51_P517430 | NM_007639 | 12479 | *Cd1d1* |  |  | -1.69 |  |  |  |
| A_51_P519791 | NM_198605 | 219114 | *Ska3* |  | 1.53 |  |  |  |  |
| A_52_P1037106 |  |  |  |  | 1.64 |  |  |  |  |
| A_52_P1068320 | NM_009399 | 21934 | *Tnfrsf11a* | -1.52 |  |  |  |  |  |
| A_52_P1084681 | NM_001048175 | 13522 | *Adam28* |  |  | -1.80 |  |  |  |
| A_52_P1092823 | NM_010573 | 16371 | *Irx1* | -1.92 |  |  |  |  |  |
| A_52_P111715 | NM_172451 | 207839 | *Galnt6* | -2.24 |  | -2.13 |  |  |  |
| A_52_P118161 | NM_146006 | 16987 | *Lss* |  |  |  |  |  | 1.53 |
| A_52_P120228 |  |  |  |  |  | -1.71 |  |  |  |
| A_52_P12023 |  |  |  |  |  |  |  |  | -1.53 |
| A_52_P121502 | NM_026385 | 67801 | *Pllp* | -1.81 |  | -1.71 |  |  |  |
| A_52_P129867 | NM_177244 | 320720 | *Fastkd1* |  |  | -1.52 |  |  |  |
| A_52_P130079 | NM_001081150 | 244421 | *Lonrf1* |  |  | -1.50 |  |  |  |
| A_52_P130490 | NM_001177544 | 665433 | *Hist1h2ao* |  |  |  |  |  | -1.61 |
| A_52_P134195 | NM_007675 | 26366 | *Ceacam10* | -2.27 |  | -2.17 |  |  |  |
| A_52_P13448 | NM_026515 | 68026 | *2810417H13Rik* |  |  |  |  | -1.56 |  |
| A_52_P136709 | NM_008796 | 18559 | *Pctp* | -1.56 |  |  |  |  |  |
| A_52_P148553 | NM_021891 | 60530 | *Fignl1* |  |  |  |  |  | -1.52 |
| A_52_P149336 | NM_183046 | 240641 | *Kif20b* |  | 1.55 | 1.55 |  |  |  |
| A_52_P149705 | NM_177222 | 320662 | *Casc1* |  |  |  |  | -1.61 |  |
| A_52_P151393 | NM_198860 | 192734 | *Lrrc75b* | -2.03 |  | -1.88 |  |  |  |
| A_52_P151887 | XR_864896 |  |  | 2.33 |  |  |  |  |  |
| A_52_P154005 | NM_173026 | 271377 | *Zbtb11* | 1.51 |  |  |  |  |  |
| A_52_P15461 | NM_008357 | 16168 | *Il15* |  |  |  |  | 1.61 |  |
| A_52_P159470 | NM_207243 | 239611 | *Muc19* | -2.48 |  | -2.50 |  |  |  |
| A_52_P173703 | NM_144557 | 245049 | *Myrip* | -1.68 |  | -1.77 |  |  |  |
| A_52_P175376 | NM_023755 | 81879 | *Tfcp2l1* |  | -1.60 | -1.59 |  |  |  |
| A_52_P176659 |  |  |  | -1.77 |  | -1.81 |  |  |  |
| A_52_P183752 | NM_007754 | 12874 | *Cpd* | -1.90 |  |  |  |  |  |
| A_52_P188589 | NM_001256059 | 100503884 | *Ccdc149* | -1.61 |  | -1.65 |  |  |  |
| A_52_P189377 | NM_175367 | 108800 | *Ston2* |  |  | 1.63 |  |  |  |
| A_52_P193256 |  |  |  |  |  |  |  | 1.62 |  |
| A_52_P201482 |  | 243548 | *Prickle2* |  |  | 1.51 |  |  |  |
| A_52_P204331 | NM_178727 | 242484 | *D630039A03Rik* | -1.87 |  |  |  |  |  |
| A_52_P205813 | NM_144731 | 108150 | *Galnt7* | -1.70 | -1.67 | -1.82 |  |  |  |
| A_52_P210078 | NM_007392 | 11475 | *Acta2* |  |  | -2.06 |  |  |  |
| A_52_P214630 | NM_011448 | 20682 | *Sox9* | -1.71 |  | -1.91 |  |  |  |
| A_52_P215093 | NM_133779 | 78928 | *Pigt* | -1.52 |  |  |  |  |  |
| A_52_P226407 | XM_006504089 |  |  |  |  | -1.51 |  |  |  |
| A_52_P229316 | NM_015737 | 14426 | *Galnt4* | -2.10 |  | -2.14 |  |  |  |
| A_52_P230773 |  | 67112 | *Fgf22* |  |  | 1.51 |  |  |  |
| A_52_P237792 | NM_031196 | 20509 | *Slc19a1* |  | 1.74 |  |  |  |  |
| A_52_P239536 | NM_181595 | 243725 | *Ppp1r9a* |  |  | -1.68 |  |  |  |
| A_52_P241676 | NM_023449 | 65962 | *Slc9a3r2* |  |  | -1.62 |  |  |  |
| A_52_P246229 | NM_199222 | 235416 | *Lman1l* | -2.20 |  | -2.13 |  |  |  |
| A_52_P246252 | NM_007812 | 13087 | *Cyp2a5* |  |  |  | -1.81 |  |  |
| A_52_P24696 |  |  |  |  |  | -2.00 |  |  |  |
| A_52_P257026 |  |  |  |  |  |  |  |  | 1.69 |
| A_52_P257705 | NM_028672 | 73873 | *Fam161a* |  |  |  |  |  | 1.71 |
| A_52_P265556 |  |  |  |  |  |  |  |  | 1.64 |
| A_52_P278192 | NM_011232 | 19355 | *Rad1* |  |  |  |  | -1.51 |  |
| A_52_P289071 | NM_177054 | 319996 | *Casc4* | -1.87 |  |  |  |  |  |
| A_52_P290673 |  |  |  |  | -1.55 |  |  |  |  |
| A_52_P293482 | NM_019516 | 56072 | *Lgals12* |  |  | -1.58 |  |  |  |
| A_52_P297642 | NM_028295 | 72599 | *Pdia5* | -2.06 | -1.76 | -2.19 |  |  |  |
| A_52_P303891 | NM_011584 | 353187 | *Nr1d2* |  |  |  | 1.57 | 2.08 | 2.01 |
| A_52_P309084 | XM_006512692 | 215821 | *Arfgef3* | -1.58 |  |  |  |  |  |
| A_52_P314705 | NM_010931 | 18140 | *Uhrf1* |  |  |  |  | -1.64 |  |
| A_52_P316376 | NM_176850 | 207165 | *Bptf* | 1.59 |  |  |  |  |  |
| A_52_P317524 | NM_153165 | 236904 | *Klhl15* |  |  |  |  | 1.50 |  |
| A_52_P318073 |  |  |  |  | 1.53 |  |  |  |  |
| A_52_P319438 | NM_001039562 | 654824 | *Ankrd37* |  |  |  |  | 1.80 | 1.57 |
| A_52_P321150 | NM_023516 | 69573 | *Hilpda* | -1.54 |  |  |  |  |  |
| A_52_P326399 | NM_024169 | 66120 | *Fkbp11* | -2.18 |  | -1.95 |  |  |  |
| A_52_P335354 | NM_001079876 | 237436 | *Gas2l3* |  | 1.64 |  |  |  |  |
| A_52_P335590 |  | 66638 | *5730458M16Rik* | 1.70 |  |  |  |  |  |
| A_52_P335842 |  |  |  |  | -1.54 | -1.55 |  |  |  |
| A_52_P33800 | NM_207636 | 319448 | *Fndc3a* |  |  | -1.63 |  |  |  |
| A_52_P340669 | NM_010800 | 17341 | *Bhlha15* |  |  | -2.24 |  |  |  |
| A_52_P343627 | NM_022020 | 63954 | *Rbp7* |  | 1.54 |  |  |  |  |
| A_52_P344601 | NM_027220 | 69814 | *Prss32* | -1.66 |  |  |  |  |  |
| A_52_P357309 | NM_145527 | 228355 | *Madd* |  | 1.73 |  |  |  |  |
| A_52_P361113 |  |  |  |  |  |  |  | -1.51 |  |
| A_52_P369613 | NM_153541 | 215627 | *Zbtb8b* |  |  |  |  | -2.57 |  |
| A_52_P370268 | NM_009638 | 11571 | *Crisp1* |  |  | -1.86 |  |  |  |
| A_52_P37593 | NM_015828 | 50798 | *Gne* | -1.91 | -1.69 | -1.80 |  |  |  |
| A_52_P375970 | NM_001039485 | 667742 | *Piezo2* |  |  | -1.51 |  |  |  |
| A_52_P382149 | NM_007811 | 13082 | *Cyp26a1* |  |  |  | -1.51 |  |  |
| A_52_P382376 |  | 59048 | *C1galt1c1* |  | 1.65 |  |  |  |  |
| A_52_P393392 | NM_153526 | 231070 | *Insig1* |  |  |  |  |  | 1.54 |
| A_52_P396401 | NM_029548 | 380714 | *Rph3al* |  |  |  |  | -3.36 |  |
| A_52_P398122 | NM_010574 | 16372 | *Irx2* | -1.81 |  |  |  |  |  |
| A_52_P401535 | NM_130904 | 170779 | *Cd209d* |  |  |  |  | 1.53 |  |
| A_52_P402786 | NM_008935 | 19126 | *Prom1* | -1.75 | -1.84 | -1.73 |  |  |  |
| A_52_P413646 | NM_007556 | 12161 | *Bmp6* |  |  | -1.57 |  |  |  |
| A_52_P417694 | NM_027078 | 67454 | *Ikbip* |  |  | -1.53 |  |  |  |
| A_52_P419059 | NM_001122603 | 215384 | *Fcgbp* | -2.08 |  |  |  |  |  |
| A_52_P421234 | NM_133832 | 98711 | *Rdh10* |  |  | -1.54 |  |  |  |
| A_52_P423380 | NM_027870 | 71703 | *Armcx3* |  |  | -1.54 |  |  |  |
| A_52_P430462 | NM_134086 | 105727 | *Slc38a1* | -1.76 |  | -2.00 |  |  |  |
| A_52_P43456 |  |  |  |  | -1.59 |  |  |  |  |
| A_52_P439263 | NM_011674 | 22239 | *Ugt8a* | -1.56 |  |  |  |  |  |
| A_52_P440621 | NM_175279 | 78748 | *Rassf10* |  |  |  | -1.54 |  |  |
| A_52_P447944 | NM_008532 | 17075 | *Epcam* | -1.89 |  | -1.65 |  |  |  |
| A_52_P448253 | NM_020583 | 57444 | *Isg20* | -1.86 |  |  |  |  |  |
| A_52_P448749 | NM_028924 | 74413 | *Tc2n* |  |  | -2.13 |  |  |  |
| A_52_P451073 | NM_178589 | 94185 | *Tnfrsf21* | -1.51 |  |  |  |  |  |
| A_52_P457028 |  |  |  |  |  | -1.62 |  |  |  |
| A_52_P463271 | NM_146041 | 218138 | *Gmds* |  |  | -2.08 |  |  |  |
| A_52_P4666 | NM_021790 | 60411 | *Cenpk* |  |  |  |  |  | -1.57 |
| A_52_P469956 | NM_178404 | 78751 | *Zc3h6* |  |  |  |  | 1.63 | 1.54 |
| A_52_P470466 | NM_177664 | 229791 | *Lppr4* |  |  | -2.02 |  |  |  |
| A_52_P473966 | NM_134090 | 105785 | *Kdelr3* | -1.82 |  | -1.89 |  |  |  |
| A_52_P475271 |  | 74107 | *Cep55* |  | 1.73 |  |  |  |  |
| A_52_P476029 | NM_001001986 | 329540 | *Nol4l* |  |  | -1.52 |  |  |  |
| A_52_P48546 | NM_026259 | 67588 | *Rnf41* |  | 1.64 |  |  |  |  |
| A_52_P485573 | NM_153484 | 21685 | *Tef* |  |  |  |  | 2.04 | 2.05 |
| A_52_P489202 | XR_380952 | 414105 | *4732465J04Rik* |  |  | 1.59 |  |  |  |
| A_52_P490493 | NM_026599 | 68178 | *Cgnl1* | -1.95 |  |  |  |  |  |
| A_52_P498193 | NM_153543 | 216188 | *Aldh1l2* |  |  | -1.73 |  |  |  |
| A_52_P500027 | NM_017373 | 18030 | *Nfil3* |  |  |  |  |  | -1.58 |
| A_52_P502771 | NM_001039556 | 623474 | *Rad54b* | 1.56 |  |  |  |  | -1.63 |
| A_52_P503927 | NM_001033178 | 77106 | *Tmem181a* |  |  | -1.51 |  |  |  |
| A_52_P517683 | NM_011526 | 21345 | *Tagln* |  |  | -1.75 |  |  |  |
| A_52_P517762 | NM_023557 | 70129 | *Slc44a4* | -1.78 |  |  |  |  |  |
| A_52_P545643 | NM_001013028 | 103266 | *Tmem263* |  | -1.63 | -1.62 |  |  |  |
| A_52_P54812 | NM_001012434 | 233529 | *Kctd14* | -2.09 | -1.72 | -2.11 |  |  |  |
| A_52_P5491 | NM_172862 | 242022 | *Frem2* |  |  | -1.74 |  |  |  |
| A_52_P550734 | NM_053173 | 16580 | *Kifc5b* |  |  | 2.19 |  |  |  |
| A_52_P552879 | NM_001025103 | 213573 | *Cracr2b* | -1.73 |  |  |  |  |  |
| A_52_P557059 | NM_146520 | 258513 | *Olfr536* |  |  |  |  | 1.60 |  |
| A_52_P577484 | NM_011448 | 20682 | *Sox9* | -1.84 |  | -1.91 |  |  |  |
| A_52_P58524 | NM_010893 | 18010 | *Neu1* | -1.53 |  |  |  |  |  |
| A_52_P593379 | NM_011041 | 18511 | *Pax9* |  |  | -1.84 |  |  |  |
| A_52_P593965 |  | 110196 | *Fdps* |  |  | 1.54 |  |  | 1.54 |
| A_52_P59908 | XR_868243 | 102633802 | *Gm31539* | 1.79 |  |  |  |  |  |
| A_52_P600030 | NM_027188 | 69726 | *Smyd3* |  | 1.55 |  |  |  |  |
| A_52_P608255 | NM_138606 | 18715 | *Pim2* |  |  |  | 1.67 |  |  |
| A_52_P615225 | NM_007407 | 11517 | *Adcyap1r1* |  |  | -1.63 |  |  |  |
| A_52_P620497 |  | 319967 | *9630019E01Rik* |  |  |  |  |  | 1.50 |
| A_52_P624155 |  |  |  |  |  |  |  | -1.83 |  |
| A_52_P629333 | NM_145608 | 234878 | *BC021891* |  |  | -1.72 |  |  |  |
| A_52_P63336 | NM_024181 | 66861 | *Dnajc10* | -1.84 |  |  |  |  |  |
| A_52_P633941 | NM_021322 | 57773 | *Wdr4* |  |  |  |  |  | 1.53 |
| A_52_P642488 | NM_008430 | 16525 | *Kcnk1* | -1.67 |  |  |  |  |  |
| A_52_P644005 |  | 20680 | *Sox7* |  |  |  |  | 1.70 |  |
| A_52_P645675 |  |  |  |  |  |  |  |  | 2.02 |
| A_52_P652513 | NM_027400 | 70361 | *Lman1* |  |  | -1.64 |  |  |  |
| A_52_P654841 | NM_011636 | 22038 | *Plscr1* |  | -1.65 |  |  |  |  |
| A_52_P655803 | NM_144807 | 212862 | *Chpt1* |  |  | -1.79 |  |  |  |
| A_52_P662226 | NM_173006 | 269823 | *Pon3* |  |  | -1.63 |  |  |  |
| A_52_P670812 | NR_038186 | 100616095 | *Snhg18* | -1.53 |  |  |  |  |  |
| A_52_P676623 | NM_028319 | 72672 | *Zfp518a* |  |  | -1.51 |  |  |  |
| A_52_P679105 | NM_029614 | 76453 | *Prss23* |  |  | -1.59 |  |  |  |
| A_52_P681495 | NM_027930 | 71804 | *Mtfr2* |  | 1.53 |  |  |  | -1.51 |
| A_52_P69955 | NM_021344 | 57816 | *Tesc* | -1.81 |  | -1.70 |  |  |  |
| A_52_P7610 | NM_080440 | 110893 | *Slc8a3* |  |  | -1.68 |  |  |  |
| A_52_P76931 | NM_172598 | 218973 | *Wdhd1* |  |  |  |  | -1.54 |  |
| A_52_P78770 | NM_144807 | 212862 | *Chpt1* |  |  | -1.63 |  |  |  |
| A_52_P79889 | NM_011065 | 18626 | *Per1* |  |  |  |  |  | -1.57 |
| A_52_P87804 | NM_001114662 | 213006 | *Mfsd4* |  | -2.03 | -2.22 |  |  |  |
| A_52_P88033 | NM_080728 | 140781 | *Myh7* | -2.10 |  | -2.38 |  |  |  |
| A_52_P88878 | NM_020043 | 56741 | *Igdcc4* |  | -1.54 | -1.50 |  |  |  |
| A_52_P900467 |  | 105244195 | *LOC105244195* |  |  |  |  | 1.60 |  |
| A_52_P923110 | XM_006527616 | 236727 | *Slc9a7* |  | -1.79 |  |  |  |  |
| A_52_P931133 |  |  |  | 1.63 |  |  |  |  |  |
| A_52_P93837 | NM_008604 | 17380 | *Mme* |  |  | 1.58 |  |  |  |
| A_52_P988287 |  |  |  |  | -1.56 |  |  |  |  |

^1^ 5 ppm Cr(VI) was not evaluated in rats

**Supplementary Table IV. BMDExpress dose-response results for potential differentially expressed genes (pDEGs) with goodness-of-fit p > 0.1.** The following are reported with their respective winning models, benchmark dose (BMD) values, BMD lower confidence limits (BMDLs), BMD/BMDL ratios, and winning fit p-values for: (**A**) 20 pDEGs in the rat oral mucosa after 7 days of exposure, (**B**) 7 pDEGs in the rat oral mucosa after 90 days of exposure, (**C**) 36 pDEGs in the mouse oral mucosa after 7 days of exposure, and (**D**) 31 pDEGs in the mouse oral mucosa after 90 days of exposure.

| **Gene Symbol** | **Entrez**  **Gene ID** | **Winning Model** | **Winning BMD (mg/L SDD)** | **Winning BMDL**  **(mg/L SDD)** | **BMD/BMDL** | **Winning Fit**  **p-value** |
| --- | --- | --- | --- | --- | --- | --- |
| **(A) Rat Palate Day 8** | | | | | | |
| *Aacs* | 65984 | Polynomial 2° | 86.52 | 54.18 | 1.60 | 0.23 |
| *Arsj* | 311013 | Power | 508.49 | 355.57 | 1.43 | 0.65 |
| *Bglap* | 25295 | Linear (1°) | 352.58 | 228.24 | 1.54 | 0.25 |
| *Ccdc176* | 500693 | Linear (1°) | 283.13 | 194.44 | 1.46 | 0.71 |
| *Cxcl1* | 81503 | Linear (1°) | 317.16 | 211.57 | 1.50 | 0.21 |
| *Fam8a1* | 291031 | Polynomial 2° | 480.30 | 407.59 | 1.18 | 0.30 |
| *Fos* | 314322 | Linear (1°) | 278.51 | 192.02 | 1.45 | 0.22 |
| *Gfod1* | 306842 | Linear (1°) | 341.56 | 223.18 | 1.53 | 0.17 |
| *Gp2* | 171459 | Linear (1°) | 275.95 | 190.68 | 1.45 | 0.19 |
| *Il10ra* | 117539 | Linear (1°) | 185.35 | 138.22 | 1.34 | 0.74 |
| *Kdm4b* | 301128 | Linear (1°) | 331.63 | 218.52 | 1.52 | 0.74 |
| *Npepo* | 290963 | Linear (1°) | 236.20 | 168.84 | 1.40 | 0.63 |
| *Nr1d1* | 252917 | Hill | 6.15 | 2.10 | 2.93 | 0.74 |
| *Nr1d2* | 259241 | Polynomial 2° | 58.03 | 40.61 | 1.43 | 0.21 |
| *Rec114* | 300751 | Linear (1°) | 326.04 | 215.86 | 1.51 | 1.00 |
| *Syt1* | 25716 | Power | 490.85 | 255.13 | 1.92 | 0.28 |
| *Syt7* | 59267 | Polynomial 2° | 96.67 | 57.99 | 1.67 | 0.73 |
| *Tspan15* | 679462 | Polynomial 2° | 89.83 | 54.62 | 1.64 | 0.47 |
| *Vps13d* | 313825 | Hill | 52.20 | 11.76 | 4.44 | 0.90 |
| *Wisp1* | 65154 | Linear (1°) | 255.17 | 179.48 | 1.42 | 0.73 |
| **(B) Rat Palate Day 91** | | | | | | |
| *Arsj* | 311013 | Linear (1°) | 361.78 | 232.40 | 1.56 | 0.44 |
| *Gadl1* | 367181 | Polynomial 2° | 57.93 | 40.41 | 1.43 | 0.46 |
| *LOC103693841* | 103693841 | Polynomial 2° | 109.76 | 61.68 | 1.78 | 0.21 |
| *Nr1d1* | 252917 | Polynomial 2° | 62.07 | 42.89 | 1.45 | 0.89 |
| *Rasd1* | 64455 | Linear (1°) | 349.56 | 226.87 | 1.54 | 0.48 |
| *Spdya* | 192209 | Linear (1°) | 298.84 | 202.49 | 1.48 | 0.69 |
| *Srrm3* | 685890 | Polynomial 2° | 99.12 | 59.24 | 1.67 | 0.58 |
| **(C) Mouse Palate Day 8** | | | | | | |
| *1700125H20Rik* | 73634 | Linear (1°) | 342.20 | 225.61 | 1.52 | 0.10 |
| *Adgrf5* | 224792 | Polynomial 2° | 87.06 | 54.68 | 1.59 | 0.35 |
| *Adrb1* | 11554 | Polynomial 2° | 61.42 | 42.58 | 1.44 | 0.86 |
| *B3gnt8* | 232984 | Polynomial 2° | 55.56 | 39.45 | 1.41 | 0.99 |
| *Ccdc116* | 76872 | Polynomial 2° | 70.08 | 46.96 | 1.49 | 0.84 |
| *Cdyl* | 12593 | Power | 104.76 | 27.74 | 3.78 | 0.14 |
| *Cemip* | 80982 | Polynomial 2° | 43.52 | 32.47 | 1.34 | 0.75 |
| *Cemip* | 80982 | Polynomial 2° | 50.20 | 36.54 | 1.37 | 0.61 |
| *Creb3l1* | 26427 | Linear (1°) | 433.49 | 264.90 | 1.64 | 0.16 |
| *Dagla* | 269060 | Linear (1°) | 451.10 | 271.77 | 1.66 | 0.13 |
| *Dlk1* | 13386 | Polynomial 2° | 88.00 | 55.09 | 1.60 | 0.24 |
| *Dst* | 13518 | Polynomial 2° | 80.20 | 51.55 | 1.56 | 0.24 |
| *Dst* | 13518 | Polynomial 2° | 122.63 | 66.08 | 1.86 | 0.83 |
| *Dtna* | 13527 | Linear (1°) | 289.55 | 199.73 | 1.45 | 0.26 |
| *Eno2* | 13807 | Polynomial 2° | 118.65 | 65.68 | 1.81 | 0.54 |
| *Evi5l* | 213027 | Polynomial 2° | 88.94 | 55.36 | 1.61 | 0.31 |
| *Fbxo32* | 67731 | Hill | 0.92 | 0.14 | 6.45 | 0.23 |
| *Fuca2* | 66848 | Polynomial 2° | 136.08 | 70.74 | 1.92 | 0.70 |
| *Fzd4* | 14366 | Power | 50.84 | 4.98 | 10.21 | 0.71 |
| *Gal3st4* | 330217 | Polynomial 2° | 74.19 | 49.04 | 1.51 | 0.39 |
| *Hpcal1* | 53602 | Linear (1°) | 301.33 | 205.75 | 1.46 | 0.61 |
| *Il17d* | 239114 | Linear (1°) | 289.44 | 199.67 | 1.45 | 0.15 |
| *Me1* | 17436 | Linear (1°) | 266.41 | 187.48 | 1.42 | 0.34 |
| *Melk* | 17279 | Polynomial 2° | 57.15 | 40.51 | 1.41 | 0.34 |
| *Mpv17l* | 93734 | Linear (1°) | 429.42 | 263.28 | 1.63 | 0.50 |
| *Ncdn* | 26562 | Linear (1°) | 263.65 | 185.99 | 1.42 | 0.65 |
| *Nov* | 18133 | Power | 203.85 | 16.76 | 12.17 | 0.35 |
| *Nr1d1* | 217166 | Polynomial 2° | 59.97 | 41.86 | 1.43 | 0.46 |
| *Pik3ip1* | 216505 | Polynomial 2° | 49.95 | 36.25 | 1.38 | 0.10 |
| *Pnpla2* | 66853 | Hill | 6.42 | 1.30 | 4.94 | 0.44 |
| *Podxl* | 27205 | Power | 22.56 | 1.97 | 11.45 | 0.68 |
| *Prkg1* | 19091 | Linear (1°) | 303.54 | 206.86 | 1.47 | 0.77 |
| *Rin1* | 225870 | Polynomial 2° | 58.22 | 40.93 | 1.42 | 0.17 |
| *Rtn4* | 68585 | Hill | 4.07 | 0.42 | 9.75 | 0.61 |
| *Tfap4* | 83383 | Polynomial 2° | 83.36 | 52.92 | 1.58 | 0.11 |
| *Zfp106* | 20402 | Linear (1°) | 297.90 | 204.01 | 1.46 | 0.74 |
| **(D) Mouse Palate Day 91** | | | | | | |
| *2810417H13Rik* | 68026 | Polynomial 2° | 46.43 | 34.30 | 1.35 | 0.20 |
| *Ankrd50* | 99696 | Polynomial 2° | 165.80 | 75.69 | 2.19 | 0.57 |
| *Brip1* | 237911 | Linear (1°) | 274.74 | 191.96 | 1.43 | 0.17 |
| *C2cd4b* | 75697 | Polynomial 2° | 33.64 | 26.03 | 1.29 | 0.46 |
| *Cd209d* | 170779 | Polynomial 2° | 85.92 | 54.35 | 1.58 | 0.40 |
| *Cenpk* | 60411 | Linear (1°) | 224.00 | 163.52 | 1.37 | 0.96 |
| *Dbp* | 13170 | Hill | 71.38 | 51.42 | 1.39 | 0.47 |
| *Dscc1* | 72107 | Power | 67.95 | 26.04 | 2.61 | 0.19 |
| *Exo1* | 26909 | Polynomial 2° | 57.27 | 40.75 | 1.41 | 0.44 |
| *Fam161a* | 73873 | Linear (1°) | 442.97 | 268.62 | 1.65 | 0.17 |
| *Fignl1* | 60530 | Polynomial 2° | 79.59 | 52.09 | 1.53 | 0.41 |
| *Hist1h2ao* | 665433 | Polynomial 2° | 39.95 | 30.27 | 1.32 | 0.22 |
| *Il15* | 16168 | Polynomial 2° | 91.38 | 55.96 | 1.63 | 0.51 |
| *Jmy* | 57748 | Linear (1°) | 255.54 | 181.54 | 1.41 | 0.92 |
| *Klhl15* | 236904 | Polynomial 2° | 77.02 | 50.13 | 1.54 | 0.28 |
| *Lss* | 16987 | Linear (1°) | 219.48 | 160.85 | 1.36 | 0.55 |
| *Nfil3* | 18030 | Power | 27.89 | 3.78 | 7.39 | 0.28 |
| *Nfil3* | 18030 | Power | 6.30 | 0.56 | 11.24 | 0.59 |
| *Nr1d1* | 217166 | Power | 1.15 | 0.07 | 16.17 | 0.15 |
| *Nr1d2* | 353187 | Polynomial 2° | 42.12 | 31.64 | 1.33 | 0.39 |
| *Rad1* | 19355 | Polynomial 2° | 80.28 | 52.04 | 1.54 | 0.79 |
| *Rad54l* | 19366 | Polynomial 2° | 53.19 | 38.30 | 1.39 | 0.16 |
| *Slc7a6* | 330836 | Polynomial 2° | 501.17 | 423.29 | 1.18 | 0.12 |
| *Sox7* | 20680 | Polynomial 2° | 146.28 | 73.60 | 1.99 | 0.71 |
| *Tef* | 21685 | Polynomial 2° | 61.86 | 43.23 | 1.43 | 0.44 |
| *Tekt5* | 70426 | Polynomial 2° | 72.58 | 48.55 | 1.49 | 0.70 |
| *Uhrf1* | 18140 | Polynomial 2° | 58.76 | 41.37 | 1.42 | 0.31 |
| *Usp44* | 327799 | Polynomial 2° | 120.81 | 66.22 | 1.82 | 0.24 |
| *Wdhd1* | 218973 | Polynomial 2° | 65.88 | 45.21 | 1.46 | 0.81 |
| *Wdr4* | 57773 | Linear (1°) | 517.87 | 296.03 | 1.75 | 0.61 |
| *Zc3h6* | 78751 | Polynomial 2° | 119.12 | 67.66 | 1.76 | 0.73 |
